# Genome Stability of Lyme Disease Spirochetes: Comparative Genomics of *Borrelia burgdorferi* Plasmids

**DOI:** 10.1371/journal.pone.0033280

**Published:** 2012-03-14

**Authors:** Sherwood R. Casjens, Emmanuel F. Mongodin, Wei-Gang Qiu, Benjamin J. Luft, Steven E. Schutzer, Eddie B. Gilcrease, Wai Mun Huang, Marija Vujadinovic, John K. Aron, Levy C. Vargas, Sam Freeman, Diana Radune, Janice F. Weidman, George I. Dimitrov, Hoda M. Khouri, Julia E. Sosa, Rebecca A. Halpin, John J. Dunn, Claire M. Fraser

**Affiliations:** 1 Department of Pathology, University of Utah School of Medicine, Salt Lake City, Utah, United States of America; 2 Department of Medicine and Institute for Genome Sciences, University of Maryland School of Medicine, Baltimore, Maryland, United States of America; 3 Department of Biological Sciences, Hunter College of the City University of New York, New York City, New York, United States of America; 4 Department of Medicine, Health Science Center, Stony Brook University, Stony Brook, New York, United States of America; 5 Department of Medicine, New Jersey Medical School, Newark, New Jersey, United States of America; 6 J. Craig Venter Institute, Rockville, Maryland, United States of America; 7 Biology Department, Brookhaven National Laboratory, Upton, New York, United States of America; University of Kentucky College of Medicine, United States of America

## Abstract

Lyme disease is the most common tick-borne human illness in North America. In order to understand the molecular pathogenesis, natural diversity, population structure and epizootic spread of the North American Lyme agent, *Borrelia burgdorferi* sensu stricto, a much better understanding of the natural diversity of its genome will be required. Towards this end we present a comparative analysis of the nucleotide sequences of the numerous plasmids of *B. burgdorferi* isolates B31, N40, JD1 and 297. These strains were chosen because they include the three most commonly studied laboratory strains, and because they represent different major genetic lineages and so are informative regarding the genetic diversity and evolution of this organism. A unique feature of *Borrelia* genomes is that they carry a large number of linear and circular plasmids, and this work shows that strains N40, JD1, 297 and B31 carry related but non-identical sets of 16, 20, 19 and 21 plasmids, respectively, that comprise 33–40% of their genomes. We deduce that there are at least 28 plasmid compatibility types among the four strains. The *B. burgdorferi* ∼900 Kbp linear chromosomes are evolutionarily exceptionally stable, except for a short ≤20 Kbp plasmid-like section at the right end. A few of the plasmids, including the linear lp54 and circular cp26, are also very stable. We show here that the other plasmids, especially the linear ones, are considerably more variable. Nearly all of the linear plasmids have undergone one or more substantial inter-plasmid rearrangements since their last common ancestor. In spite of these rearrangements and differences in plasmid contents, the overall gene complement of the different isolates has remained relatively constant.

## Introduction

Bacteria in the spirochete genus *Borrelia* cause arthropod-borne human diseases such as Lyme disease and relapsing fever, as well as a number of diseases of veterinary importance [1]–[6]. They are obligate parasites that are only found in their vertebrate or arthropod hosts and are rather difficult to study in the laboratory. Only quite recently have their biology, genetics and molecular pathogenesis begun to become accessible to experimentation [7]–[9]. The determination and analysis of the first *Borrelia* genome sequence, that of the *Borrelia burgdorferi* type strain B31, stimulated significant progress in this arena. Its unusual genome was found to comprise a 910 Kbp linear chromosome and twenty-one (twelve linear and nine circular) plasmids that contain over 600 Kbp of DNA [10], [11] (two additional plasmids are now thought to have been lost between the isolation of strain B31 and its genome sequence determination [12], [13]). This work confirmed Barbour's [14] original observations, and many other studies have shown that *Borrelia* isolates universally harbor numerous linear and circular plasmids (*e. g.*, [15]–[27]). The B31 chromosome carries 815 predicted genes (our re-annotation, below) that encode largely housekeeping functions. These functions include a minimal metabolic capability that cannot synthesize amino acids, nucleotides or lipids *de novo* [11].

One circular plasmid, cp26, carries genes that encode several nucleotide metabolism enzymes [28], small molecule transporters [29], [30] and the enzyme that creates the unique closed hairpin telomeres present on the *Borrelia* linear replicons [31]–[34]. The other plasmids have very few metabolic or housekeeping genes, but do encode numerous lipoproteins, many of which have been shown to be present on the cell surface when they are expressed (*e. g.*, [35]–[37] and references therein). The plasmids have a number of interesting features in addition to bearing lipoprotein genes. (i) A number of the linear plasmids have an unusually low protein coding density for prokaryotic DNA and carry numerous pseudogenes that appear to be in a state of genetic decay [10], [38]. (ii) Several of the circular plasmids in strain B31 (the cp32s) are homologous nearly throughout their lengths [10], [12]. (iii) There are unusually large numbers of paralogous genes on the plasmids. The vast majority of strain B31 plasmid genes have plasmid-borne paralogs, and in strain B31 107 of the paralogous gene families (PFams) include mostly plasmid genes. (vi) The highly paralogous nature of the plasmids, along with the apparent mutational decay of some members of PFams, suggests a history of duplicative rearrangements followed by decay of damaged and redundant genes [10], [38]. (v) Up to eleven of the B31 plasmids appear to be prophages or are prophage-related [39]–[41]. (vi) Most of the plasmids, probably all but cp26, can be lost without affecting growth in culture (*e. g.*, [42], [43]). (vii) And finally, several plasmids have been shown to be required for growth in mice or in *Ixodes* ticks, and/or encode proteins that interact with host components (details below). Thus, the plasmids appear to be largely involved in the interactions of *Borrelia* with its hosts.

All members of the *Borrelia* genus that have been analyzed carry linear chromosomes that are similar in size to the strain B31 chromosome. These chromosomes appear to be quite evolutionarily stable, since their sizes do not vary greatly and recent sequences of the chromosomes of additional Lyme agent *B. burgdorferi* sensu stricto species [44] and related species *B. garinii*, *B. afzelii*, *B. “bavariensis”*, *B. “finlandensis”*, *B. valaisiana*, *B. spielmanii* and *B. bissettii* [45]–[49], show that they are all essentially co-linear with the chromosome of *B. burgdorferi* B31, and that there are only a very small number of chromosomal gene content differences among these species (with the exception of *B. burgdorferi* extreme right-end differences [50], [51] and the larger but still relatively modest differences between Lyme agent and relapsing fever *Borrelia* species [52]). Directed analyses have shown that *B. burgdorferi* plasmids cp26 [27], lp54 [20] and the cp32s [12] have largely conserved structures and are present in all isolates that have been studied. Other plasmids appear to have conserved structures but are only present in a subset of strains (*e. g.*, B31-like cp9 [10], [53], [54] and lp38 [21]), while still others such as lp5, lp21, lp36, and lp56 are less frequently present and/or have highly variable sizes and presumably variable structures [19], [21], [55], [56]. The similar sizes of different plasmids (which are not separable in electrophoresis gels) and the highly paralogous nature of the plasmids has made unambiguous assembly and analysis of plasmid sequences complex and difficult [10], [47]. Thus, studies of bacteria in the *Borrelia* genus are in an unenviable position in which determination of all the plasmids present in any new isolate requires that a complete (non-draft) genome sequence be determined.

Comparison of whole genome nucleotide sequences both within and between species is a powerful and critical part of gaining a true understanding of the organization, diversity and evolution of bacterial genomes. This strategy reveals the invariant features of the compared genomes and allows discovery of more variable sequences that (i) correlate with specific host disease features, (ii) permit tracking of sub-types within species, and (iii) give critical insight into evolutionary mechanisms. In addition, comparison of closely related genomes can often illuminate inaccuracies in the prediction of genes and other features in genomes. In this report we discuss the plasmids present in the *B. burgdorferi* genomes of isolates N40, JD1 and 297 and compare their genetic contents and organizations with the previously known strain B31 genome. More global and less gene oriented comparisons of the twenty-two *B. burgdorferi* sensu lato genomes that we have sequenced [10], [11], [44]–[46], [49] will be presented in subsequent publications.

## Results and Discussion

### *B. burgdorferi* whole genome sequence determination

In order to begin to address questions about *B. burgdorferi* and population structure, the genetic basis of virulence, possible exchange of genetic information among individual bacteria in the wild, as well as natural diversity and evolutionary mechanisms of the Lyme disease *Borrelia* species, we determined and annotated the complete sequences of the whole genomes of *B. burgdorferi* strains N40 and JD1 and of the plasmids of strain 297 [44]. These strains and strain B31 were chosen for the analysis presented here because (i) space prevents such a detailed analysis of many more isolates, (ii) they include the three most commonly used laboratory strains (B31, N40 and 297), (iii) they represent four different lineages by rRNA spacer sequence [57]–[60], pulsed-field gel DNA pattern [16], [17], [61], OspC (outer surface protein C) [62]–[65] and multilocus sequence type [66]–[69] (Table 1), and, importantly, (iv) the accuracy of the computer assembly of the plasmid sequences has only been confirmed experimentally for only these four isolates. Strain 297 was isolated from a human with Lyme disease in CT, while JD1, N40 and B31 are *Ixodes scapularis* tick isolates from MA, NY and NY, respectively. All four come from the northeastern part of United States of America, a region with a high frequency of human Lyme disease.

**Table 1:** *B. burgdorferi* isolates in this study.

Isolate	PFGE type^a^	rRNA IGS1 lineage^b^	OspC type^c^	MLST type^d^	Number of plasmids		Biological source	Ref.
					Linear^e^	Circular^e^		
B31	B	1	A	1	12	11^f^	*Ixodes* tick/NY	[70]
N40	E	9	E	19	9^f^	8	*Ixodes* tick/NY	[71]
JD1	C	5	C	11	11	9	*Ixodes* tick/MA	[72]
297	A	2	K	3	10^f^	10	Human/CT	[73]

a Numerous *B. burgdorferi* chromosomal pulsed-field gel electrophoresis (PFGE) types have been identified (*e. g.*, [16], [17], [61].

b At least nine *B. burgdorferi* rRNA intragenic spacer (IGS) lineages have been defined [57], [58], [74].

c Over 20 *B. burgdorferi sensu stricto* OspC types have been identified [57], [62].

d Multilocus sequence typing (MLST) of Margos *et al.* [67].

e Number of plasmids present (the values for N40, JD1 and 297 were determined in this study and for B31 by Casjens *et al.* [10]).

f Includes the following plasmids known to have lost from the cultures whose genomes were sequenced: B31, cp9-2 and cp32-5; 297, lp25; and N40, lp28-3 (see also text and footnote c of table 2).

Whole genomic DNAs from *B. burgdorferi* strains N40 and JD1 and isolated plasmid DNA from strain 297 were sequenced by previously described Sanger sequencing random shotgun and closure methods (Materials and Methods). In each genome, DNA library “shotgun” sequencing followed by closure of sequence gaps by sequencing PCR amplicons or additional DNA clones resulted in the accumulation of multiple, unconnected sequence contigs. Our previous experience with the *B. burgdorferi* B31 genome suggested that such contigs most likely represent the large chromosome and the multiple plasmids that all *Borrelia* cells carry, and that repeated sequences on plasmids can cause incorrect sequence assembly. To confirm that the shorter contigs are plasmid-derived and to check the correctness of the assembly of the sequencing runs into contigs, whole cell DNAs, uncleaved and cleaved with strategically chosen restriction enzymes, were displayed in pulsed-field electrophoresis agarose gels and analyzed by Southern hybridization using unique probes prepared by PCR amplification from each of the putative plasmids contigs in N40, JD1 and 297 (data not shown). This restriction mapping ensured that improper assemblies were corrected, that the linear or circular nature of each plasmid was independently determined, and that the plasmid sequences accurately reflect the true *in vivo* situation. Since the covalently closed, hairpin-ended terminal fragments from the *Borrelia* linear replicons do not ligate into the vectors used to make circular plasmid sequencing libraries [10], [11], sequence determined in this way is expected to be missing some bp from each end of the linear replicons. In the process of confirming the sequence assembly, the native plasmid sizes and sizes of terminal restriction fragments from most linear plasmid ends were measured, and the approximate number of unsequenced bp at the DNA ends were estimated; these values are given in table S1. The nucleotide sequence currently determined for the plasmids of *B. burgdorferi* strains B31, N40, 297 and JD1 are 612108, 437361, 508697 and 608486 bp, and represent about 40%, 33%, 40% and 37% of these total genome sequences, respectively. The accession numbers of the chromosome and plasmids of these four strains were reported previously [10], [11], [44](and are also listed in table S1). Some preliminary and/or specific gene findings regarding these genome sequences at their incomplete “draft” stages have been reported elsewhere [66], [75]–[79].

During this work we found that the strain “N40” *ospC* gene sequence deposited in GenBank under accession number AF416430 is in fact *not* from the authentic N40 which was originally isolated and described by Barthold *et al.* [71] and whose genome we sequenced (the correct N40 *ospC* sequence has been previously deposited in GenBank three times under the accession Nos. U04240, DQ437463 and AY275221). We have confirmed that the “AF416430 strain”, which we call strain “M” in this report (see below), is still masquerading as “N40” in some laboratories (see [80] for additional details), and caution is recommended in assuming that the N40 genome sequence discussed here is from the same isolate as all strains previously reported under “N40” name.

### The *B. burgdorferi* sensu stricto chromosome

#### The chromosome “constant portion”

Restriction mapping, anecdotal sequencing, and DNA array analysis have indicated that the chromosomes of different isolates of *B. burgdorferi* sensu stricto are quite similar (*e. g.*, [17], [61], [66], [67]). The genome sequences show that the B31, N40 and JD1 chromosomes are indeed essentially completely syntenic, with the only major length variation among *B. burgdorferi* sensu stricto chromosomes being different amounts of plasmid-like DNA attached at their right ends (see below). The “constant region” includes the left 903 Kbp that carries strain B31 genes *b31_0001* through *b31_0843* (see Materials and Methods for gene nomenclature). B31-JD1, B31-N40 and JD1-N40 pair wise comparisons show that the constant regions of the chromosomes are 99.5%, 99.4% and 99.4% identical, respectively.

In an effort to improve the accuracy of the annotation of the *B. burgdorferi* genes, we annotated the JD1, N40 and 297 genome sequences in parallel, and updated the B31 genome annotation, as described in Materials and Methods. ORFs ≤50 codons were removed from the prediction, and those in the 51–100 range were not predicted unless they were intact and had homologs in all four of the genome sequences discussed here; two putative chromosomal genes, *b31_0771a* and *b31_0838a*, were identified that were not recognized in the original annotation of the B31 chromosome. Many of the short ORFs were previously suspected to be spurious gene identifications or nonfunctional genes [10], and these are not included in the present analysis unless they meet the above criteria. We thus identify 815 putative chromosomal protein-coding genes which occupy 93.5% of the 903 Kbp constant region in the B31, N40 and JD1 chromosomes. These genes as well as the tRNA, tmRNA and rRNA genes are all present and in identical locations in all three *B. burgdorferi* chromosomes, and there are no large indels or rearrangements among the three sequenced chromosome constant regions. The constant regions of the chromosome will be compared in detail elsewhere and will not be discussed further in this report.

The *B. burgdorferi* chromosomes have significant gene content differences only in the variable region at their right ends. We previously identified three different lengths of extensions beyond the right end of the constant region in a panel of 31 isolates from North America [17], [50], [51]. Strain N40 represents a chromosome that has no long “extra” DNA extension at its right end, and its rightmost gene (*n40_0843*, which encodes a probable arginine-ornithine antiporter) is less than two hundred bp from the right telomere by our measurement of terminal restriction fragment lengths (data not shown). B31, 297 and JD1, have additional sequences that extend about 7, 19 and 20 Kbp beyond the N40 right end position [51]. The B31 extension is “plasmid-like” in that it contains only genes that are similar to those on B31's plasmids [10]. Strain 297's right end is extremely similar to that of strain Sh-2-82, and much of the latter's chromosome extension was determined to be 99% identical in sequence to the B31 linear plasmid lp21 [51]. The exact sources of the B31 and JD1 extensions were not known.

**Figure 1:**
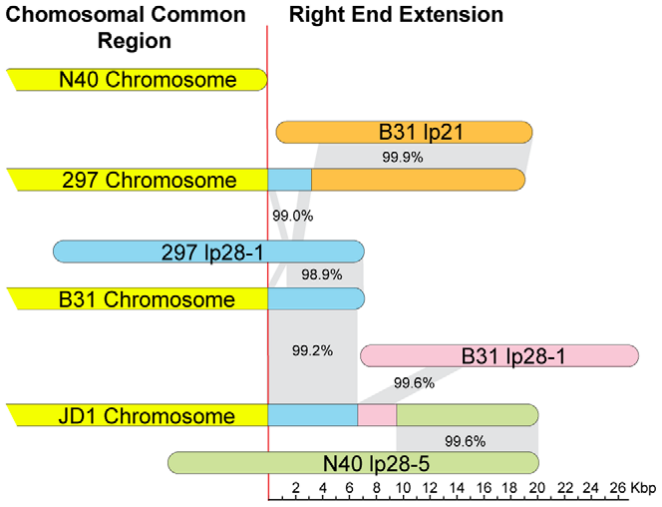
Length variation of *B. burgdorferi* chromosomes. The relationships among the right end, plasmid-like, chromosomal extensions relative to known plasmids are indicated by gray shading; plasmid sizes are not drawn exactly to scale. There is a 1053 bp deletion and a 17 bp insertion in the 297 lp28-1 plasmid relative to the B31 extension. It is assumed that the 297 chromosome is essentially identical to that strain Sh-2-82 (see text and [51]).

The B31 chromosome extension can now be identified as >98% identical to the version of linear plasmid lp28-1 that is present in strain 297 (plasmid nomenclature is discussed in the following section), and three contiguous sections of the JD1 extension are 99.2%, 99.6% and 99.6% identical to sections of 297 lp28-1, B31 lp28-1, and N40 lp28-5 linear plasmids, respectively (Figures 1 and S1). The extremely high similarity between the chromosomal extensions and these plasmids strongly supports the notion that these chromosomal right-end extensions and linear plasmids have had very recent common ancestors. The mechanism of joining plasmid sequences to the chromosome is not known. It does not appear to be fusion at the telomeres, since all the plasmid-like sequences at the chromosome ends are not near the ends of plasmids except the right chromosomal telomere, which appears to be the plasmid telomere when there is an extension. We have argued previously that, since (i) closely related species such as *B. bissettii*, *B. garinii* and *B. afzelii* do not appear to have such chromosomal extensions, and (ii) genes are not likely to be rebuilt perfectly by non-homologous recombination events, these represent accretions of DNA onto the chromosome from linear plasmids and thus represent a mechanism by which *Borrelia* recruits plasmid genes onto the chromosome [10], [50], [51].

### Plasmid types, occurrence and nomenclature

#### Plasmid content of sequenced genomes

Our results show that the sequenced cultures of *B. burgdorferi* strains B31, N40, JD1 and 297 carry 12, 8, 11 and 9 linear plasmids and 9, 8, 9 and 10 circular plasmids, respectively. Thus, these isolates each carry between 16 and 21 plasmids. Table 2 lists the plasmids present in each genome, and table S1 gives their experimentally measured and sequence contig sizes. With the updated re-annotation of the B31 genome described above, the number of annotated genes on sequenced plasmids is 706, 463, 677 and 585 in B31, N40, JD1 and 297, respectively. A difficulty in working with *Borrelia* strains in the laboratory is that plasmids are often spontaneously lost, and, although there is evidence that strain 297 carries a homolog of B31 linear plasmid lp25 [81], [82] and N40 carries a homolog of lp28-3 [78], these two plasmids were not present in the genome sequences determined in this study. They must have been lost between the original isolation and growth of the culture for DNA isolation and genome sequencing; strain B31 is also known to have lost two or three plasmids before sequencing [12], [13]. The bp position numbers of the linear plasmids of B31 used in this report are those of the original GenBank annotations of Casjens *et al.* [10] that do not include the more recently determined terminal sequences reported by Tourand *et al.* [83].

**Table 2:** *B. burgdorferi* plasmids present in four isolates.

	B31	N40	JD1	297
Linear plasmids	12	8	11	9
Circular plasmids	9	8	9	10
Total plasmids	21	16	20	19
lp5	+	−	−	−
lp17	+	+	+	+
lp21	+	−	−	−
lp25	+	+	+	(+)^a^
lp28-1	+	−	+	+
lp28-2	+	+	−	−
lp28-3	+	(+)^a^	+	+
lp28-4	+	+	+	+
lp28-5	−	+	+	+
lp28-6	−	−	+	+
lp28-7	−	−	+	−
lp36	+	+	+	+
lp38	+	+	+	+
lp54	+	+	+	+
lp56	+	−	−	−
cp9-1	+	+	−	−
cp9-2	(+)^a^	−	−	−
cp26	+	+	+	+
cp32-1	+	−	+ (fused)^b^	+
cp32-3	+	−	+	+
cp32-4	+	+ (trunc)^b^	−	+
cp32-5	(+)^a^	+	+ (fused)^b^	+
cp32-6	+	−	+	+
cp32-7^c^	+	+ (trunc)^b^	−	+
cp32-8	+	−	+	−
cp32-9	+	+	+	+ (trunc)^b^
cp32-10	+ (int)^b^	+	+	−
cp32-11	−	−	+	+
cp32-12	−	+	+	+

a Plasmids known to be in some cultures of the indicated strain, but which were not present in the sequenced culture. Their sequences remain undetermined.

b Structural differences from otherwise organizationally similar plasmids are indicated as follows: trunc, truncated compared to other homologous plasmids; int, B31 cp32-10 is integrated into plasmid lp56; fused, JD1 cp32-1 and cp32-5 are fused into one large circular “cp32-1+5” plasmid.

c In some B31 cultures the plasmid cp32-7 is replaced by cp32-2 [12]. These two plasmids have the same apparent compatibility and appear to be prophage DNAs. Since it seems unlikely that they can exist in the same cell, and they are expected to be able to move between strains, one of them may have been inadvertently introduced in the laboratory. We use cp32-7 for this compatibility type since it is the one that is present in the completely sequenced B31 genome.

#### *B. burgdorferi* plasmid types and nomenclature

*Borrelia* plasmids were originally named according to their DNA topology (linear “lp” and circular “cp”) and approximate size in Kbp. Strain B31 linear plasmid lp54, for example, is 53678 bp in length. To continue to name the plasmids in all strains according to their size, however, has several difficulties: (i) a majority of the linear plasmids are in the 24 to 30 Kbp size range, so different names based only on size are limited, (ii) we find numerous significant organizational and size differences among the strains (*e. g*., the “lp36's” present in the four strains considered here range from 23 to 36 Kbp in length; see below), and (iii) such names have no biological significance. To give plasmids names that correlate with at least some biological feature, we [75], [77] and Stevenson and Miller [84] have suggested that, when possible, names be given to *Borrelia* plasmids according to their type of partitioning genes, and in particular the type of paralogous family (PFam) 32 protein that they encode (PFams defined by Casjens *et al.* [10]; see also below). The PFam32 proteins are homologs of the ParA plasmid partitioning proteins in other better understood systems [85], [86], and experiments in *Borrelia* indicate that PFam32 protein “sequence types” correlate with plasmid compatibility types (see references cited in Casjens *et al*. [75], [77]). The PFam32 proteins encoded by the four genomes fall into twenty-six easily distinguishable sequence types, fourteen on linear plasmids and twelve on circular plasmids [75]. Additional compatibilities include plasmids cp9 and lp5, which carry no PFam32 gene and so cannot be categorized in this way. The compatibility properties of lp5 and cp9 are not understood; no lp5-like plasmid is present in the other isolates analyzed here, and it is not known if the cp9's in B31 and N40 are compatible (see below).

In the three new genome sequences three new linear plasmid PFam32 types are identified that are not present among the previously known B31 plasmids. These are named “lp28-5” (present in strains N40, JD1 and 297), “lp28-6” (JD1 and 297) and “lp28-7” (JD1) (Table 2). The sizes of these six new “lp28” linear plasmids range between 27 and 31 Kbp. We chose “lp28-X” names, since they carry genes that are largely from the same set of PFams as the four “lp28” plasmids present in strain B31. In addition, two PFam32 types not present in B31 are found among the circular plasmids of N40, JD1 and 297. Stevenson and Miller [84] independently discovered both of these types and named them cp32-11 and cp32-12.

### Plasmid variation among *B. burgdorferi* strains

#### Plasmid organizational and gene content variation

Comparison of strains B31, N40, JD1 and 297 reveals major structural differences in a number of the plasmid PFam32 types, in addition to complete plasmid presence-or-absence differences. There appear to have been numerous rearrangements between and within plasmids since their last common ancestors. These rearrangements are largely restricted to the linear plasmids and result in a patchwork or mosaic relationship when two “cognate” (in same PFam32 group) plasmids are compared. A given plasmid can have long patches of very high sequence similarity (often several Kbp >99% identical) with a plasmid in another strain, and yet have immediately adjacent sequences that are different in the two plasmids and that are either (i) nearly identical to part of a different plasmid, (ii) homologous to, but less highly related to sections of other plasmids, or (iii) not present in the other three strains analyzed here. Such *inter-strain* linear plasmid relationships are reminiscent of the relationships among the linear plasmids *within* each individual strain, and we previously argued from analysis of the strain B31 plasmids that the intra-strain mosaic relationships were apparently generated by duplicative rearrangements and perhaps also horizontal transfer processes [10], [38]. The presence of such inter-strain differences agrees with previous studies which have shown that particular plasmid sequences (used as hybridization probes) are not present on identically sized plasmids in all *B. burgdorferi* isolates (*e. g.*, [19], [21]). Because of these complex relationships, in this report we use a conservative definition of “orthologous” to mean identical syntenic positions on plasmids of the same compatibility type, and *not* for the most closely related genes when two strains are compared or for genes that lie in small regions of synteny on different plasmid types.

**Figure 2:**
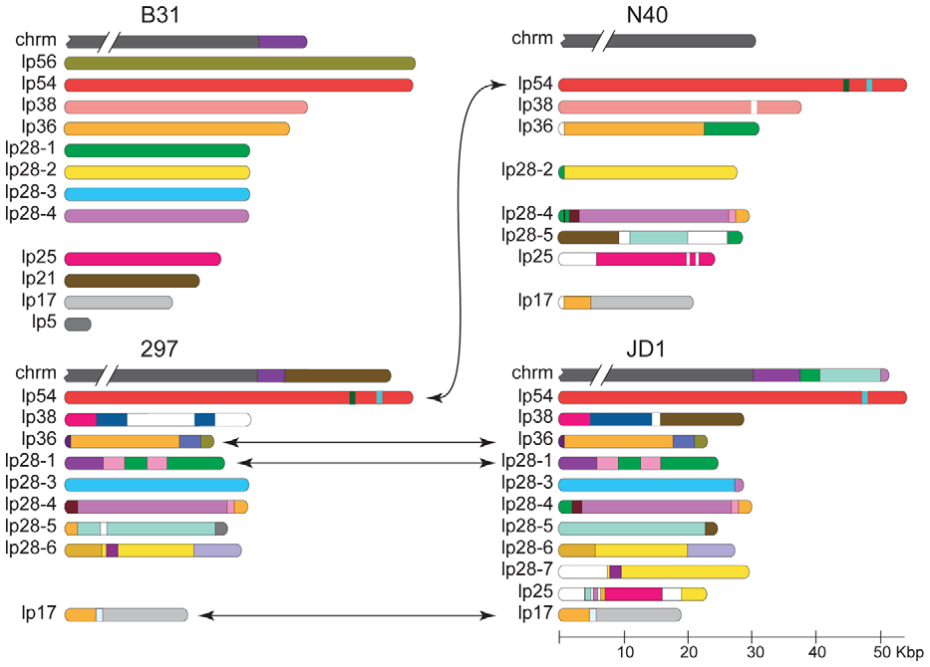
Linear plasmid contents of *B. burgdorferi* strains B31, N40, 297 and JD1. The linear plasmids and right end plasmid-like chromosomal extensions of are shown as horizontal bars with rounded ends. Identical colors indicate regions of nucleotide sequence that is ≥94% identical, and white denotes regions that are <94% identical to other sequences in the diagram. Each of the B31 plasmids was first defined with a different color and additional colors were added to the other plasmid sets as necessary. Arrows connect plasmids that have identical overall organization and high sequence similarity (ignoring small polymorphisms and indels <500 bp). Strain 297 plasmids lp28-3 and lp28-4 have not been sequenced to their termini (see table S1), so it is not known whether they are organizationally the same as their B31 or JD1 and N40 or JD1 cognates, respectively.

Figure 2 shows a diagrammatic depiction of the differences in organization among the linear plasmids of strains B31, N40, JD1 and 297 in which identical colors represent nucleotide sequences that are >94% identical in the different genomes. (The >94% cutoff is arbitrary; however, we note that essentially all of the locally syntenic and thus orthologous regions are >94% identical among these strains. If lower cutoffs are used many more regions that are homologous but not orthologous would be given the same color in the figure.) It is evident from this overview that although a few organizationally identical linear plasmids are found in the different strains, none of the linear plasmid PFam32 types are organizationally identical across the four strains. Linear plasmids, lp54, lp28-2 and lp28-3 have relatively small (∼1 Kbp or less) differences among the four strains, but lp17, lp25, lp28-5, lp36 and lp38 each have three very different versions that have significantly different gene contents. There is relatively little strain-specific DNA that is unique to any of the four strains; *i.e.*, only a very small fraction of any of these genomes' total linear plasmid sequence is unrelated to sequence present somewhere in the other three sequenced *B. burgdorferi* genomes; we note that nearly all of the white regions in Figure 2 actually have homology to plasmid sequences in one of the other strains, but it is less than the 94% identical cutoff used in the figure. The following sections discuss the inter-strain relationships of each plasmid type in more detail.

### Variation within each plasmid type in different *B. burgdorferi* isolates

#### *cp9*

B31 cp9 is not required for mouse or tick infection in the laboratory and is rather easily lost in culture [87]–[89]. Cp9 plasmids have been previously sequenced from *B. burgdorferi* strains B31 [11] and N40 [53] and *B. afzelii* strain IP21 [54] and found to be rather similar, but not identical in these three cases (see figure 6 in [77]). Of the new genomes compared here, only N40 carries a cp9 plasmid, and it is very similar to B31 cp9-1. Our 8722 bp N40 cp9 sequence has seven single bp differences from the previously reported N40 cp9 sequence [53]. These two plasmids have identical gene organizations, except that one putative gene (*b31_c10* or *revB*) and part of another gene (*b31_c08*) of B31 cp9-1 are replaced in N40 cp9 by several hundred bp of apparently non-protein-coding DNA (Figure S2B). The cognate B31 and N40 cp9-1 genes range from 73.8% to 99.1% identical. These plasmids carry no PFam32 gene, but they do encode PFam57 proteins that are about 90% identical, and members of this family have been shown to be required for proper plasmid replication/partitioning [90]. It is not known if these two plasmids are compatible.

#### cp26

Circular plasmid cp26 is universally present in *B. burgdorferi* isolates (*e. g.*, [27], [55]), is required for virulence in mice [42], [91], and is the only plasmid that is known to be essential for growth in culture [27], [91]–[94]. The B31 cp26 carries genes involved in GMP synthesis [28], chitobiose import [30], host integrin binding [95], oligopeptide import [29], and the telomere hairpin formation [32], [33]. It also encodes one of the important surface antigens expressed in the mammalian host, OspC protein [57], [96]–[100]. The three additional complete cp26 sequences in the genomes analyzed here all have identical gene content and organization to the B31 cp26 reported by Fraser *et al.* [11] (Figure S2C). The *ospC* genes are especially variable and range from 82.6% to 86.5% identical in the four strains. If *ospC* is removed from the comparison, the remainder of the plasmids range from 98.4% to 99.2% identical in pair wise comparisons, close to the average similarity of the chromosomes (∼99.4% identity, above). A more detailed analysis of single-nucleotide polymorphisms in cp26s will be reported elsewhere (E. Mongodin, W. Qiu, B. Luft, S. Schutzer, C. Fraser-Liggett and S. Casjens, unpublished).

#### cp32s

Members of this family of circular plasmids are present in all *B. burgdorferi* isolates analyzed to date and are thought to be prophages [39]–[41]. In addition to putative phage virion assembly genes, the cp32s carry genes that encode a number of proteins that have been studied [35], [101]. These phage “lysogenic conversion” (host modification) genes include the *rev* genes whose surface lipoprotein products bind fibronectin [102], the *mlp* encoded surface lipoproteins [103], [104], the *bdr* (*Borrelia* direct repeat) genes whose functions are unknown, and the complex family of the *erp* (also called *ospEF* or *elp*) genes whose various members have been shown encode surface lipoproteins that bind to plasminogen [105], laminin [106] and factor H complement regulatory factor binding protein [107]–[109].

The sequenced genomes of B31, N40, JD1 and 297 contain nine, six, nine and nine members of the cp32 family, respectively. Like the B31 cp32s, the cp32 plasmids in the other three strains are homologous throughout nearly their entire lengths, and the 23 new complete cp32 sequences all have gene arrangements that are very similar to those of the B31 cp32s (ORF maps of these plasmids are shown in Figure S2D–G). Among the thirteen known cp32 compatibility types [84], only plasmids with cp32-9 partition genes are present in all four genomes (if the cp32-5 which was lost before the B31 genome was sequenced [10], [12] is included, then it too was present in all four isolates).

The variations in overall organization of these cp32s include the integration of one into a linear plasmid in B31 [10], two are fused into one large “cp32-1+5” plasmid in JD1 that is made up of two different full-length cp32s fused together to form a 60.7 Kbp circular plasmid (this fusion of cp32-1 and cp32-5 plasmids was confirmed by Southern DNA restriction enzyme cleavage analysis, data not shown), two in N40 are truncated (cp32-4 and -7, which have approximately 14 and 13 Kbp deletions, respectively) and two in 297 are truncated (cp32-7 and -9, which both have ∼9 Kbp deletions) (Figures S2E and G); several of these deletions have been noted previously, where N40 cp32-7 was called cp18, and 297 cp32-7 and cp32-9 were called cp18-1 and cp18-2, respectively [84], [110], [111]. In addition, there is an approximately 5.6 Kbp inversion in N40 cp32-5 (Figure S2E). The four deletions and the inversion affect only the putative virion assembly gene region of these prophage plasmids [40], [41], so the plasmid partitioning and lysogenic conversion genes of these plasmids appear to remain intact. There are also a small number of other gene content differences among the cp32s, such as the presence or absence of a *revA* gene (*e. g.*, the complement of cp32s in JD1 carries no *revA* gene) and several variably present genes immediately transcriptionally downstream of the *erp* gene region. Only two putative gene types are present in the newly sequenced cp32s that do not have homologues on cp32 plasmids in the B31 genome; these are *297_w45* (a PFam55 gene; members of this family are present on four linear plasmids and cp9-1 in B31) in 297 cp32-11, and *jd1_q42* and *297_m41* found on JD1 cp32-10 and in 297 cp32-7, respectively (a fragment of this gene family lies at the same location in B31 cp32-3). In addition, more than one *mlp* gene is present in JD1 cp32-12 and 297 cp32-4. The *erp/elp//ospEF* gene group diversity will be discussed in detail elsewhere (B. Stevenson, B. Jutras & S. Casjens, unpublished). There appears to have been considerable homologous recombination among the cp32 plasmids, and this is discussed in more detail below.

#### lp5, lp21 and lp56

The N40, JD1 and 297 genome sequences do not contain plasmids with partition genes similar to B31 lp5, lp21 or the non-cp32-like portion of lp56. However, we have previously found that a 16 Kbp region that is very similar to B31 lp21 sequences is present at the right end of the strain 297 chromosome [51], and we note below that JD1 lp38 also carries a section that is very similar to a major part of B31 lp21.

#### lp17

**Figure 3:**
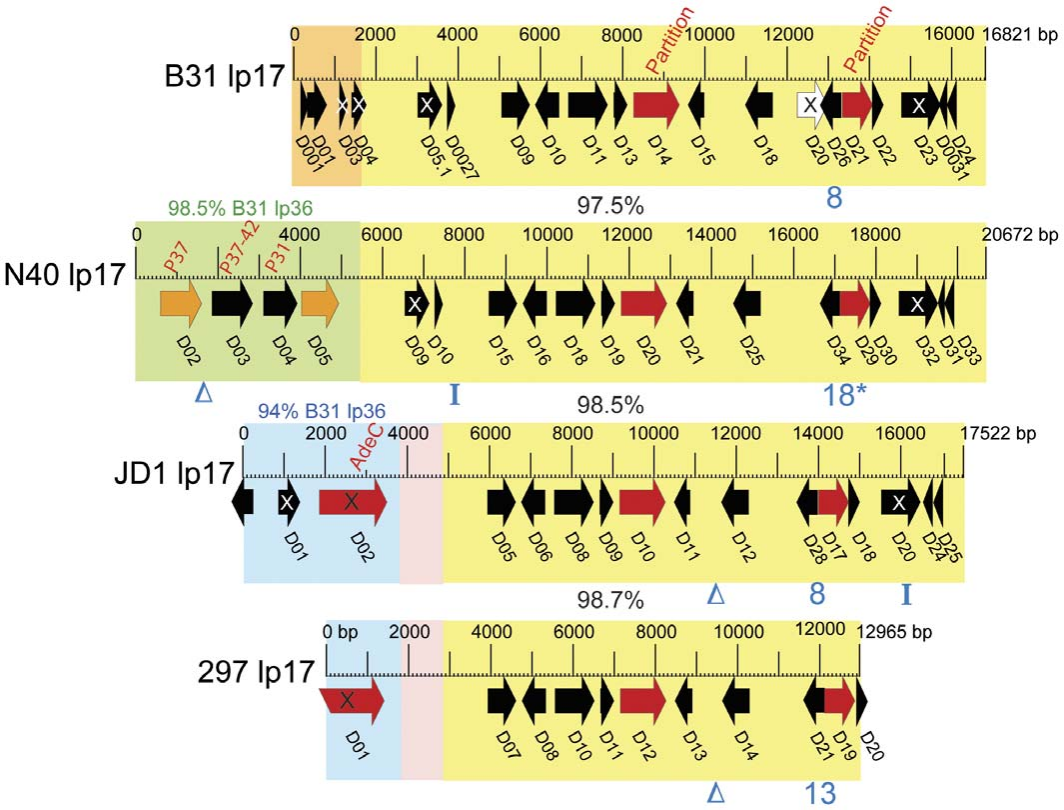
Organizational and open reading frame relationships among four lp17 plasmids. The four lp17s are aligned vertically, and identical colored background rectangles indicate very similar sequence (the 297 plasmid is the same size as JD1 lp17 but is missing several Kbp of sequence from its termini; see text and table S1). Rectangles of the same color denote homologous sequences, and the percentage nucleotide sequence identity of parallel yellow sections are shown between the maps. The arrows denote annotated predicted genes, where red arrows have a predicted function, black have unknown function, orange are known antigens, and white is a pseudogene not annotated except in the B31 plasmid; alternate gene names or predicted function are noted in red text in figure. An “X” indicates that a gene is truncated or has a frame disruption relative to a known homolog. The blue “Δ” indicates a short deletion relative to orthologous sequence in another lp17 plasmid(s); blue numbers indicate the number of short tandem repeats present at that location; an asterisk (*) notes that the repeat sequence is not identical to that of the other lp17s; an (I) marks the locations of short inversions relative to the other lp17s.

Lp17's roles in pathogenesis are unclear, but it encodes protein D18 that regulates OspC expression from cp26 (above) [112]. There are three organizationally different versions of lp17 in the four genomes, the previously characterized 17 Kbp B31 plasmid, a 21 Kbp N40 plasmid, and 19 Kbp plasmids in JD1 and 297 (Figure 3). The rightmost ∼13 Kbp regions of these four plasmids are >97% identical; however, they have three very different left ends as follows: (i) N40 lp17 carries four apparently intact genes that are >98% identical to B31 lp36 genes *b31_k45*, *b31_k46*, *b31_k47* and *b31_k50*; orthologs of these four genes are not present in the JD1 or 297 plasmid sequences, although other members of their paralogous families are present. Fikrig *et al.* [113] showed that the *n40_d02* (the *b31_k50* homolog on lp17) protein elicits protective immunity in mice. N40 does not contain orthologs of *b31_k48* and *b31_k49*, and it seems likely that the *b31_k48* and *b31_k49*-like genes were removed from the N40 lp17 by a homologous recombination event between *b31_k47* and *b31_k49*-like genes in a progenitor in which this region resembled B31 lp36 in organization. Xu *et al.'s* [114] PCR amplifications suggested that their set of OspC type E strains all have a similar lp17 arrangement to that of N40. (ii) To the left of their homology with B31 lp17, the very similar JD1 and 297 lp17s have a short 200 bp sequence that is about 84% identical to a fragment the PFam145 genes of the cp32 plasmids, about 900 bp that are not similar to any other sequence in these four genomes, and about 3.7 Kbp that are 94% identical to genes *b31_k13*, *b31_k15* and *b31_k17* (*adeC*) of B31 lp36. The *b31_k15* and *b31_k17* homologs in JD1 and 297 have frame-disrupting mutations, but there are intact versions of both on lp36 in JD1 and in 297. (iii) Finally, the left end of B31 lp17 has 1496 bp that is unique to B31 and contains no gene of known function.

The 2–3% difference between the orthologous sequences on these plasmids (Figure 3) does not affect the reading frames of any of the putative lp17 genes; however, there have been a few small rearrangements in the different lineages. As an example of the types of such differences that are present between orthologous plasmid sequences in strains B31, N40, JD1 and 297 in general, these are indicated in Figure 3. They include deletions (identical 241 bp deletions relative to the other two at about bp 11100 in JD1 and 9100 in 297), inversions (of 259 bp at bp ∼8000 in N40 and of 101 bp in the *d20* pseudogene in JD1), and differing numbers of the tandem 21 bp repeats present in *b31_d20* and its orthologs (8, 18, 8 and 13 copies in B31, N40, JD1 and 297, respectively). All of the repeats in N40 lp17 have the same two single bp differences relative to the other lp17 plasmids, perhaps implying substantial contraction and expansion of the array since the N40 sequence has been evolutionarily separated from the others.

#### lp25

This linear plasmid in strain B31 has been shown to be essential for infection of mice and ticks [81], [89], [115], [116], and it harbors several important genes, *pncA* (*b31_e22*), which encodes a nicotinamidase [117], *bptA* (*b31_e16*, a surface lipoprotein that is required for persistence of strain 297 in the tick vector [82]), and *b31_e02*, a member of PFam01 that encodes a DNA restriction/modification protein whose presence lowers the efficiency of genetic transformation of B31 cells [118]–[120]. N40 and JD1 lp25s each carry genes that are orthologs of *pncA* and *bptA*, and a homolog of *b31_e02*. Although infectious strain 297 carries an lp25-like plasmid [81], [82], it was unfortunately lost from the culture whose genome was sequenced.

**Figure 4:**
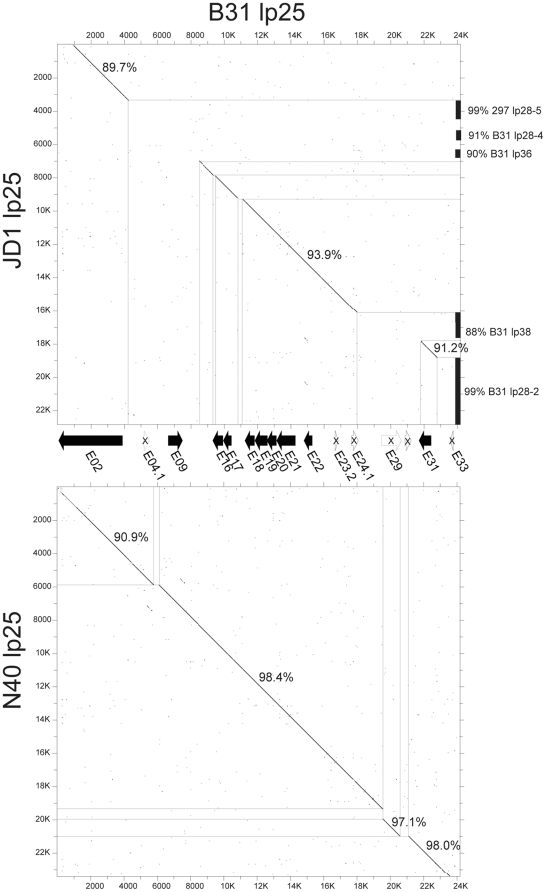
Comparison of three lp25 plasmids. Matrix plots with a 19 identities/23 bp window were created by DNA Strider [135]. Percent identities of nucleotide sequences are indicated near the diagonal identity line for most orthologous regions. The predicted genes for B31 lp25 are shown between the two plots (open arrows with “X”s are putative pseudogenes), and regions of high similarity to other plasmids are noted on the right.

Figure 4 shows that the N40 lp25 is very similar to B31 lp25 with only a small number of short indel differences in regions that are not predicted to affect intact genes. The rightmost 18 Kbp regions of the two plasmids are over 98% identical, while the leftmost approximately 6 Kbp is about 91% identical, and a few hundred bp at the extreme left ends are unrelated in the B31 and N40 lp25 plasmids. JD1 lp25 represents a different subtype of this plasmid. The central 9 Kbp of JD1 lp25 is more divergent, with about 94% identity and two several hundred bp indels relative to the B31 and N40 lp25s. JD1's intact *bptA* and *pncA* genes lie in this region, and it also has a PFam01 restriction/modification gene at its left end and a PFam60 putative lipoprotein gene in the right central region that are about 90% identical to the orthologous genes in this position in B31 and N40 lp25. Finally, JD1 lp25 has several sections that are not present in the B31/N40 type lp25s; these “mosaic” patches have 98.6% and 98.8% identity to parts of 297 lp28-5 and B31 lp28-2, respectively, as well as weaker similarities to other *B. burgdorferi* plasmids (Figures 4 and S2H). These latter differences mean that JD1 carries no true ortholog to the antigenic B31 E09 (PFam44) putative lipoprotein, and carries only members of PFam52 (*jd1_e04*) and PFam102 (*jd1_e27*) that are rather divergent from their B31 counterparts.

#### lp28-1

**Figure 5:**
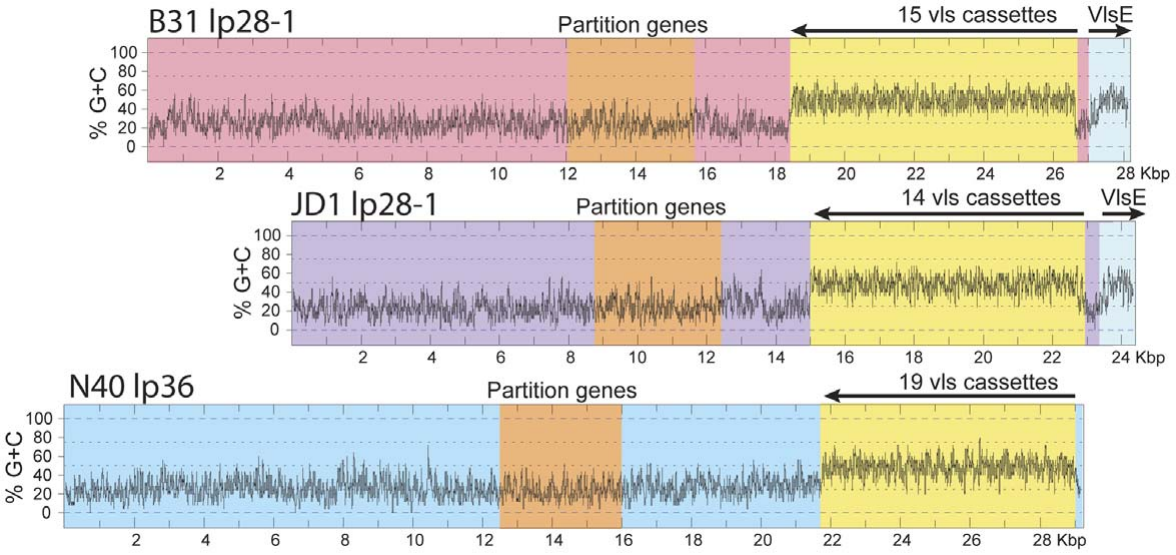
Comparison of lp28-1 plasmids and the *vls* cassette and *vlsE* loci. Percent G+C plots for the plasmids were created by DNA Strider [135]. Different background color indicates very different sequence in the different plasmids (note that the partition gene regions in the two lp28-1 plasmids are homologous, but moderately divergent from those of lp36; see text).

The B31 plasmid lp28-1 has received considerable attention because it carries two genes, *arp* (*b31_f01)* and *vlsE* (near the left and right ends, respectively), that are important in the mouse model of Lyme borreliosis. Loss of lp28-1 severely reduces strain B31 infectivity in mice but not in ticks [81], [89], [115], [116], [121]–[124], and antibodies against the B31 Arp protein cause resolution of *B. burgdorferi* induced arthritis in mice [125]–[127]. N40 does not carry a plasmid with an lp28-1 PFam32 gene, and the JD1 and 297 lp28-1 plasmids are very similar to each other (99.5% identical over the nearly 15 Kbp), but are quite different from B31 lp28-1. These two lp28-1 types only have the partition genes and *vls/vlsE* region in common (Figures 5 and S2I). Between these two regions of the JD1 and 297 plasmids lies about 2.6 Kbp of DNA that contains a PFam106 gene (*jd1_f23* and *297_f25*) that is homologous to B31 lp38 genes *b31_j23* and *b31_j24*. This JD1 protein is only about 40% identical to B31_J23 protein. At their left ends JD1 and 297 lp28-1s have about 6 Kbp that is about 99% identical to the right end extension of the B31 chromosome (above) and which contains an apparently intact PFam138 gene (*b31_0852* in B31) and about 2.7 Kbp that has no ortholog in B31 and no convincing intact genes. The *arp* gene is not present on lp28-1 in the three new genomes discussed here. In N40 it is near the right end of lp28-5 (gene *n40_y16*), and in JD1 it is near the left end of lp28-4 (*jd1_i37*; and perhaps also in 297, although the sequence of the parallel region of its lp28-4 was not determined). The B31 and N40 Arp proteins are identical, and the JD1 homolog is 99.1% identical to them, so this movement of the *arp* gene among these different plasmids happened quite recently.

The *vlsE* gene at the right end of B31 lp28-1 encodes a major outer surface protein and is unique in *Borrelia* in that during a mouse infection genetic information from fifteen tandem, unexpressed *vls* cassettes can be copied into the *vlsE* expression locus, presumably to present the host immune system with a “moving target” which can in theory have millions of different amino acid sequences [124], [128]–[132]. The *vlsE* gene was not present in the DNA libraries used in the original sequencing of the strain B31 genome, and similarly was not present in any of the libraries used in sequencing the three genomes presented here. Zhang *et al*. [128] cloned the B31 *vlsE* expression locus and showed that it lies very close to the right telomere of lp28-1 in strain B31. More recently, Hudson *et al.* [133] and Bykowski *et al.* [134] have shown that there are seven bp (5′-TTCTCTC; see accession No. DQ275473) between the bulk of the lp28-1 sequence (accession No. AE000794) and the *vlsE* expression locus sequence (accession No. BBU76405). Possible *vlsE* expression loci sequences for strains 297 and N40 have been PCR amplified (297, accession Nos. U76405 and AB011063; N40, X. Wang and J. Weis, personal communication), but attempts at PCR amplification between these sequences and the N40 and 297 cassette regions from N40 or 297 DNA were unsuccessful. However, similar PCR amplification using primers designed from the right end of the JD1 lp28-1 sequence cassette region and the reported 297 expression locus allowed extension of the JD1 lp28-1 sequence to include most of its *vlsE* locus. The sequence of the *vls-vlsE* region will be examined in more detail elsewhere (S. Norris, D. Edmundson, T. Lin, G. Chaconas and S. Casjens, unpublished).

The *vls* cassette region is also present on plasmids with lp28-1 compatibility in B31, JD1 and 297, but in N40 they reside on the plasmid that has an lp36 type PFam32 gene. In the N40 plasmid, the joint between B31 lp28-1 and B31 lp36-like sequences is close to the left end of the cassettes, so little other B31, JD1 or 297 lp28-1-like genetic material is present on N40 lp36. Like the parallel region in B31, the *vls* cassette region of N40, JD1 and 297 all have extremely high (for *Borrelia*) G+C contents of about 50% (Figure 5), and B31, JD1 and N40 each have a dip to a much lower and more nearly normal *Borrelia* G+C content between the cassettes and the *vlsE* expression site near the right end of the plasmid (the 297 sequence does not extend this close the plasmid's right end). The presence of this G+C dip and a lack of similarity with the *vlsE* gene near the right end of the N40 lp36 sequence suggest that all of the N40 cassettes are likely represented in the reported sequence. Although the cassette regions are similar in size (about 8, 7.2 and 8 Kbp, respectively, in B31, N40 and JD1), there are significant differences between these cassette regions. B31 has 15 cassettes, JD1 has 14 and N40 has 19. The B31 and JD1 cassettes are quite constant in size (about 570 bp with a few that are up to 90 bp shorter; Figure S3). The N40 cassettes are somewhat more variable in size and range from about 200 to over 600 bp in length (average is 395 bp; Figure S3). The cassette sequences are much more divergent than all other clearly orthologous sequences in these isolates. The 6 Kbp of the JD1 and 297 cassette regions that are sequenced in both genomes contains eleven cassettes that are up to 93% identical, but numerous less similar sections make the whole region only about 81% identical between the two strains. All the other pair wise comparisons of the four cassette regions are less similar (*e. g.*, about 65% between B31 and JD1, based on alignments created by DNA Strider [135] and by inspection of diagonal matrix similarity plots, but it is difficult to obtain accurate sequence alignments). Thus the *vls* cassettes are present in these four strains in three approximately equidistantly related versions represented by B31, N40 and JD1/297.

#### lp28-3

Plasmids with lp28-3 type PFam32 genes are present in B31, JD1 and 297. N40 has been reported to carry such a plasmid [78], but it was absent from the N40 culture whose DNA was sequenced. These three plasmids are very similar (over 99% identical in pair wise comparisons; Figure S2J), except in JD1 where about 1.2 Kbp at the right end contains a PFam52 gene (*jd1_h47*) that is not homologous to the parallel region of B31 lp28-3 (where a PFam48 gene lies in this region); the 297 lp28-3 sequence does not extend near enough to the right end, so it is not known if it is similar to JD1 or B31 in this regard. The JD1 1.2 Kbp right end (above) is 99.5% identical to sequence at the right end of B31 lp36. The only B31 lp28-3 gene that has been studied, the *cspZ* gene (*b31_h06*) encodes the human factor H complement regulatory factor binding protein CRASP-2 [78], [136] and lies in the common region of the three lp28-3s.

#### lp28-4

The B31 lp28-4 is required for the ability to infect the tick gut [116], and it carries several genes of current interest including *b31_i06* which encodes a surface localized nucleotidase [137], *b31_i16* (*vraA*; variable strain-associated repetitive antigen A) which confers partial protection as a vaccine [138], *b31_i26* which encodes a possible multidrug efflux protein, and *b31_i38* and *b31_i39* which encode PFam54 surface proteins [37], [139]. The four lp28-4s are very similar (99.2 to 99.9% identical in pair wise comparisons) over most of their lengths, but the B31 plasmid has several Kbp of different, non-homologous DNA at both ends (Figure S2K). The right terminal 2 Kbp of the N40 and JD1 plasmids are nearly identical to each other and are 99.3% identical the left terminus of B31 lp36; this region harbors a PFam12 (*jd1_i36*) gene and a PFam01 fragment (*jd1_i47*) that are not present on B31 lp28-4. The leftmost 1.4 Kbp of B31 lp28-4 is replaced in the JD1 and N40 plasmids by different B31 lp28-1-like sequences; in the JD1 plasmid this DNA contains the *arp* gene (see above).

Although the central orthologous regions of the four lp28-4s are very similar, there are a few differences that could have significant effects on the function of possibly important genes on these plasmids. The *jd1_i19* (putative multidrug efflux pump) has an in-frame stop codon relative to the other three strains, and B31 and JD1 PFam60 orthologs *b31_i28* and *jd1_i22* have different single frameshifts relative to the cognate N40 and 297 genes. Finally, B31 lp28-4 has a set of three tandem paralogous PFam54 genes (*b31_i36*, *b31_i38* and *b31_i39*), while the other three lp28-4s have two such genes in this position. Since the B31 genes *b31_i36* and *b31_i38* are 99.4% identical, it seems likely that these are the result of a recent gene duplication in the B31 lp28-4 lineage. Thus, although the orthologous lp28-4 regions are over 99% identical, several mutational changes have occurred in them that could have functional importance.

#### lp28-5

**Figure 6:**
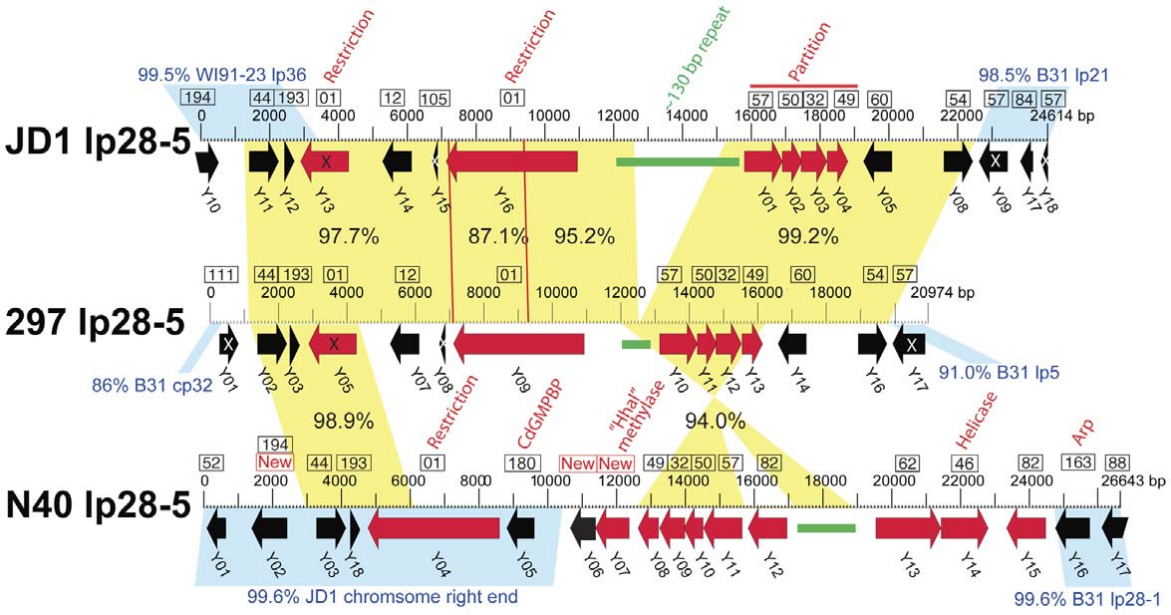
Organizational and open reading frame relationships among three lp28-5 plasmids. Maps are labeled as in Figure 3. Yellow background between maps joins regions of homologous sequence in adjacent maps; paralogous family numbers (table S2) are indicated in black boxes above each putative gene; red boxes marked “new” indicate genes for which there is no homolog in the strain B31 genome; green bars mark the 133 bp repeat regions (see text); CdGMPBP, cyclic-di-GMP binding protein. Blue background marks regions of high similarity to regions in other *B. burgdorferi* genomes.

N40, JD1 and 297 each carry a plasmid with a previously unknown type of PFam32 gene that has been named lp28-5 (Figure 6). These represent a new *Borrelia* plasmid PFam32 compatibility type (see PFam32 tree in figure 3 of [75]). A substantial fraction of N40 lp28-5 is not *closely* related to any of the previously characterized B31 *B. burgdorferi* plasmids; nonetheless, it largely encodes more distant homologs of known B31 genes. The JD1 and 297 lp28-5 partition proteins are 94% identical to their N40 orthologs. Like most of the other linear plasmids, the three lp28-5s also have significant differences. The JD1 and 297 lp28-5 plasmids are quite similar to one another and carry genes that are mostly homologs of the B31 paralogous families (*e. g.*, PFams01, 12, 54, 60; Figure 6); however, they have only the partition gene cluster and one additional 3.4 Kbp section in common with N40 lp28-5. The common 3.4 Kbp regions (>97% identical) contain a PFam44 gene (*jd1_y11*, *297_y02* and *n40_y03*) and a fragment of a PFam01 gene. Each of the three lp28-5 plasmids also carries between 6 and 26 tandem repeats of a 133 bp sequence of unknown function (see below).

There appear to have been a number of recent inter-plasmid DNA transfer events that involved the lp28-5s. The rightmost 2.1 Kbp of N40 lp28-5 is 99.6% identical to the left end of B31 lp28-1 (and contains an *arp* gene; above), while the right ends of JD1 and 297 lp28-5s are different from that of N40 and one another; N40 lp28-5's best matches are 98.5% identical to the left end of B31 lp21. The JD1 lp28-5 leftmost 4.4 Kbp is 99.5% identical to a portion of the lp36 found in strain WI91-23 [44]. N40 lp28-5's leftmost 10.5 Kbp is 99.6% identical to the right end of the JD1 chromosome (above), but the left ends the JD1 and 297 lp28-5s are not highly similar to any sequences in the other genomes compared here. The 297 lp28-5 left end contains a short ∼200 bp section that is 86% identical to part of the *blyB* (*b31_r24*) gene of B31 cp32-4. Sequences that appear to have been transferred between *Borrelia's* circular and linear plasmids are not common, but in addition to this instance, we find that JD1 lp28-7 (below) carries two *bapA/eppA* family (PFam95) genes that are typically found in some cp32s and cp9-1s.

**Figure 7:**
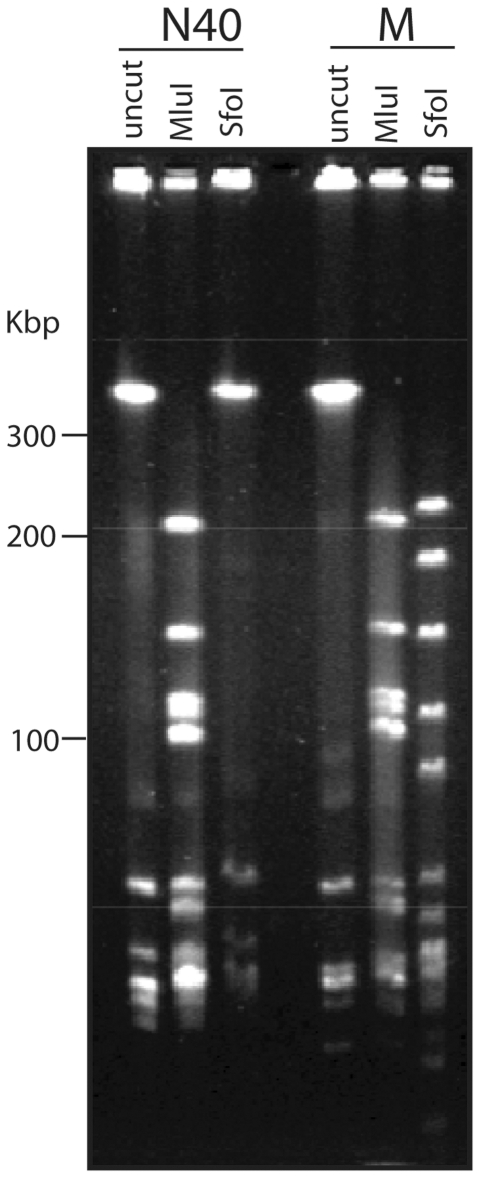
N40 DNA is not cut by restriction endonuclease *Sfo*I. DNAs were prepared in agarose blocks, cleaved with the indicated restriction endonuclease, and subjected to agarose gel pulsed-field agarose electrophoresis and stained with ethidium bromide as previously described [17], [147]. Strain M is described in the text. Identical results to those with strain M were obtained with strains B31, JD1 and 297 (data not shown).

N40 lp28-5 also carries the following predicted genes which have no B31, JD1 or 297 plasmid homologs: (i) *n40_y02* which encodes a novel putative lipoprotein, (ii) *n40_y05* which is predicted to encode a cyclic-di-GMP-binding protein [140], [141] and is a homolog of a chromosomal gene that is present in all sequenced chromosomes (*e. g.*, *b31_b0733/plzA*), (iii) *n40_y14* which is a putative helicase, (iv) *n40_y06* which has no known homologs, and (v) *n40_y07* whose predicted protein product is 48% identical in amino acid sequence to HhaI cytosine DNA methyltransferase encoded by *Haemophilus haemolyticus* ([142] and references therein). To test the predicted *in vivo* HhaI-like DNA methyltransferase activity of the N40_Y07 protein, we treated *B. burgdorferi* DNAs with restriction endonucleases SfoI and HhaI (New England Biolabs, Ipswich, MA), since HhaI cytosine methyltransferase methylates GCGC to create GC^me^GC, and DNA cleavage by both SfoI and HhaI are blocked by this methylation [143]. Endonuclease cleavage was monitored by CHEF pulsed field gel electrophoresis display of the resulting fragments. Figure 7 shows that *Sfo*I fails to cleave N40 DNA, while it does cleave the DNA of strains M (above), B31, JD1 and 297 (data not shown for the last three). The same is true for restriction endonuclease HhaI (data not shown), suggesting that the *n40_y07* encodes an active cytosine DNA methyltransferase with the above specificity.

#### lp28-2, lp28-6 and lp28-7

Plasmids of these three compatibility types are considered together because, although they comprise three different PFam32 types, they are otherwise quite similar (Figure S2L). Near the left end of all five of these plasmids are partition gene clusters whose PFam32 proteins are of three different types (see tree of PFam32 proteins in figure 3 of [75]). We named these two new types lp28-6 and lp28-7. All five of these plasmids have long central syntenic regions that include several genes with homology to bacteriophage virion assembly genes (typified by *b31_g21*, terminase; *b31_g20*, portal protein; *b31_g10* tail tape measure protein), suggesting that this region may be the virion assembly operon of a prophage [40], [144]. B31 and N40 carry mostly syntenic lp28-2 plasmids that are 96.1% identical in their homologous regions but carry a few hundred bp of non-homologous sequences at their extreme left ends. The central 12 Kbp regions in the other three plasmids have >99% identity punctuated with shorter stretches of less similar but still homologous sequences. For example, JD1 and 297 lp28-6 central regions are perfectly syntenic and 99.5% identical except for a 1.8 Kbp patch of homology with about 60% identity (Figure S2L).

At the right ends of these five plasmids are 7 to 8 Kbp that are homologous, but are less similar than the central region; here the lp28-6s of JD1 and 297 are over 99% identical, but they are only 75–85% identical (with several indels) to the parallel portion of JD1 lp28-7 and the two lp28-2s in Figure S2L. These five plasmids carry three different types of lipoprotein genes at their extreme left ends; (i) B31 and N40 lp28-2 have PFam12 (*e. g.*, *b31_g01*) and PFam102 (*b31_g02*) putative lipoprotein genes whose roles are not known. (ii) JD1 lp28-7 encodes two *bapA* (PFam95) proteins AA37 and AA38 that are about 50% identical to previously known members of this family [87], [145], [146]. (iii) The leftmost gene on JD1 lp28-6, *jd1_z01*, is a rather distantly related homolog of the B31 *vlsE* gene. It is less similar to the JD1 lp28-1 *vls* cassettes than is the JD1 *vlsE* gene, so it seems that it has not recently procured genetic information from the *vls* cassettes (which are present on lp28-1 in JD1, above); it is about the same length as *vlsE* and appears to have an intact lipidation amino acid sequence consensus. The other three *B. burgdorferi* sequences do not contain an intact gene of this type (B31 and N40 have related *b31_j51* and *n40_j34* pseudogenes at their lp38 right termini); the terminal portion of the possibly syntenic lp28-6 plasmid in 297 was not sequenced.

A surprising feature of the JD1 genome sequence determination is that its lp28-6 was *very* highly represented in the DNA libraries used for sequencing. While the remainder of the genome coverage was in the 9- to 30-fold range (including the other plasmids), lp28-6 sequence coverage was 180-fold, indicating an approximately 10-fold higher copy number than the other *Borrelia* plasmids, which are present in the 1 to 3 per chromosome range when it has been measured [147], [148]. This feature is not a general property of the lp28-6 type plasmids, since the very closely related 297 lp28-6 was not over-represented. Interestingly, the JD1 and 297 lp28-6s have extremely similar partition gene clusters; there are only three differences between the nucleotide sequences of this region, single bp differences just upstream of the PFam62 gene (*jd1_z06*) and in the PFam50 gene (*jd1_z05*) that do not change the amino acid sequence of the encoded proteins, and a different number of AAAGAA repeats within the PFam49 gene (Figure S4). There are six tandem copies of this sequence in the JD1 gene (*jd1_z03*) and eight in the 297 ortholog (*297_z01*). It is possible that the upstream mutations affect expression levels or that the altered number of repeats (each of which encodes Lys-Glu) affects the function of the PFam49 protein to increase the copy number.

#### lp36

**Figure 8:**
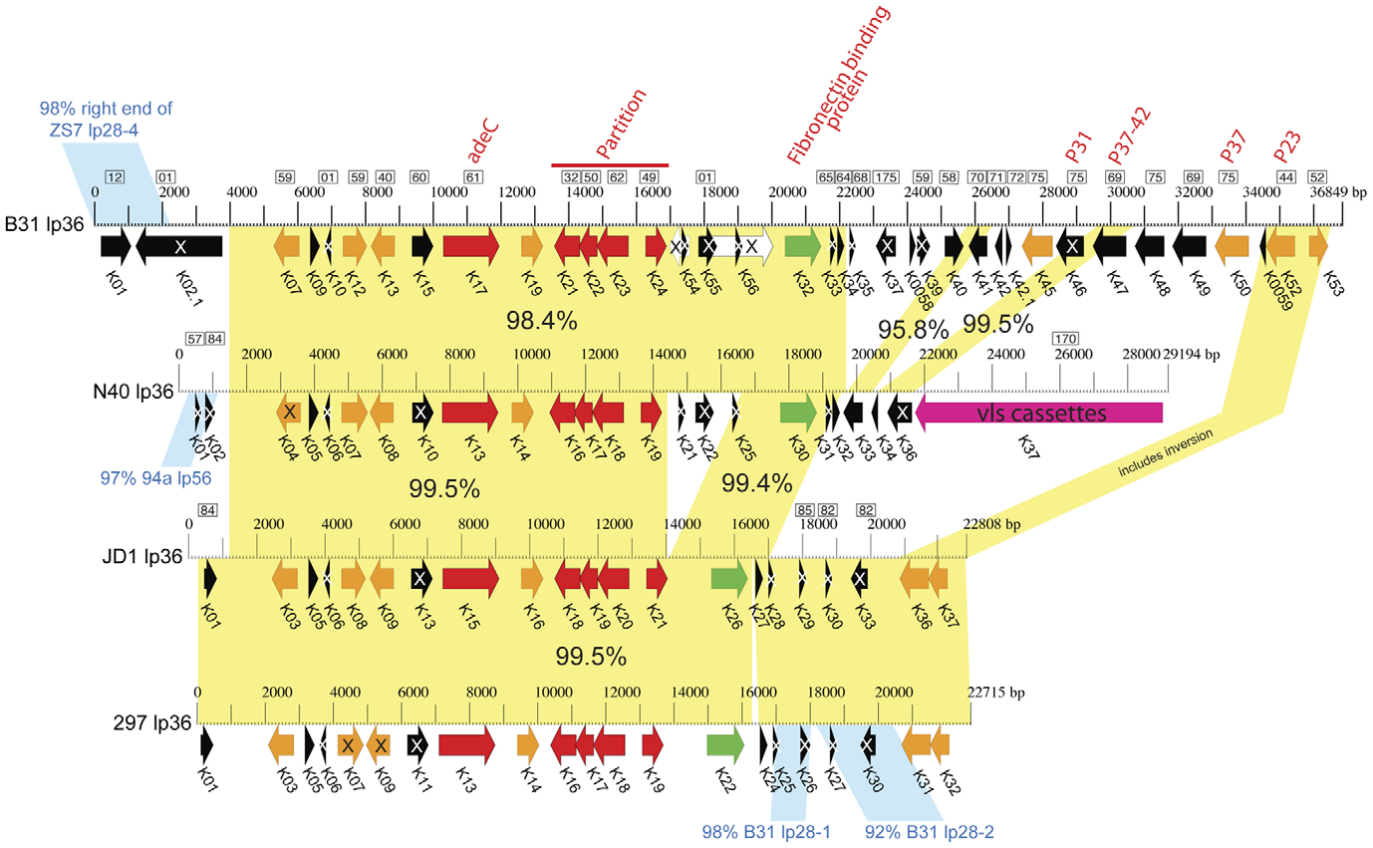
Organizational and open reading frame relationships among four lp36 plasmids. Maps of the four lp36s are labeled as described in Figure 6; green genes encode predicted and proven surface lipoproteins; red, plasmid partitioning and other DNA and nucleotide metabolism proteins; magenta, and *vls* cassette region (see text). Yellow shading between maps marks regions of nucleotide sequence similarity (percent identity values in black text).

B31 lp36 carries two genes whose products have been studied, an adenine deaminase encoded by *b31_k17 (adeC)* that is important in mouse infectivity [149] and a fibronectin binding protein encoded by *b31_k32* [150]–[154]; both of these are present on the lp36 plasmids of all four strains compared here. In addition, lp36 encoded lipoprotein K07 is an immunodominant antigen in human infection [155]–[157], and its gene is also present in all four lp36s; however, the N40 ortholog (*n40_k04*) has a frame disrupting mutation. B31 lp36 was previously known to be unusual in that DNA probes from it hybridize with plasmids that are particularly variable in size in different strains [21]. The lp36 plasmid sequences from B31, N40, JD1 and 297 were measured in agarose gels to be 36, 31.5, 24 and 24 Kbp long, respectively (Table S1; data not shown). The JD1 and 297 lp36's are 99.5% identical, with only one indel where 234 bp are missing at bp 16524 of the JD1 plasmid; no long ORF is affected by this indel (Figure 8). These two plasmids are shorter versions of B31 lp36 in which about 4 Kbp of the B31 plasmid's left end is replaced by about 1 Kbp of sequence that is 87% identical to the B31 lp28-4 left end, and sections of about 2 and 12.5 Kbp of B31 lp36 are not present in the JD1 and 297 plasmids (Figure 8); in the latter two, the 12.5 Kbp region is replaced by about 4.5 Kbp of which 3 Kbp is closely related to part of B31 lp28-1. N40 lp36 also has left end differences from the other three lp36 plasmids, and as discussed above, the N40 lp36 carries the *vls* cassette region (on lp28-1 plasmids in the other three strains) at its right end (Figure 8). Thus, the lp36 plasmids appear to have undergone several major rearrangements since their last common ancestor and are present as three quite different organizational subtypes in the four strains analyzed here.

#### lp38

**Figure 9:**
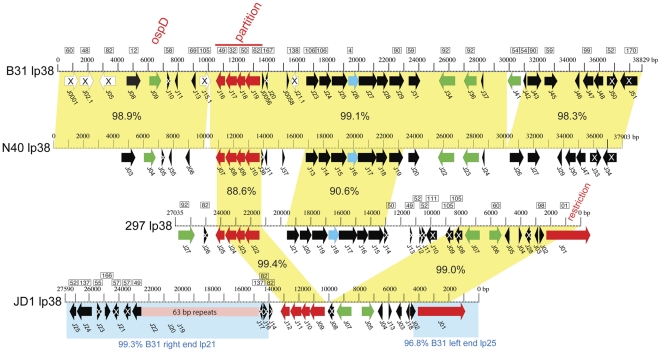
Organizational and open reading frame relationships among four lp38 plasmids. Maps of the four lp38s are labeled as described in Figure 8. Yellow shading between maps marks regions of high nucleotide sequence similarity (percent identity values in black text). The pink horizontal bar indicates the region of 63 bp repeats in JD1 lp38; and blue arrows represent predicted transporter genes.

Highly related linear plasmids in the 37 to 39 Kbp size range are present in 25 of the 56 *B. burgdorferi* sensu stricto isolates that have been examined [19], [21], [158], [159], but it is not required for the tick-mouse infectious cycle in the laboratory [160]. Where it has been studied these ∼38 Kbp plasmids carry parallel sets of genes (which include the outer surface protein OspD gene, *b31_j09* [21], [158], [159], [161], [162]). This suggested a potentially invariant organization for these plasmids [21]. B31 and N40 are among the strains previously known to carry a plasmid in this size range, and 297 and JD1 are among those known not have one [17], [21], [159], and the sequences reported here confirms those findings. The N40 genome contains a linear plasmid (37903 sequenced bp) that is very similar to B31 lp38 (Figure 9). The B31 and N40 lp38 plasmids are about 99% identical in nucleotide sequence, with only three indels larger than 25 bp as follows: (i) B31 is missing 351 bp at its bp 9571 within a PFam115 pseudogene (*b31_j15.1*), (ii) at bp 10293 of B31 lp38 there are 12 tandem repeats of the heptamer AATAGTT (between genes *b31_j15.1* and *b31_j16*), whereas in the N40 sequence it is repeated 119 times, and (iii) N40 is missing 1093 B31 bp at its bp 30326 that includes most of genes *b31_j41* and *b31_j42* (*b31_j41* is a PFam54 gene that has been shown to encode a membrane protein [37]).

Plasmids that carry lp38-related PFam32 partition genes are nonetheless present in the JD1 and 297 genomes, but these plasmids are quite different from those in B31 and N40 (Figure 9). Although the N40/B31 and JD1/297 lp38 type PFam32 protein sequences are robustly clustered, they form two distinct subgroups within this branch of the PFam32 tree (see figure 3 in [75]), and it is not known if these differences could give rise to compatibility differences. JD1 and 297 lp38's were measured by Southern pulsed-field gel analysis to be about 29 and 31 Kbp in length, respectively (data not shown), and both have rather complex relationships to other known *Borrelia* plasmids. In addition to nearly identical 3 Kbp regions that contain the four gene partitioning cluster, 297 and JD1 lp38s contain about 8 Kbp of common sequence; of this 8 Kbp about one third is 97% identical to the left end of B31 lp25 (Figure 9), and the remaining part has no very closely related homolog present elsewhere in these four genomes. The latter region contains (i) genes (*297_j06* and *jd1_j05*) that encode closely related PFam60 putative lipoproteins that are only ∼55% identical to their closest relative in B31, Q05 protein, and (ii) genes *jd1_j07* and *297_j07* which have no B31 homolog and encode closely related putative lipoproteins that are ∼75% identical to plasmid-encoded proteins PGP088 and BAPKO_6042 of *B. garinii* PBi and *B. afzelii* PKo, respectively [46]–[48]. In addition, JD1 lp38 contains about 13 Kbp at its left end that is >99% identical to the right half of B31 lp21 (including 7.8 Kbp of the lp21 63 bp tandem repeat tract). The 297 lp38 carries ∼9 Kbp of DNA (in three patches) that has no very closely related sequence elsewhere in the four strains (Figure 9); however, it encodes, for example, a 71% identical homolog of the PFam92 *b31_j27*. Neither JD1 nor 297 carries (on lp38 or elsewhere in the genome) a homolog of the B31 *ospD* gene.

#### lp54

Previous studies have shown that plasmids similar in size and gene content to B31 lp54 are universally present in *B. burgdorferi* sensu lato isolates (*e. g.*, [17], [19]–[22], [55], [92]). The B31, N40 and 297 lp54s are known to encode the well-studied outer surface proteins OspA and OspB, as well as decorin binding proteins, a thymidylate synthase, the complement factor H binding protein CRASP-1, and a number of less well-characterized surface and antigenic proteins (*e. g.*, [163]–[169]). Some of these have been found to be important in the tick-mouse infectious cycle [169]–[182]. The three new lp54 sequences all have gene contents that are nearly identical to B31 lp54 (Figure S2M), and have overall sequence identities between 98.9% and 99.4% (excluding the few differences discussed below). The only translational reading frame-breaking difference found among the genes of the four lp54s is a stop at codon 207 of the *ospB* gene in strain 297 (*297_a16*). Curiously, Probert *et al.* [183] found a stop codon in the 297 *ospB* at codon 199. Apparently these two subcultures of 297 have suffered independent *ospB* mutations. These may have been selected in different laboratory mouse passages, since OspB loss has been reported to be involved in interaction with host immunity [184].

Analysis of the lp54 sequences indicated that the homologs of *b31_a24* and the *b31_a68-b31_a70* cluster are the most variable parts of this plasmid [66] (in the lp54s of the four strains considered here, orthologous genes have identical locus_tag numbers). B31 lp54 carries a cluster of nine tandemly arranged PFam54 genes of which *b31_a64*, *b31_a65*, *b31_a66*, *b31_a68*, *b31_a69*, *b31_a70* and *b31_a73* are apparently intact, and *b31_a71* and *b31_a72* are truncated. Among these genes only the function of B31_A24 and B31_A68 (CRASP-1) proteins have been studied, and they are required during tick-to-mouse transmission [171], [175] and bind human complement factor H [166], respectively. N40 and 297 lp54s are missing the *b31_a70* ortholog and have a novel member of the family (called *n40_a67.5* and *297_a67.5*, respectively) inserted into the cluster. JD1 is also missing a *b31_a70* ortholog but has no additional gene. These relationships and their evolutionary significance were discussed in more detail in Wywial *et al.* [76]. The putative deletion that removed the *b31_a70* ortholog in JD1 has different endpoints from the one that removed it in N40 (which is identical to that of 297), suggesting that independent deletions occurred in these strains. The N40 deletion appears to have been generated by a homologous recombination event between similar paralogous sequences but, curiously, the JD1 deletion appears to have been a non-homologous event (see below). The other highly sequence-variable location on the lp54s includes the region orthologous to the 3′-terminal portion of *b31_a24* (which encodes decorin binding protein B [185], [186]) and the *b31_a23-b31_a24* intergenic region, where B31, JD1 and N40 have quite different sequences, but JD1 and 297 have more closely related sequences. This latter relationship is similar to the larger picture in which the 297 and JD1 linear plasmids are organizationally the most closely related strain pair (summarized in Figure 2); however, the close relationship between N40 and 297 in the *n40_a68-n40_a73* region does not agree with this history, and suggests that this rearrangement (if it happened only once in this exact fashion, which seems likely) has moved horizontally relative to other parts of lp54.

### Plasmid genes and paralog families

#### Paralogous protein families

The sequence of the strain B31 genome indicated that its genome contains a large number of paralogous gene families that lie largely on the plasmids [10], and this is also true of strains 297, N40 and JD1. Our analysis of the PFams in each strain and, where possible, the orthology relationships among the strains within PFams are presented in table S2 (see Materials and Methods and legend of table S2 for methods used). This analysis points out that divergence and rearrangements can make the true inter-strain orthology relationships of genes on the *Borrelia* plasmids difficult to discern (*e. g*., PFam01 example below).

This analysis found 160 paralogous families in the four genomes analyzed here (note that the PFam numbers go higher than 160 because some originally defined PFams have subsequently been merged under one number). The large majority of these PFams have members in all four strains. Of the 109 plasmid gene-containing PFams, only seventeen do not have representatives present in all of the four genome sequences compared here; and five of these (PFam65, 76, 78, 102 and 192) are likely explained by the missing lp25 sequence and terminal plasmid sequences in stain 297 (above), two (PFam72 and 175) are represented only by putative pseudogenes in B31, and PFam137 has its 297 member near the right end of the chromosome [51]. Thus representatives of only nine intact gene-containing families (PFam63, 68, 70, 71, 76, 88, 90, 193 and 194) appear to legitimately be missing from one or more of the four genomes, and these are largely the result of the failure of B31 to carry an lp28-5 plasmid and the large differences among the lp28-1s and lp36s of the different strains (Figures S2I and 8). Thus, JD1 carries no PFam63 gene (*revA*, normally on a cp32 plasmid in the other strains) and no PFam90 (on lp38 in the other strains) genes, and B31 has no PFam193 or 194 genes (on lp28-5 in other strains). These four PFams contain relatively large and so likely genuine genes; however, their roles have not been studied, except for PFam63 [102]. Thus, in spite of the plasmid content differences and numerous plasmid rearrangements discussed above, there are relatively few examples of PFams that are not present in all four strains, and the genome contents are in fact rather constant.

The number of paralogs present in individual PFams are, however, often variable (table S2); for example PFam01 contains restriction proteins and several related pseudogenes, and B31, 297, JD1 and N40 harbor at least 2, 2, 4 and 2 *apparently* intact members (297 and N40 might each have an additional intact member on their unsequenced lp25 and lp28-3, respectively). These proteins form two major sequence types (JD1_Y04/JD1_0905 and the other eight) that are about 18% different. In the larger group, B31_E02, B31_H09 (and very similar JD1_H09 and 297_H03), 297_Y09, JD1_E01, JD1_Y16, and N40_E01 proteins all differ from one another by 11±2%. It is not known if such sequence differences could cause restriction target specificity differences. The proteins B31_H09, JD1_H09 and 297_H03, which are encoded at identical positions on lp28-3s, are nearly identical; however, members within the two other syntenic groups B31_E02, JD1_E01 and N40_E01 on lp25 (Figure S2H) and JD1_Y16 and 297_Y09 on lp28-5 (Figures 6, S2H and S2J) differ by about 10%. These differences among syntenic orthologs suggest that there has been (presumably homologous) recombinational “shuffling” of sequences among the PFam01 paralogs.

Another family of note is PFam82, a putative IS605 type transposase [10], [187]. There are numerous PFam82 fragments in all four of the genomes compared here. In B31, 297 and JD1 no PFam82 member appears to be intact, but the N40 genome contains several *apparently* intact transposase genes (*n40_g05*, *n40_y12* and *n40_y15*). Of these, *n40_g05* is an ortholog of the frameshifted *b31_g05* gene, and *n40_y12* appears to have recently hopped into the partition gene cluster since the separation of the N40 lp28-5 from its common ancestor with JD1 and 297 (Figure 6).

#### Novel plasmid-encoded proteins

Only a few percent of the plasmid sequences of B31, 297, N40 and JD1 have no recognizable homologs in the other three strains, and as noted above only very few previously unknown *B. burgdorferi* plasmid gene types were identified in the three new sequences. This small number of “new” genes that encode previously unknown proteins are (i) *n40_y07* which encodes a DNA restriction methylase (above), (ii) homologs *n40_y02*, *jd1_y10* and *jd1_0909* that encode PFam194 putative lipoproteins, (iii) *n40_y06* with no predicted function, (iv) orthologs *297_j07* and *jd1_j07* that encode a putative lipoprotein of unknown function that have plasmid-borne homologs in the *B. afzelii* and *B. garinii* genomes (above), and (v) *jd1_z02* which encodes a putative PFam68 lipoprotein which has only apparently disrupted homologs in B31 and 297 and no homolog in N40.

### Plasmid DNA rearrangements

Clearly the patchwork of sequences with near identity to other sequences in the *B. burgdorferi* linear plasmids (Figures 2 and S2) indicates that there have been rearrangements sufficiently recently that there has been little time for extensive divergence of the sequences involved. Non-homologous rearrangements create novel sequence joints and thus novel juxtapositions of genes, and new combinations of such novel sequence joints can be created by homologous recombination among plasmid patches with similar sequence.

#### Non-homologous recombination

When mosaic sequences were analyzed *within* strain B31, we concluded that those rearrangements were most likely generated by non-homologous recombination [10], [38]. This conclusion is strongly reinforced by comparison of the plasmids in the four strains in this study. For example, near its right end JD1 lp28-3 has a novel sequence joint compared to B31 lp28-3. It joins sequences that are 99.6% identical to B31 lp28-3 and 99.5% identical to B31 lp36 (Figure 8). If it is assumed that the direction of this rearrangement is the formation of JD1 lp28-3 by a recombination between parents similar to these two B31 plasmids, the recombination point can be deduced to be at an exact bp in both putative parents, and there is no sequence similarity at all at this location in the putative parental plasmids (Figure S5A); also shown in Figures S5B–D are three other typical examples of such apparently non-homologous recombination events, an inversion in N40 cp32-5, the creation of a novel joint in N40 lp17 by recombination between B31 lp17-like and lp36-like plasmids, and the putative deletion that removed the putative *B31_a70* ortholog from JD1 lp54 (above). Thus, many of the rearrangements that gave rise to the organizational differences among the plasmids of the four strains appear to be the consequence of such non-homologous recombination.

#### Homologous recombination

**Figure 10:**
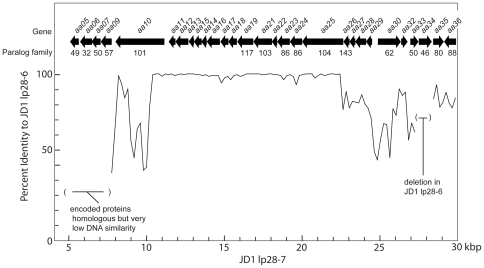
Comparison of JD1 and 297 lp28-6 plasmids. The DNAs of JD1 plasmids lp28-6 and lp28-7 were aligned with DNA strider [135], and the percent identity was computed for sequential 200 bp windows across the region shown in the figure. These two plasmids have two regions of near identity from about 8 to 9 Kbp and 10.5 to 22.5 Kbp, which abut regions with lower similarity. The JD1 lp28-7 open reading frames and their paralogous family relationships are shown above.

Homologous recombination could occur between any highly similar plasmid sequences, and the event that likely deleted the *n40_a70* gene from the N40 lp54 is shown in Figure S5E. But in most cases homologous recombination can only be recognized by reassortment of outside markers. The group of lp28-2, -6 and -7 linear plasmids shows a particularly clear example of such reassortment of outside markers. B31 lp28-2, N40 lp28-2 and JD1 lp28-6, have PFam101 *b31_g10* and cognate homologs that are locally syntenic and >98% identical, and plasmids 297 lp28-6 and JD1 lp28-7 have parallel PFam101 genes (*297_z06* and *jd1_aa10*) that are 99.8% identical. However, the first three genes contain nearly identical internal 1300 bp patches that are only ∼60% identical to the second two (which are also nearly identical). Figure 10 shows this relationship (within gene *jd1_aa10*) between JD1 lp28-6 and lp28-7. The most likely explanation for the existence of such abrupt changes in relatedness within similar sequences is recombination between two homologous but diverged sequences. Thus, the five parallel PFam101 genes are present as two “sequence types”, called A and B, in the five related plasmids (Figure S2L). Another example of such a relationship on these same plasmids is the 99.5% identical right end regions of JD1 lp28-6 and 297 lp28-6 (*jd1_z24*-*jd1_z28* and parallel 297 region) that are only about 75% identical to a homologous but divergent version on the other three plasmids (the two versions are called C and D in Figure S2L). N40 lp28-2, 297 lp28-6, JD1 lp28-6 and JD1 lp28-7 carry all four of the possible combinations of these sequence type alleles, AC, BD, AD, and BC, respectively. (Other, probably non-homologous, recombination events have occurred nearer to the left ends of these plasmids, but this does not impact these conclusions). It is extremely unlikely that localized mutational divergence can account for such patches of different but uniform relatedness, so two A alleles, for example, cannot be similar by virtue of separate but parallel divergence from a B ancestor. The presence of all four allele combinations in such a situation cannot arise through simple linear evolutionary descent [188], and at least one of them must have arisen by a recombination event between the other combinations. This event was almost certainly homologous recombination within the 12 Kbp of very highly similar (all ≥98.7% identical in pair wise comparisons) sequence between the A/B and C/D loci.

Homologous recombination has also occurred among the circular plasmids, and the cp32s are a robust example of such reassortment. Comparison of their sequences indicates that the most variable genes on the largely homologous cp32 plasmids lie in four regions as follows (see Figures S6A and S6B): region 1, *b31_s27*, *rev*, *mlp* and *bdr* genes [110], [189]–[195]; region 2, the partition genes including the PFam32 genes (*e. g.*, [75]); region 3, the *erp/elp/ospEF/p21* type surface protein genes [84]; and region 4, several alternative genes of unknown function immediately to the right of region 3 [145], [146], [196]. Each region's contents can be parsed into a small number of robust sequence types (alleles) by neighbor-joining tree analysis, and trees of each of the encoded homologous proteins are shown in Figure S6C–F. In most cases these sequence types are unambiguously very different from one another, for example the three Mlp protein types are more than 50% different from each other on branches with very high bootstrap support, as are the three PFam114 types (Figure S6C and F, and see figure 4 of Casjens *et al.* [75] for the PFam32 partition protein tree). Although it does not impact the conclusions drawn here, the evolution of the *erp* genes is oversimplified in this type of analysis, and will be discussed in more detail in a future publication (B. Stevenson, B. Jutras and S. Casjens, unpublished).

For each gene in the cp32 variable regions, the sequence types thus determined were assigned arbitrary numbers (see Figure S6C–F), and the findings are summarized in table 3, which lists the allele types present at each of the four variable regions on the thirty-two different cp32 sequences present in the four genomes (counting the fused “dimer” cp32 in JD1 as two such sequences arbitrarily divided as shown in Figure S2F). The sequence alleles present in these four regions of the cp32 plasmids have clearly not diversified only by linear evolutionary descent, and considerable reassortment of these different alleles is required to explain their current distribution. In fact, among these thirty-two cp32s, no two plasmids have the same constellation of variable region alleles. For example, the N40, JD1 and 297 cp32-12's each have the same region 2 PFam32 compatibility allele (by definition), but carry three different sets of alleles at each of the other three variable regions. On the other hand, although movement toward randomization has occurred, the alleles at these four variable positions appear not to have been shuffled to the point of complete loss of linkage in all cases. For example all four cp32-9s have the same alleles in region 3. In addition, many of the genes *within* the variable regions also appear to have been re-assorted, for example, *mlp* allele 2 is found in association with all four *bdr* alleles in region 1. Because the cp32 plasmids retain the same gene order and the vast majority of their genes have not been broken by non-homologous recombination events, this reassortment almost certainly occurred through numerous homologous recombination events.

**Table 3:** Homologous recombination among cp32 plasmids.

	Region 1^a^				Region 2^a^	Region 3^a^		Region 4^a^		
strain	S27	Rev	Bdr	Mlp	cp32	A	B	bapA	PF114	other
297	**–**	**–**	**1**	**1**	1	**4**	**1**	**–**	**1**	**–**
297	**1**	**–**	**3**	**2**	3	**2**	**–**	**–**	**3**	**–**
297	**–**	**1**	**–**	**1+2**	4	**3**	**–**	**–**	**–**	**1**
297	**–**	**–**	**3**	**2**	5	**4**	**1**	**–**	**1**	**–**
297	**–**	**–**	**3**	**1**	6	**2**	**–**	**1**	**–**	**4**
297	**–**	**–**	**1**	**2**	7	**3**	**–**	**–**	**–**	**5**
297	**–**	**–**	**2**	**1**	9	**4**	**1**	**–**	**–**	**–**
297	**–**	**–**	**1**	**1**	11	**2**	**–**	**–**	**–**	**3**
297	**–**	**1**	**–**	**2**	12	**4**	**1**	**–**	**1**	**–**
JD1	**1**	**–**	**1**	**2**	1	**2**	**–**	**–**	**–**	**3**
JD1	**–**	**–**	**3**	**2**	3	**2**	**–**	**–**	**3**	**–**
JD1	**–**	**–**	**1**	**2**	5	**1**	**–**	**–**	**1**	**–**
JD1	**–**	**–**	**3**	**2**	6	**4**	**2**	**–**	**3**	**–**
JD1	**–**	**–**	**3**	**2**	8	**4**	**1**	**–**	**3**	**–**
JD1	**–**	**–**	**2**	**2**	9	**4**	**1**	**–**	**3**	**–**
JD1	**–**	**–**	**3**	**2**	10	**2**	**–**	**1**	**–**	**4**
JD1	**–**	**–**	**1**	**1**	11	**2**	**–**	**–**	**3**	**–**
JD1	**–**	**–**	**1**	**2**	12	**3**	**–**	**–**	**–**	**1**
B31	**–**	**1**	**–**	**2**	1	**4**	**1**	**–**	**1**	**–**
B31	**1**	**–**	**1**	**2**	3	**2**	**–**	**1**	**–**	**–**
B31	**–**	**–**	**4**	**2**	4	**4**	**2**	**–**	**3**	**–**
B31	**–**	**1**	**–**	**2**	6	**2**	**–**	**–**	**–**	**2**
B31	**–**	**–**	**1**	**2**	7	**2**	**1**	**–**	**3**	**–**
B31	**–**	**–**	**1**	**2**	8	**4**	**1**	**–**	**1**	**–**
B31	**–**	**–**	**2**	**2**	9	**4**	**1**	**–**	**2**	**–**
B31	**–**	**–**	**3**	**1**	10	**1**	**–**	**–**	**1**	**–**
N40	**–**	**–**	**1**	**1**	4	**2**	**1**	**–**	**–**	**–**
N40	**–**	**–**	**1**	**2**	5	**2**	**–**	**–**	**–**	**2**
N40	**–**	**–**	**1**	**2**	7	**4**	**2**	**–**	**3**	**–**
N40	**–**	**–**	**4**	**1**	9	**4**	**1**	**–**	**1**	**–**
N40	**–**	**1**	**–**	**1**	10	**1**	**–**	**–**	**3**	**–**
N40	**–**	**–**	**–**	**–**	12	**2**	**–**	**–**	**–**	**–**

a ClustalX2 0.3 [214] was used to create a neighbor-joining tree for proteins encoded in the genes the four cp32 “variable regions”. The variable regions were identified by inspection of matrix comparison plots (Figure S6A), and their positions are shown in Figure S6B. Robust branches (amino acid “sequence types”) were identified for the proteins encoded by the genes in each region, and these different protein types were given arbitrary numbers. The trees and definitions of sequence types are shown in figures S6C–F. Where a region 3 has two genes, the left (transcriptionally upstream) gene is in column “a” and the right gene is in column “b”. A dash “–” indicates no gene is present; PF, paralogous family. The genes in the rightmost region 4 “other” position are as follows: 1, *mlp* type 3 gene; 2, homolog of *b31_m39*; 3, homologs of *297_w43* (very weak similarity to *b31_m39*) and *297_w44* (PFam55); 4, homolog of *297_m41*; 5, unique *297_o29*. The different B31_S27, Rev and BapA proteins are all so similar that subtypes were not evident.

### Mutational decay and pseudogenes

The constant regions of the *Borrelia* chromosomes have very few obvious pseudogenes (unlike the plasmids and plasmid-like right end chromosomal extensions). However, our previous analysis of the B31 plasmids identified over 150 pseudogenes as regions of nucleotide sequence similarity to intact genes but whose reading frame is disrupted and/or truncated [10]. A large majority of these pseudogenes reside on the “rapidly evolving”/organizationally variable subset of the linear plasmids (probably all linear plasmids except lp54), most of which also carry extensive regions that contain no long open reading frames. The latter appear to represent decaying, useless DNA [38]. Many of these B31 non-coding regions are present in extremely similar orthologous sequences of the plasmids of the other three strains. A typical example of this resides on the lp28-4 plasmids. Here the three largest tracts of apparently non-coding DNA (including >6 Kbp that includes one recognizable ∼650 bp degenerate pseudogene *b31_i08.1*) are all present in all four strains and more than 99% identical to one another (Figure S7). Here and elsewhere, the B31, N40, JD1 and 297 linear plasmids carry many of the same pseudogenes that have changed little since their last common ancestor. In addition, the linear plasmid sequences present in N40, JD1 and 297 that have no clear orthologs in B31 have similar densities of apparent pseudogenes; for example, Figure 9 shows that the regions of 297 lp38 that have no B31 orthology harbor a number of pseudogenes (*e. g.*, *297_j08-j14*) which are related to, but not orthologous to other known *Borrelia* genes. In a second example, the JD1 and 297 lp28-5s, which are not present in B31, carry substantial regions of apparently noncoding DNA, which include apparently truncated genes (*e. g.*, *jd1_y13* and *297_y05*) and regions that have no convincing open reading frames (*e. g.*, ∼1.5 Kbp between *jd1_y05* and *jd1_y08* and orthologous DNA in 297; Figure 6).

Mutational inactivation of plasmid genes is apparently ongoing, since among the strains studied here there are a number of plasmid orthologs in which only a subset is disrupted. Some examples are as follows:

#### (i) Mutations in the JD1 lineage

The putative lipoprotein-encoding gene *jd1_h20* in plasmid lp28-3 has a frame-breaking insertion of a single T at 12731 relative to its orthologs in B31 (*b31_h18*) and 297 (*297_h12*); these three orthologs are >99% identical. Also in JD1, the homologs of *b31_i26* and *b31_i34* have an in-frame stop codon and frameshift, respectively, compared to the orthologous regions of the other three lp28-4s (Figure S7).

#### (ii) Mutations in the B31 lineage

The lp28-2 genes *b31_g03* (PFam48) and *b31_g05* (PFam82; putative transposase) were originally suggested to be pseudogenes that contain single frameshifting mutations on the basis of comparison with paralogs in strain B31 and/or genes in other bacteria [10], [38]. The reading frames of orthologs of both of these genes (*n40_g03* and *n40_g05*) appear to be intact in N40, confirming this interpretation. The nucleotide changes that shifted the frames in these two genes in B31 are a tandem duplication of a TGGAG (N40 bp 2327-2331) and run of 4 T's in N40 is lengthened to 5 T's in B31 (B31 bp 3675), respectively.

#### (iii) Mutations in the N40 lineage

The lp28-2 genes *n40_g17* and *n40_g22* have frameshifting mutations relative to their B31 orthologs (Figure S2L). The fact that paralogous sequence on JD1 lp28-6, 297 lp28-6, and parallel *B. garinii* PBi (accession No. AY722917), and *B. bissettii* DN127 (accession No. CP002760) plasmids carry the longer form of both genes strongly suggests that these N40 genes are inactivated by mutation. Here a run of 8 T's in B31 (B31 bp 15923-30) is shortened to 7 in N40 and a run of 4 C's in B31 (B31 bp 20677-80) is lengthened to 5 in N40, respectively.

#### (iv) Mutations in the JD1 and 297 lineages

JD1 and 297 carry a version of lp17 that has sequences similar to B31 lp36 at one end (above). This region carries *adeC* (*b31_k17*) and *b31_k15* homologs that contain frameshift mutations relative to the lp36 homologs that are present in all four genomes. The JD1 and 297 lp17 *adeC* genes (*jd1_d02* and *297_d01*) are 99.3% identical to one another, and their sets of disrupting mutations are overlapping but not identical, suggesting that their decay began before the separation of the lp17 plasmids in the JD1 and 297 lineages and has continued since than time. In these lp17 *adeC* pseudogenes, a run of six A's is extended to seven at position 1111 of JD1 lp17, a run of nine T's in the intact genes is shortened to eight in both genes (at 2163 in JD1), and a run of two C's in the intact genes is three and four long in 297 and JD1, respectively (at 3049 in JD1). Many of the recent, frame-disrupting changes in plasmid genes appear to be slippage mutations where runs of a single nucleotide are shortened or lengthened.

### Tandem direct repeat sequences

Since they can expand and contract relatively rapidly on an evolutionary scale, the number of repeats in “variable number tandem repeat” (VNTR) tracts is often used for separation and identification of closely related lineages of any organism, and bacteria are no exception [197]. Such methods have been applied to other *Borrelia* species [198], [199] and such repeat tracts have been previously noted on lp21 (63 bp repeat) [10], lp38 (17 bp repeat) [158], [159] and lp28-4 (27 bp repeat in *vraA* gene) [138]. In addition, members of the *bdr* gene family (PFam80) contain more complex imperfect direct repeats [191], [200]). Although Zuckert and Barbour [192] did not observe changes in the number of repeats in the *bdrT* (*b31_g33* on lp28-2) gene they examined during growth in culture, we find, for example, that the *bdrT* gene contains about 9 repeats in B31 and its otherwise 99.4% identical ortholog in N40 has about 6 repeats (imprecision is due to the presence of partial and overlapping repeats). So strains from different rRNA [57]–[60] or OspC [62]–[65] lineages can have different numbers of *bdr* gene repeats.

Tandem Repeats Finder ([201] and http://tandem.bu.edu/trf/trf.html) was used to identify tandem repeats of short sequences in B31, N40, JD1 and 297, and table S3 lists twelve such tracts that show substantial variation among these four strains. These include repeat tracts within three chromosomal genes, *b31_0210*, *b31_0546* and *b31_0801* and their orthologs in the other strains, as well as tracts on lp17, lp28-4 and lp54, all of which are present in essentially all *B. burgdorferi* strains tested and so could be useful in tracking closely related members of this species. Of these chromosomal genes, *b31_0210* (*lmp1*) is required for persistence in murine tissues [202], and *b31_0801* is predicted to encode a translational initiation factor. The B31 *vraA* (*b31_i16*) lp28-4 gene encodes a surface lipoprotein [138] that contains 21 perfect repeats of the highly charged nine amino acid sequence KKKQQEEEL; the *vraA* gene is a member of PFam60, but the other members of this family do not contain this nine amino acid sequence. The N40, JD1 and 297 *vraA* orthologs have 31, 24 and 35 repeats, respectively. These strains belong to different rRNA and OspC lineages of *B. burgdorferi*; however, the *vraA* genes in two other strains from the B31 rRNA lineage 1 (ZS7 and Bol26) and one from its sister rRNA lineage 3 (strain 64a) contain 3, 1 and 11 repeats, respectively [44], showing that the length of at least this VNTR can apparently change quite rapidly.

Measurement of precise lengths of very long VNTRs is not simple, so they are likely to be less useful in lineage tracking, but strain B31 plasmid lp21, the homologous chromosome region in strain 297, and JD1 lp38 carry about 11, 11 and 8 Kbp of imperfect 63 bp tandem repeats [10], [51]. The lp28-5 plasmids carry between 5 and 26 imperfect repeats of an unrelated 133 bp sequence. All six translational reading frames are blocked in each of the 133 bp repeats. Since accurate assembly of sequencing runs in such regions is difficult, we experimentally confirmed the approximate length of the block of tandem 133 bp direct repeats to about 1.8 Kbp in N40 lp28-5 by measuring the size of DNA restriction fragments that contain mostly the repeat region (data not shown). The roles of such long repeat tracts are unknown, and they are quite unusual in prokaryote genomes.

### Conclusions

#### Overall genome relationships

The *B. burgdorferi* genome contains an approximately 903 Kbp chromosome “constant region” and plasmids cp26 and lp54, which are quite evolutionarily stable. Comparisons among the four genome sequences analyzed in this study show that these three replicons are nearly completely syntenic and more than 98% identical in nucleotide sequence among strains from different rRNA/OspC lineages. In addition to these highly conserved regions, the genomes of this species also contain a large number of much more variable plasmids, and the majority of *B. burgdorferi* isolates carry 7–20 Kbp of variable, plasmid-like sequences at the right end of the otherwise genetically stable chromosome. Much of this more variable portion of the genome is also very highly related in sequence among strains, but it has suffered numerous rearrangements. For example, the orthologous parts of variable plasmids lp28-3, 1p28-4 and lp36 (which constitute the majority of the sequence of each of these plasmids) are each ≥99% identical among the cognate plasmids in the four strains. In most of the rearrangements found in the plasmids, non-homologous DNA has apparently replaced previously existing sequences. The very high identity (>99%) of the sequences in two or more related but rearranged plasmid versions indicates that these rearrangements happened rather recently on an evolutionary time scale.

Some of the variable regions appear to have suffered multiple sequential or parallel replacements by different non-homologous plasmid sequences. For example, (i) three lp17s have three different left ends but have the same right end; (ii) three lp28-4s have two different right ends and three different left ends; (iii) the three chromosomes with right end extensions have three largely non-homologous extensions; and (iv) the *arp* gene and *vls* cassette region each lie on several different plasmid types in the different genomes. These and other observations strongly indicate that the linear plasmid rearrangement process is ongoing, and that such events have happened independently in different *B. burgdorferi* lineages. Yet, in spite of the many organizational and plasmid content differences, the gene content of the four strains remains relatively constant. Although there is some variation in the number of members of the different paralogous gene families each strain carries, a large majority of such families are represented in all four strains. In addition, only a few “new” previously unknown *B. burgdorferi* gene types were identified in the three new genome sequences.

#### Rates of horizontal exchange?

The presence of such a large number of plasmids, some of which appear to be prophages, suggests that horizontal exchange of these DNAs could be frequent [10], [40], [144], [203]. Indeed, previous analysis of several plasmid genes has suggested that there has been “extensive” horizontal exchange among *B. burgdorferi* lineages (*e. g.*, [66], [79], [84], [158], [204]–[206]), and Eggers and Samuels [39] have demonstrated that in the laboratory cp32 plasmids can transfer between strains as phage virions.

Among of the forty sequenced linear plasmids in the four strains, there are only four pairs of cognate linear plasmids in which we found no organizational differences between strains. These are lp17, lp28-1 and lp36 in 297 and JD1, and lp54 in 297 and N40 (noted by arrows in Figure 2; because of undetermined sequence at the ends of 297 lp28-3 and lp28-4, it is not known whether they might be organizationally the same as their B31 or JD1 and N40 or JD1 cognates, respectively). In addition, the cognate plasmid pairs of lp28-5, lp28-6 and lp38 are more similar to each other in JD1 and 297 than to the cognate plasmids in the other two strains. Although there are some substantial differences between the JD1 and 297 plasmids, these two linear plasmid sets appear to be more like one another than the other pair wise combinations. Although the JD1 and 297 rRNA IGS/chromosomal MLST/OspC lineages do not appear to be particularly closely related [57], [58], [67], the similarity of their plasmid contents might indicate that they are in fact more closely related than previously suspected, and the very different plasmids that are present in JD1 and 297 (*e. g.*, their lp38s) could be examples of horizontal transfer of plasmids, but study of more isolates will be required to determine if this is true.

Previous work and the sequences analyzed here show that the cp32 plasmids in different rRNA/OspC *B. burgdorferi* lineages are similar in overall structure, but can have considerable differences at the four variable positions discussed above. On the other hand B. Stevenson and co-workers (personal communication) have shown, by sequencing several of the variable regions, that strains B31 and BL206 (both rRNA IGS lineage 1, OspC type A [58], [62]) appear have very similar cp32 complements, as do strains 297 and Sh-2-82 which are both rRNA lineage 2, OspC type K (Sh-2-82 our unpublished results). Thus, although the complete BL206 and Sh-2-82 genome sequences have not been determined, it appears that different members of the same rRNA/OspC lineages can have highly similar cp32 contents, implying that transfer between these lineages may not be so rapid in the wild that plasmid contents are randomized. None of the completely sequenced cp32 sets present presented here (from members of four different rRNA lineages) are as highly related as the above strain pairs (table 3), but they will provide a robust basis for the future determination of whether the cp32 contents of all or most isolates within rRNA/OspC lineages are indeed similar. This is especially intriguing because the cp32 plasmid prophages could be prone to particularly rapid horizontal transfer [10], [40], [144], [203].

#### Plasmid types

Have all extant *B. burgdorferi* sensu stricto plasmid “compatibility types” been identified? In addition to the 26 PFam32 types mentioned above that are present in the four strains, a 27^th^ PFam32 type has been reported for “cp32-13” in California isolate CA15 ([84] and our unpublished results for other strains). In addition, strain B31 linear plasmid lp28-1 carries two apparently intact PFam32 genes. One of them, *b31_f13*, represents a novel PFam32 type that lies in a partition gene cluster that is missing its PFam57/62 member gene and so was ignored in previous analyses. It seems quite possible that plasmids of this compatibility type (a 28^th^ type) will be found in other strains. If PFam32 types are distributed randomly in natural isolates, saturation has probably not been reached by analyzing only four strains, although the number of undiscovered compatibility types is likely not large since the N40, JD1 and 297 plasmid sequences add only five “new” types (lp28-5, lp28-6, lp28-7, cp32-11 and cp32-12) to the 21 types previously known in strain B31. We note that the four sequenced isolates are all from a geographically rather restricted region (southern New England and New York), and it will be interesting to determine whether *B. burgdorferi* isolates from other locations have different plasmid types.

The organizational variation *within* each PFam32 type in the four strains studied here suggests that the overall number of *B. burgdorferi* linear plasmid “organizational types” may not be small. Nonetheless, the facts that (i) some pairs of organizationally identical cognate plasmids exist among these four strains, and that (ii) there are numerous novel sequence joints that are present in more than one strain, suggest that a limited number of such variants exists for each plasmid compatibility type in nature. Our analyses also indicate that a many of the rearrangements that formed the different organizational types occurred so recently that the sequences involved have diverged substantially less than one percent since the rearrangement, yet the process is not so fast that every *B. burgdorferi* isolate has a completely different set of plasmid organizations. Since no such rearrangement has been observed in the laboratory, such events are in fact quite rare, at least under laboratory conditions.

#### Future directions

Many important unanswered questions regarding Lyme *Borrelia* genomics and population structure remain, including the following: Do other *B. burgdorferi* isolates harbor additional plasmid compatibility types? How many organizational subtypes within each plasmid compatibility type exist in *B. burgdorferi* in nature? Are any plasmids or plasmid subtypes restricted to particular *B. burgdorferi* chromosomal lineages or geographic areas? What are the relationships among plasmids of different *B. burgdorferi* sensu lato species? Are plasmids transferred between strains as whole entities or as fragments, and what are the rates of transfer in nature? Are plasmids transferred only within species or between closely related *Borrelia* species in nature? What gene set constitutes the *B. burgdorferi* pangenome? We have recently sequenced nine additional *B. burgdorferi* sensu stricto genomes [44] and eight genomes of related species [45], [46], [49] in order to begin to extend our knowledge in all of these areas.

## Materials and Methods

### Strains and DNA preparation

Low passage cultures of *B. burgdorferi* isolates B31, N40, JD1 and 297 were the kind gifts of Drs. Alan Barbour, Martin Schriefer, Tom Schwan and Justin Radolf, respectively. In this study, low passage cultures of N40, JD1 and 297 were propagated in complete BSK-II medium (Sigma, St. Louis, MO) at 34°C without isolation through a single colony or passage through a mouse in order to minimize loss of plasmids. For isolation of whole genomic DNA, 1 liter of log-phase bacteria (∼4×10^7^ bacteria/ml) were harvested by centrifugation at 10,000 rpm for 30 min at 4°C. The bacterial pellet was washed twice with 10 mM Tris pH 7.5, 100 mM NaCl buffer, and resuspended in 430 µl TES (10 mM Tris pH 7.5, 100 mM NaCl, 10 mM EDTA). Subsequently, 10 µl of freshly prepared lysozyme (50 mg/ml), 50 µl Sarkosyl (10%), and 10 µl proteinase K (10 mg/ml; Sigma, St. Louis, MO) were added, and the mixture was incubated at 50°C overnight prior to RNase treatment. DNA was then extracted with phenol/chloroform and chloroform, precipitated with ethanol, and finally resuspended in TE buffer (1 mM Tris pH 7.5, 1 mM EDTA). Strain 297 plasmids were isolated with a Qiagen (Valencia, CA) Plasmid Midi-100 Kit according to the manufacturer's recommendations.

### Sequencing and sequence analysis

#### Sequencing, assembly, and gap closure

Sanger shotgun sequencing and assembly were performed as described previously for genomes sequenced at TIGR/JCVI [207]. All three genomes were sequenced to closure. Briefly, small-insert and medium-insert plasmid libraries were generated by random nebulization and cloning of genomic DNA. The following libraries were generated: N40, one 3–4 kb small-insert and one 6–8 kb medium-insert libraries; JD1, one 2–3 kb small-insert, one 3–4 kb small-insert and one 8–12 kb medium-insert libraries; 297, one 3–4 kb small-insert and one 10–12 kb medium-insert libraries. In the random sequencing phase, at least a 9-fold coverage across the genome was achieved from the shotgun sequencing libraries generated for each strain. More specifically, a total of 19971, 59714 and 11126 Sanger sequencing reads were generated during the random sequencing phase for N40, JD1 and 297 respectively. The sequences were assembled using the TIGR Assembler (www.jcvi.org/cms/research/software/) and the Celera Assembler (http://sourceforge.net/projects/wgs-assembler), and the scaffolds constructed using TIGR BAMBUS [208]. All sequence and physical gaps were closed by editing the ends of sequence traces, primer walking or transposon-primed sequencing on plasmid clones, and combinatorial PCR followed by sequencing of the PCR product.

A number of the termini of bulk-determined sequence contigs were extended by sequencing DNAs from inverse PCR or by direct PCR amplification using outside primers designed from sequence predicted to be orthologous by comparison with plasmids from one of the other strains. About 8, 19, and 5 Kbp were determined by these directed methods in the N40, JD1 and 297 plasmid sequences. The lengths of the sequence contigs and plasmid sizes (determined by pulsed-field electrophoresis and Southern analysis as in Casjens *et al.* [17]), as well as lengths of the “missing” unsequenced terminal regions (calculated from the sizes of terminal restriction fragments) are given in Table S1. The linear 297 plasmid sequence contigs are often missing from 2000 to 2500 terminal bp; this was a poorly understood property of the DNA libraries, not sequencing depth. The GenBank accession numbers for the sequences determined in this study were reported in Schutzer *et al.* [44] and are included in Table S1.

#### Sequence annotation

For the N40, 297 and JD1 genomes, an initial set of ORFs likely to encode proteins was identified by GLIMMER (http://cbcb.umd.edu/software/glimmer/). This first set of open reading frames (ORFs) was then manually curated so that ORFs equal to or less than 50 codons long (not counting the stop codon) were removed unless they are homologs of a similarly sized gene of known function, and ORFs in the 51–100 codon range were only included if their reading frame is intact in cognate sequence in all of the strains that carry the sequence. ORFs that overlapped were inspected visually and, in some cases, removed. ORFs were searched against a nonredundant protein database as described previously for all TIGR genomes. Frameshifts and point mutations were detected, checked and corrected where appropriate. Remaining frameshifts and point mutations are considered authentic, and corresponding regions were annotated as “authentic frameshift” or “authentic point mutation,” respectively. Two sets of hidden Markov models (HMMs) were used to determine ORF membership in families and superfamilies. These included 10,340 HMMs from PFAM version 23.0 (http://pfam.sanger.ac.uk/) and 3,603 HMMs from TIGRFam version 8.0 (www.jcvi.org/cms/research/projects/tigrfams/overview/). TOPPRED [209] was used to identify membrane-spanning domains in proteins. For ease of comparison, the genome of strain B31 was also re-annotated by this pipeline, and this reannotation can be found in the original accession numbers of the B31 replicons (Table S1 and [10], [11]).

Some of the plasmids carry full-length, degenerate pseudogene paralogs of other intact plasmid genes. These were not annotated (as they were in the original B31 annotation). The automated ORF searches identified some smaller ORFs within these pseudogenes, and since they could theoretically be expressed they were kept in the predicted ORF list. Translation frameshift and in-frame stop differences among the strains sequenced here were compared to homologs in *B. garinii* PBi [47], *B. afzelii* PKo ([48] and our unpublished results) and *B. bissettii* DN127 (our unpublished results) to determine which state is most likely functional.

#### Open reading frame nomenclature

*Borrelia* researchers have usually used the “locus tags” of the strain B31 genome GenBank annotation [10] as names for genes and their encoded proteins. Thus, according to bacterial convention, the B31 chromosomal genes have often been named “*bb0xxx*” (lower case and italicized) in ascending order from *bb0001* upward across the chromosome. The B31 plasmid locus tag names are similar but have the form “*bb$xx*” in which “*$*” is a letter code denoting which plasmid type carries the gene (*e. g*., *bba74* encodes protein B31_A74 and lies on lp54, *bbs09* lies on cp32-3, *etc.*). Increased genome sequencing forces the use of more complex locus tags such as, Bbu*jd1_*Axx for strain JD1 plasmid lp54. To avoid very long gene names now that multiple genomes have been sequenced, we suggest the use the form “strain name_locus tag number only” for gene names so that their strain source is included (*e. g*., *b31_0843* for a B31 chromosomal gene, and “*jd1_a34*” for JD1 plasmid lp54 reading frame 34 with plasmid letter code lower case “a”), and we follow these conventions here. Table S4 lists the locus tag letters with their corresponding plasmids for all the plasmids in the four current genome sequences as well as for our additional unpublished sequences. In the different genomes, the same locus tag numbers in the B31, N40, 297 and JD1 chromosome, cp26 and lp54 indicate orthology of the corresponding genes; however, organizational differences in the other plasmids made this system unworkable, so the same locus tag numbers on these replicons do *not* indicate orthology.

#### Methods of ortholog/paralog analysis

We identified orthologous plasmids by inspection and by using NUCMER [210] and BLASTn [211]. For each set of orthologous replicons, we identified orthologous ORF sets by first finding all homologs of each ORF using all-against-all BLASTn [211]. Homologous ORFs were clustered using the MCL algorithm [212]. Within each homolog cluster, orthologs were distinguished from paralogs by visual inspection of gene orders displayed by the authors' unpublished synteny browser and by matrix comparison with DNA Strider [135]. Percent identity of DNA and protein sequences was calculated by DNA Strider using alignments created by that program. Protein multiple sequence alignments were constructed using ClustalW 1.83 [213] and ClustalX2 0.3 [214]. Codon alignments were derived from protein alignment templates using PERL scripts.

## Supporting Information

Figure S1 **The right end of the B. Burgdorferi JD1 chromosome.**
(PDF)

Figure S2 **Open reading frame maps for plasmids carried by** ***B. burgdorferi*** **strains B31, N40, JD1 and 297.**
(PDF)

Figure S3 **Vls cassette regions.**
(PDF)

Figure S4 **Ip28-6 copy number.**
(PDF)

Figure S5 **Recombination points.**
(PDF)

Figure S6 **Neighbor-joining trees of proteins encoded by** ***B. burgdorferi*** **cp32 variable regions.**
(PDF)

Figure S7 **Orthologous non-coding DNA in Ip28-4.**
(PDF)

Table S1 **B31, JD1, N40 and 297 replicon sizes and accession numbers.**
(PDF)

Table S2 **Paralogous protein families in four** ***B. burgdorferi*** **genomes.**
(PDF)

Table S3 **Major tandem repeat tracts.**
(PDF)

Table S4 **Lyme agent** ***Borrelia*** **plasmid letter appellations for locus tags.**
(PDF)
