# Supplementary material for: Genome Stability of Lyme Disease Spirochetes: Comparative Genomics of Borrelia burgdorferi Plasmids
Source: PLoS One. 2012 Mar 14;7(3):e33280. doi: 10.1371/journal.pone.0033280 (PMC3303823; doi:10.1371/journal.pone.0033280)
Supplement: Figure S1 — The right end of the B. Burgdorferi JD1 chromosome. (PDF) [file pone.0033280.s001.pdf]

Figure S1. The Right End of the *B. burgdorferi* JD1 Chromosome

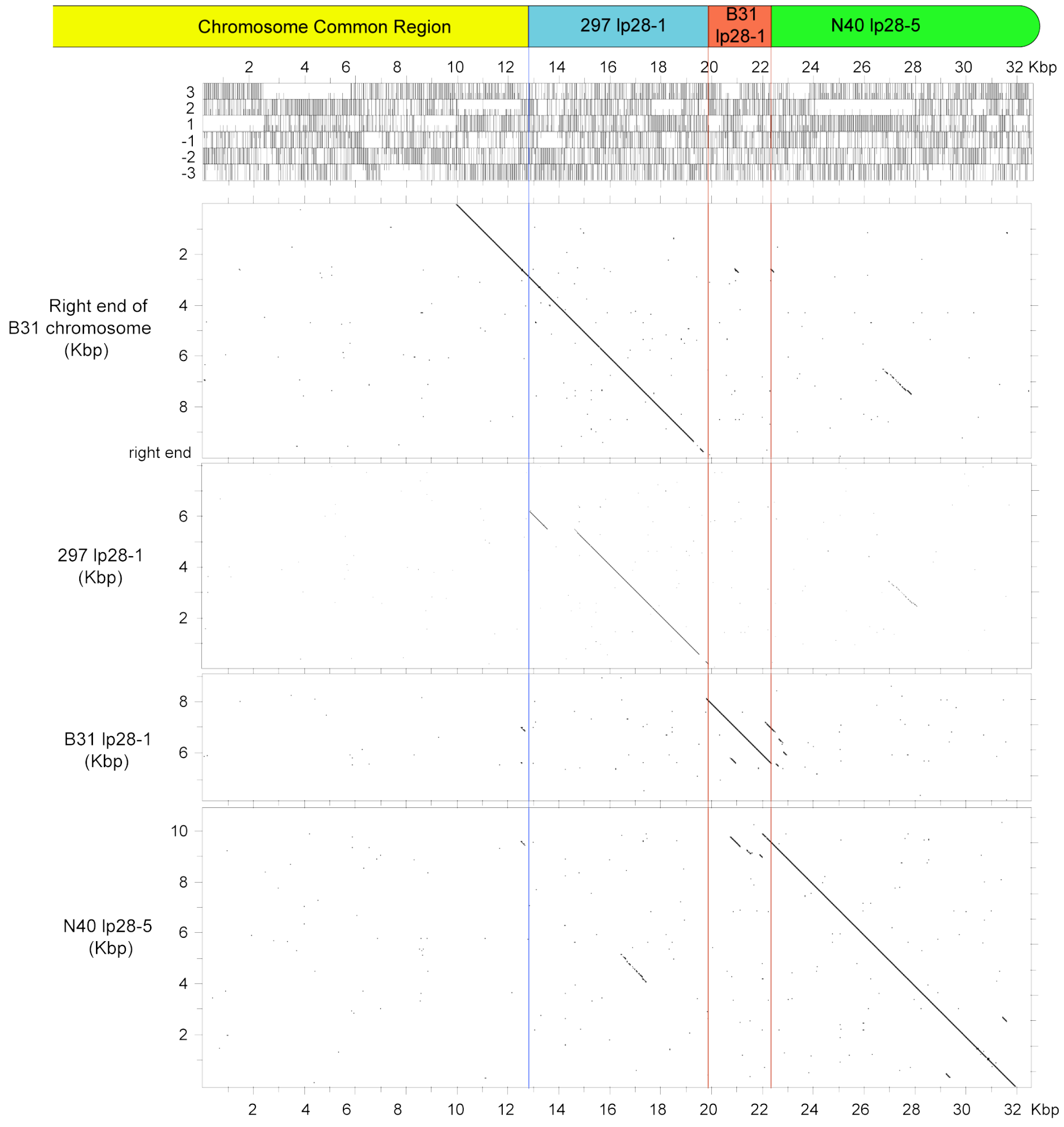

Figure S1. The *B. burgdorferi* JD1 chromosome right end extension. Matrix plots comparing the right end of the JD1 chromosome with plasmids in other strains are shown (plots with 19 identities in a 23 bp scanning window were generated by DNA Strider [1]). An open reading frame plot is shown above, where all six reading frames are indicated (1, 2 and 3 are left to right and -1, -2 and -3 are right to left) with stop codon locations marked by vertical lines across the frame, and potential ATG start codons marked by vertical lines half way through the frame.

Reference

[1] Douglas SE (1994) DNA Strider. A Macintosh program for handling protein and nucleic acid sequences. Methods Mol Biol 25: 181-194.
