# Supplementary material for: Genome Stability of Lyme Disease Spirochetes: Comparative Genomics of Borrelia burgdorferi Plasmids
Source: PLoS One. 2012 Mar 14;7(3):e33280. doi: 10.1371/journal.pone.0033280 (PMC3303823; doi:10.1371/journal.pone.0033280)

Figure S2. Open reading frame maps for plasmids carried by *B. burgdorferi* strains B31, N40, JD1 and 297.

Open reading frames (ORFs) that are considered to be potential functional genes were annotated as described in Methods of accompanying report. Above, names or predicted functions of genes of current interest are noted in red.

Yellow shading indicates highly similar (>85% nucleotide sequence identity) regions, and red text in these regions indicates selected identity values. Blue shading and associated text indicate regions that are different among the plasmids from the four strains and are very similar to sections of other non-cognate plasmids. Asterisks (*) denote plasmids present in other *B. burgdorferi* draft genome sequences and in one case (N40 lp28-4 left end) sequence at the end of B31 plasmid lp28-1 that was not determined in the original genome sequence (accession # FJ472338). The right end of the bp scale for each plasmid gives the length of the plasmids (this value and the bp scales *do not* include the B31 terminal sequences determined by Tourand *et al.* [1]; these values in general extend each end less than 100 bp, except the left end of B31 lp28-1 in which 767 bp were missing from the originally determined sequence [2]).

Putative pseudogenes are marked with "X"s and are defined as (i) regions of nucleotide sequence similarity to apparently intact genes but whose reading frame is disrupted and/or truncated relative to those intact genes or (ii) small ORFs which lie in a larger pseudogene [5]. It is not possible to know at this time whether or not some of these "pseudogenes" might actually be expressed and have a function.

Maps of lp5, lp21 and lp56 are not shown because they have no cognates in N40, JD1 or 297. The maps of circular plasmids are linearized for displayed at arbitrary points, which match those previously used by Casjens *et al.* [3] where possible.

S2A. Key for color of ORFs in maps.

ORF colors in figure S2 maps denote known or predicted functions.

(†) Includes genes that encode DNA restriction/methylation enzymes, transposase and plasmid partition proteins

S2B. cp9 maps

S2C. cp26 maps

S2D. B31 cp32 maps

Slanting black line on cp32-10 indicates the location at which it is integrated into plasmid lp56.

S2E. N40 cp32 maps

S2F. JD1 cp32 maps. Plasmid cp32-1+5 consists of two fused cp32-type plasmids that are shown in the first two lines of the figure.

S2G. 297 cp32 maps

S2H. lp25 maps

S2I. lp28-1 maps

S2J. lp28-3 maps

S2K. lp28-4 maps

S2L. lp28-2, lp28-6 and lp28-7 maps. Green letters represent sequence types of *b31_g10* and *b31_g29* and homologues as discussed in text.

S2M. lp54 maps

References

1. Tourand Y, Deneke J, Moriarty TJ, Chaconas G (2009) Characterization and in vitro reaction properties of 19 unique hairpin telomeres from the linear plasmids of the lyme disease spirochete. J Biol Chem 284: 7264-7272.
2. Fraser CM, Casjens S, Huang WM, Sutton GG, Clayton R, *et al.* (1997) Genomic sequence of a Lyme disease spirochaete, *Borrelia burgdorferi*. Nature 390: 580-586.
3. Casjens S, Palmer N, van Vugt R, Huang WM, Stevenson B, *et al.* (2000) A bacterial genome in flux: the twelve linear and nine circular extrachromosomal DNAs in an infectious isolate of the Lyme disease spirochete *Borrelia burgdorferi*. Mol Microbiol 35: 490-516.

Figure S2A. Color key for figure S2 ORFs

- Nucleic acid and nucleotide metabolism†
- Outer surface protein
- Transport
- Possible phage virion assembly
- mlp family
- rev family
- erp/elp/ospEF
- Pseudogenes not analyzed in detail in this study (see Casjens et al., 2000)
- Antigenic
- Complement factor interaction
- bdr family
- Holin/hemolysin
- bapA
- vlsE & cassettes
- Unknown function

Figure S2B. cp9

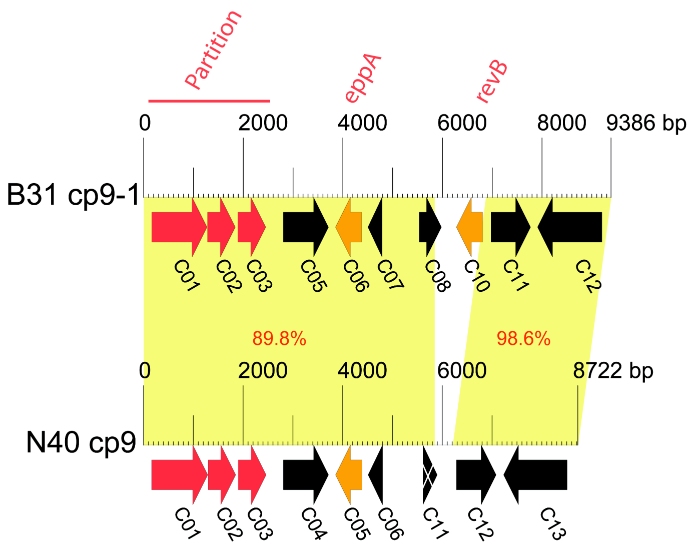

Figure S2C. cp26

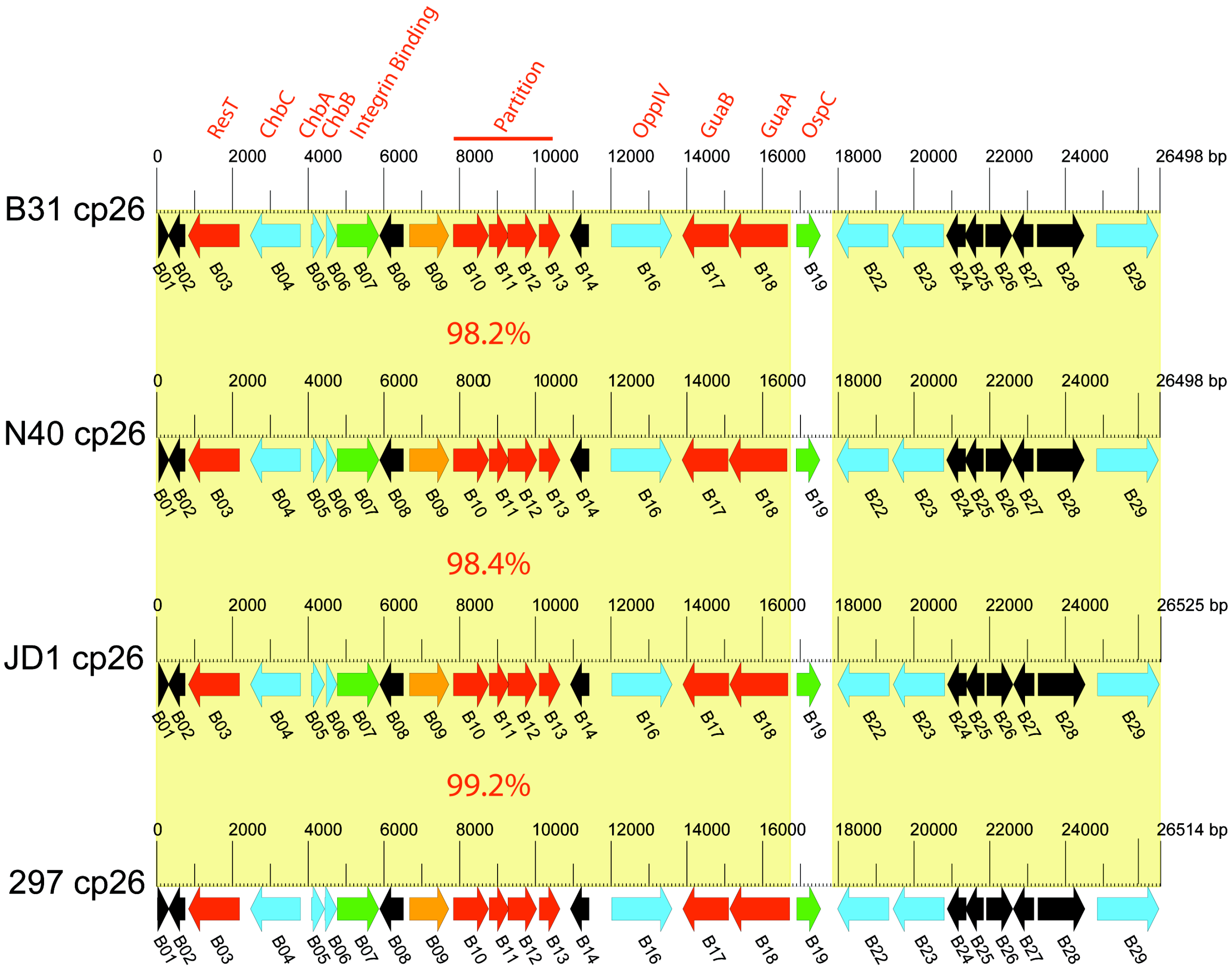

Figure S2D. B31 cp32s

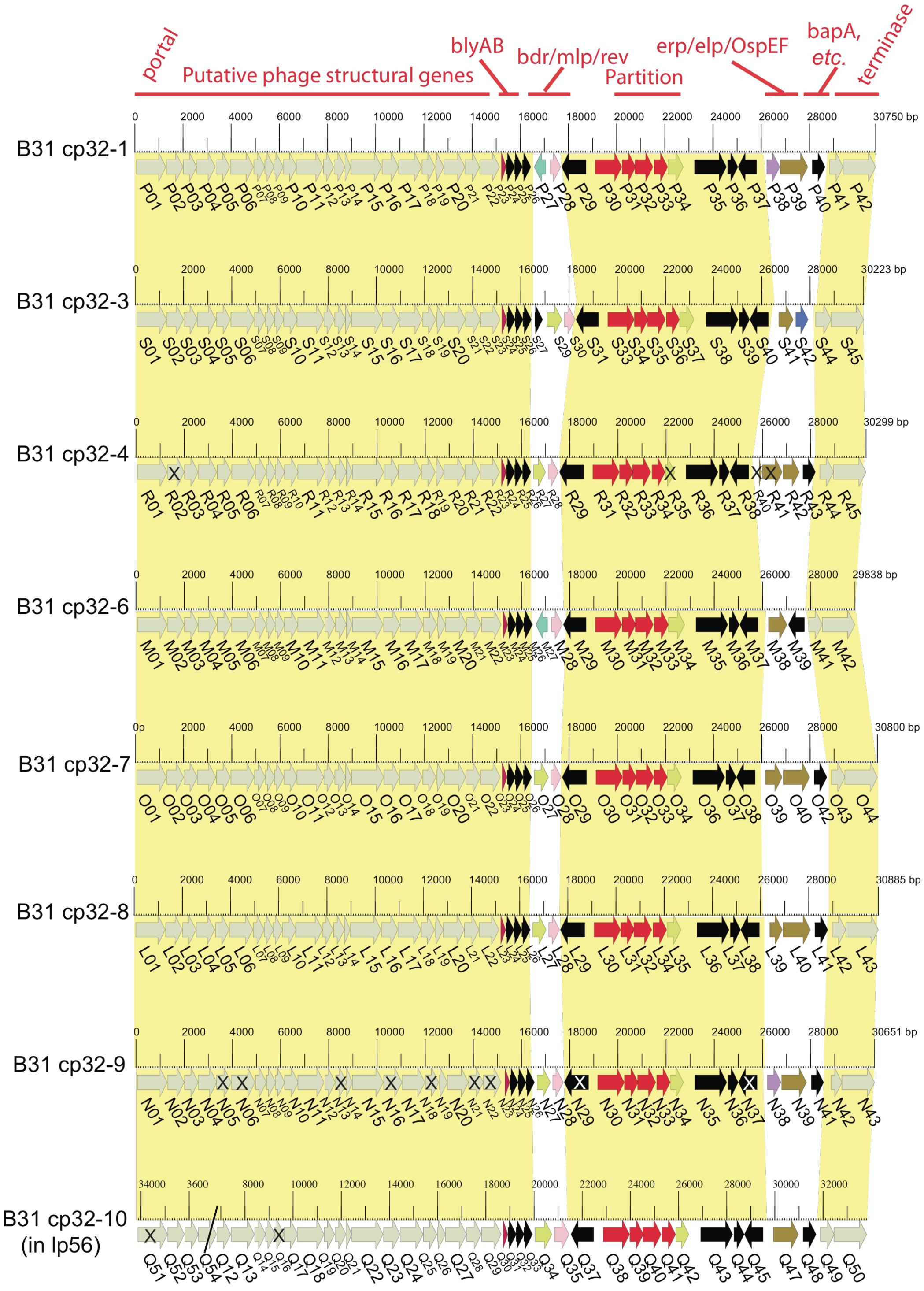

Figure S2E. N40 cp32s

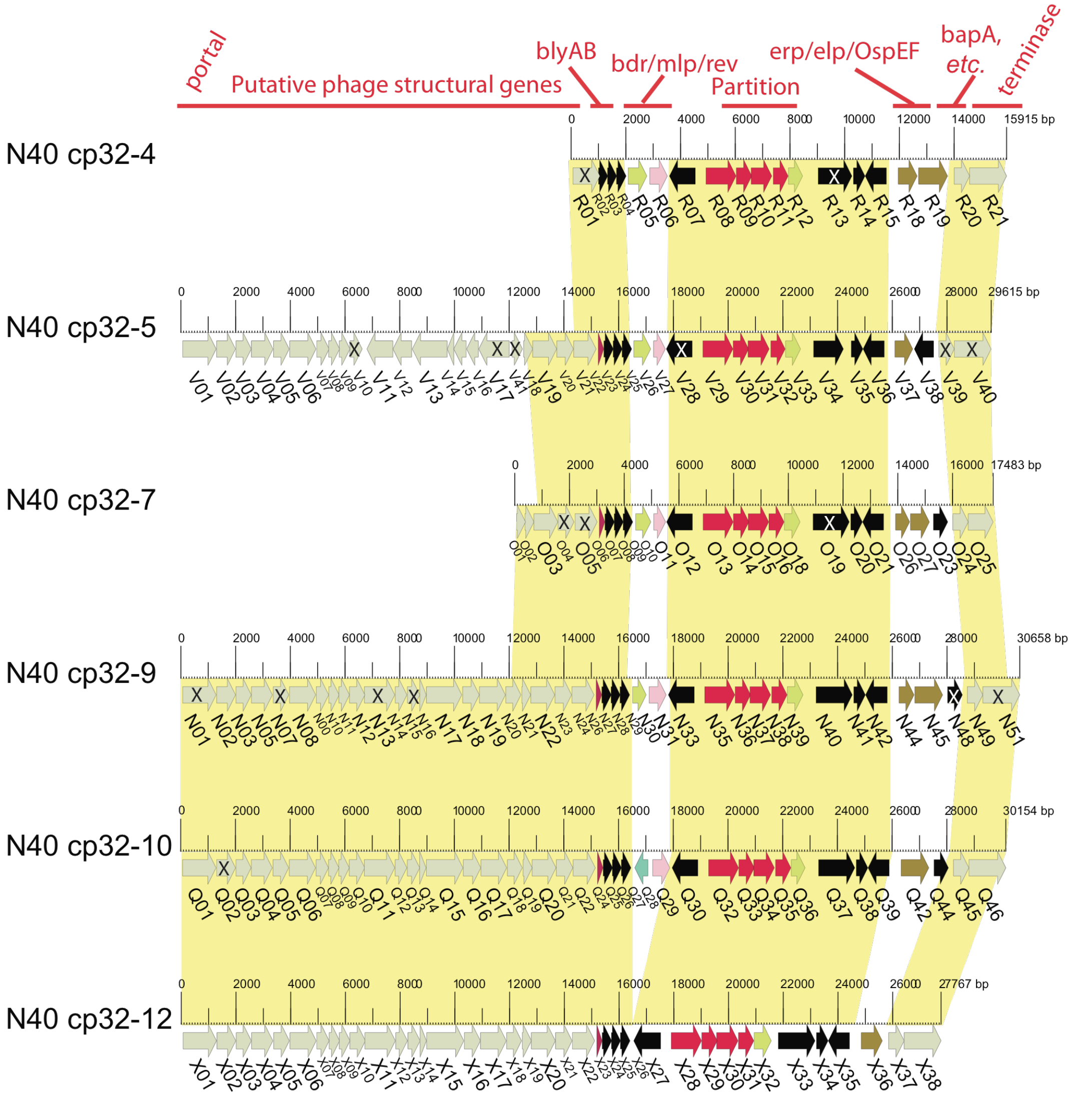

Figure S2F. JD1 cp32s

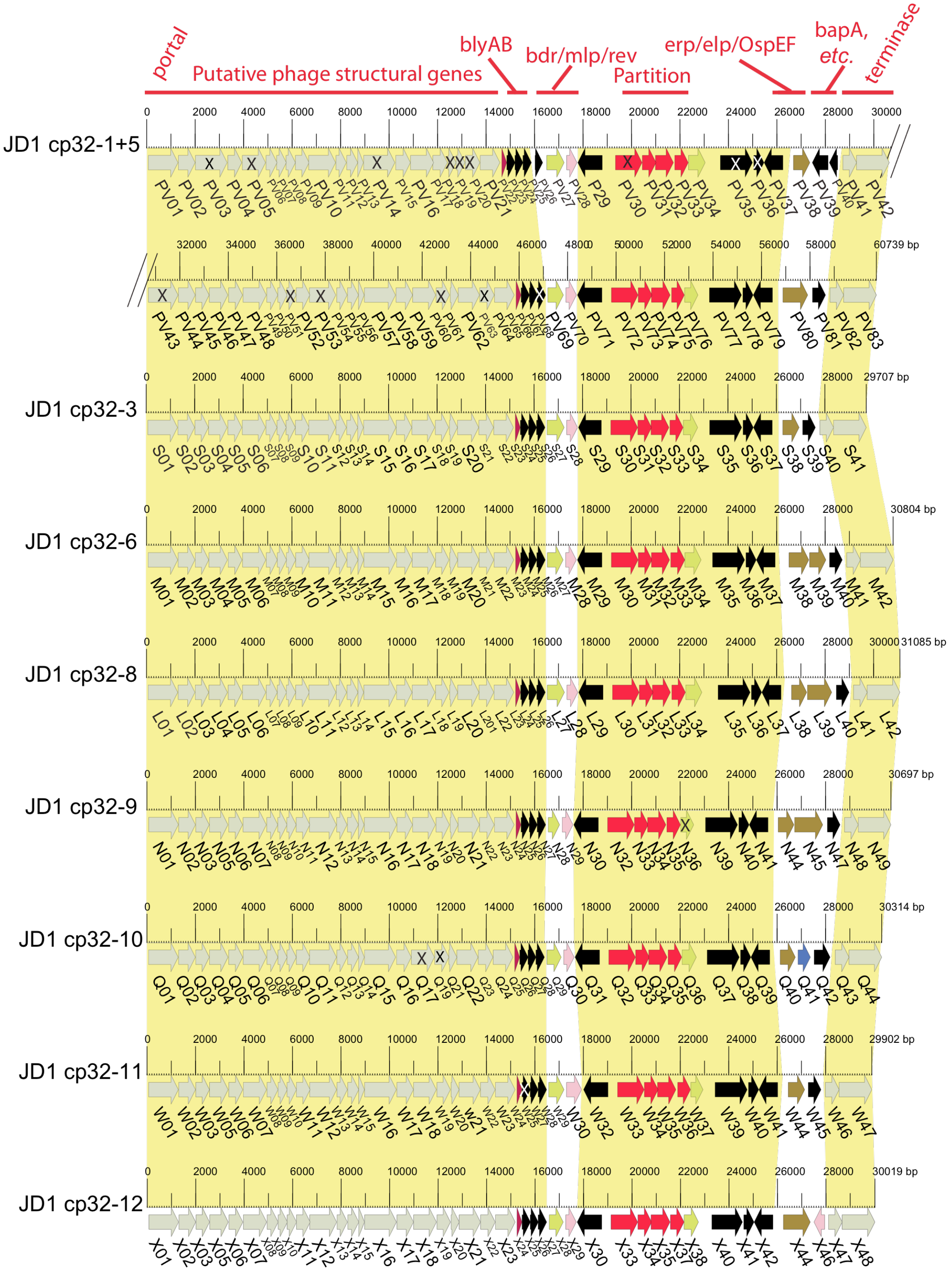

Figure S2G. 297 cp32s

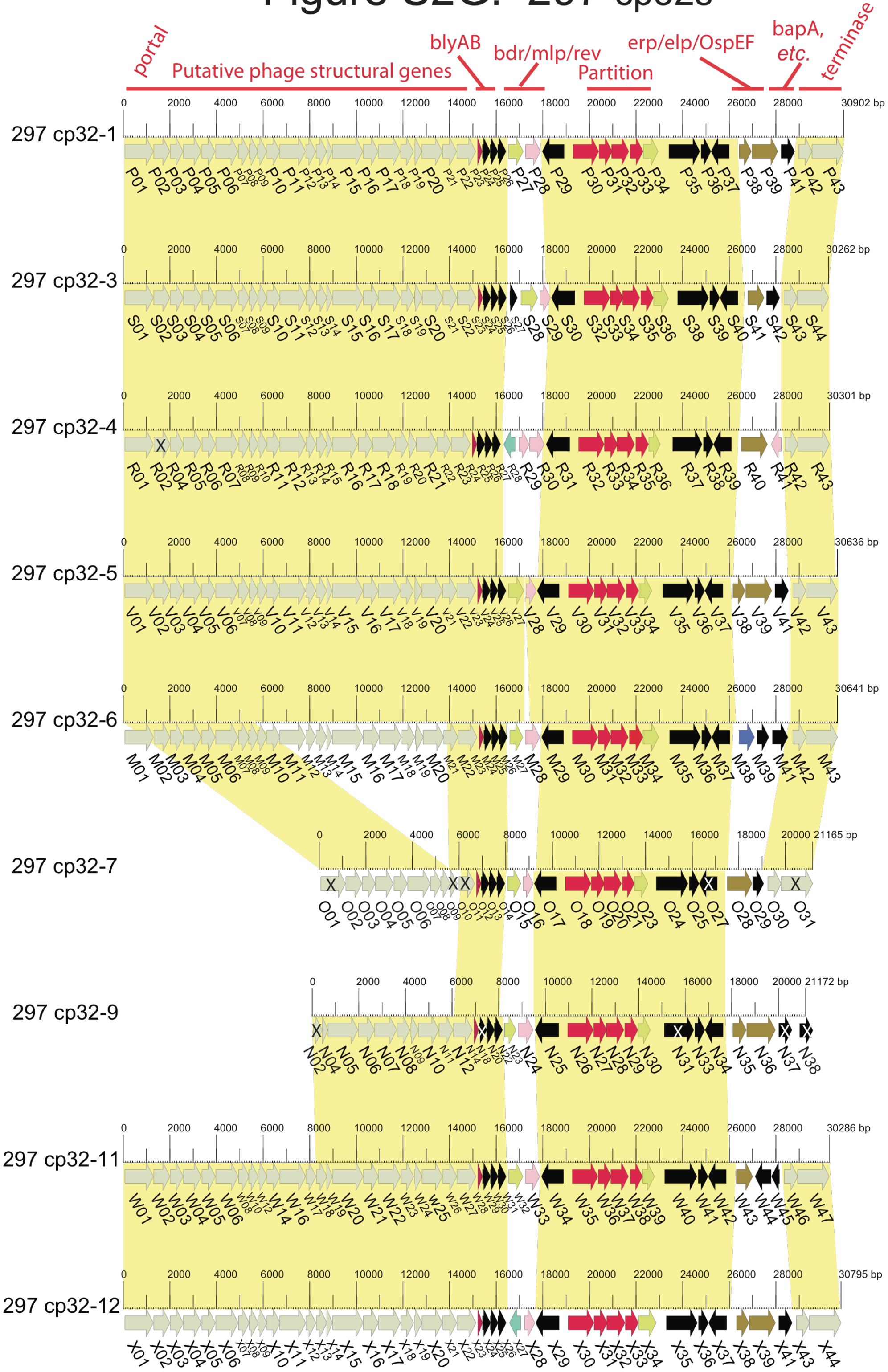

Figure S2H. lp25

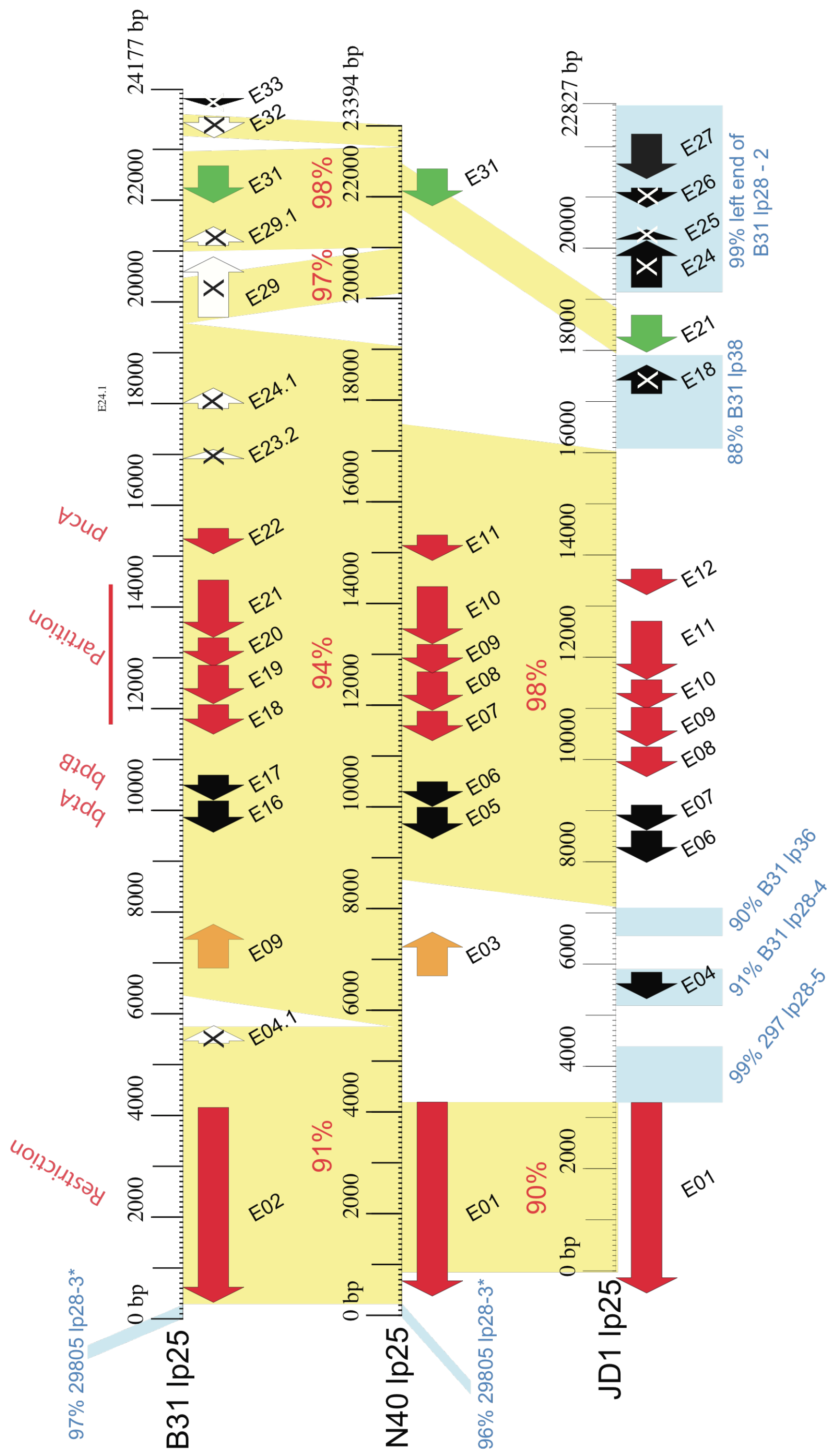

Figure S2I. lp28-1

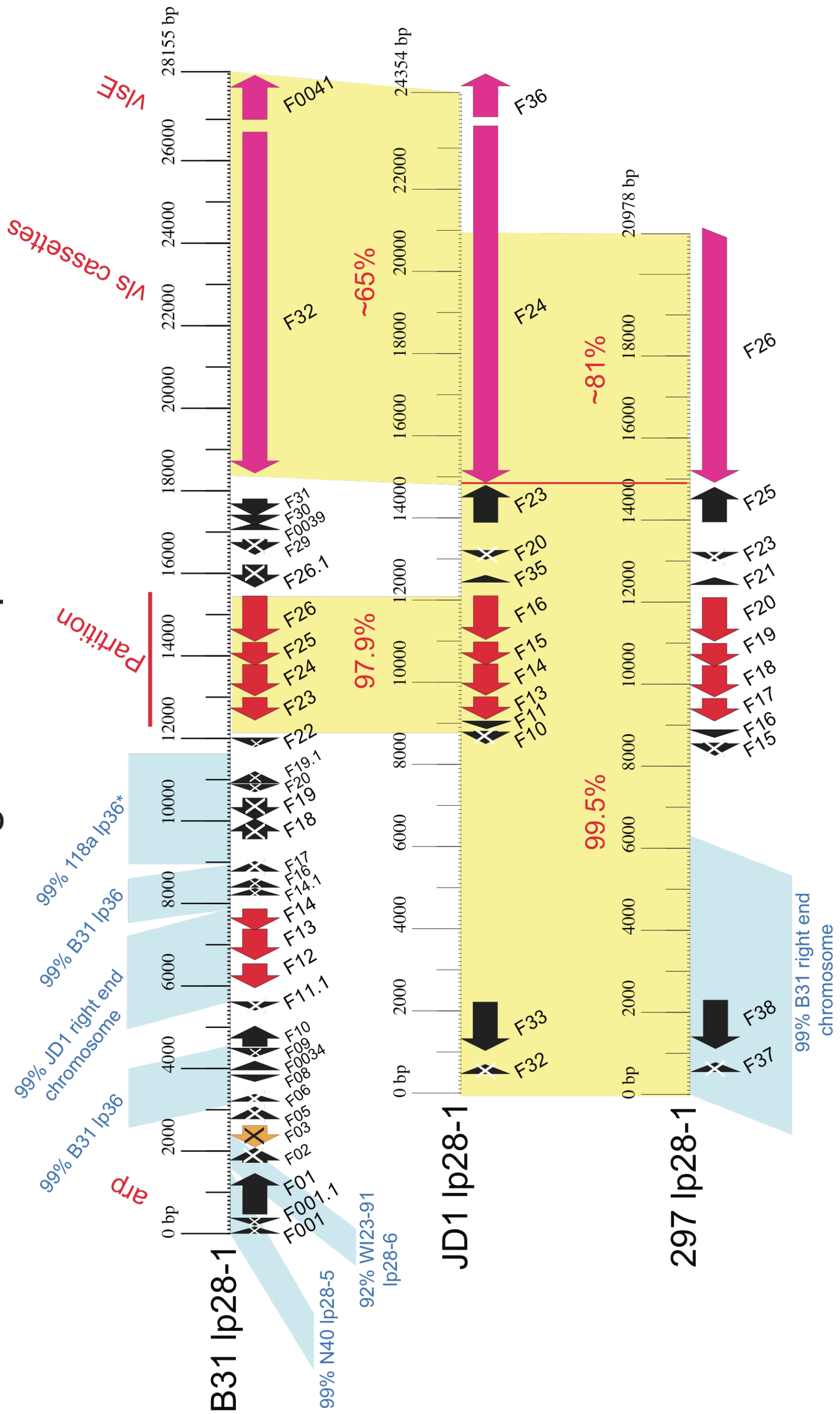

Figure S2J. lp28-3

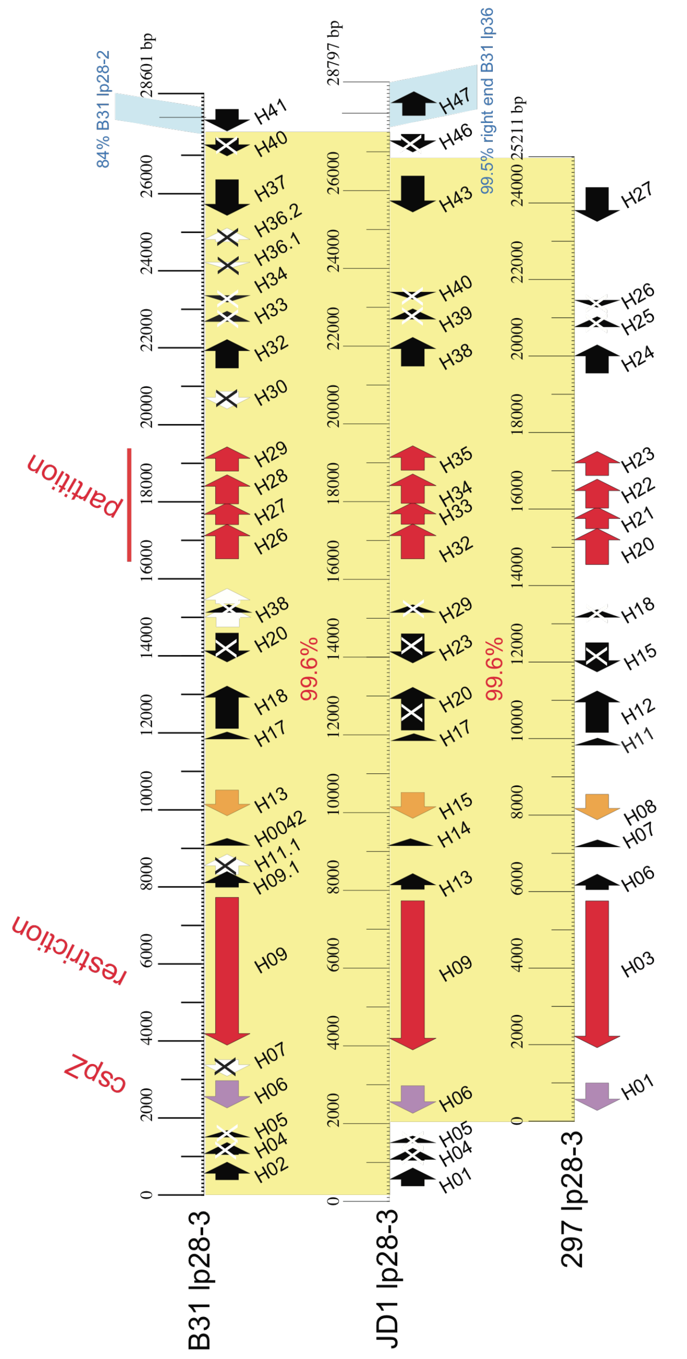

Figure S2K. lp28-4

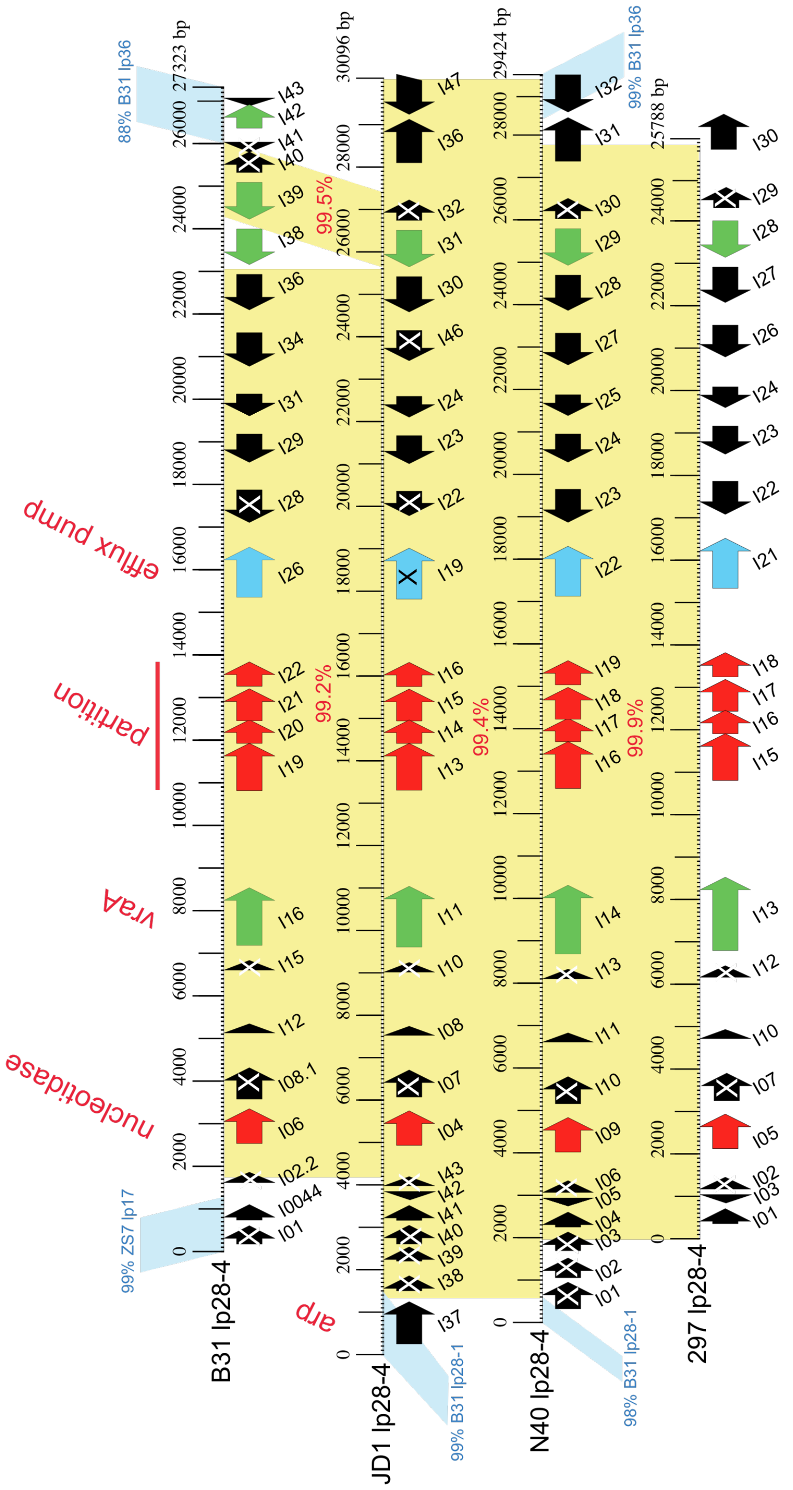

Figure S2L. lp28-2, lp28-6 & lp28-7

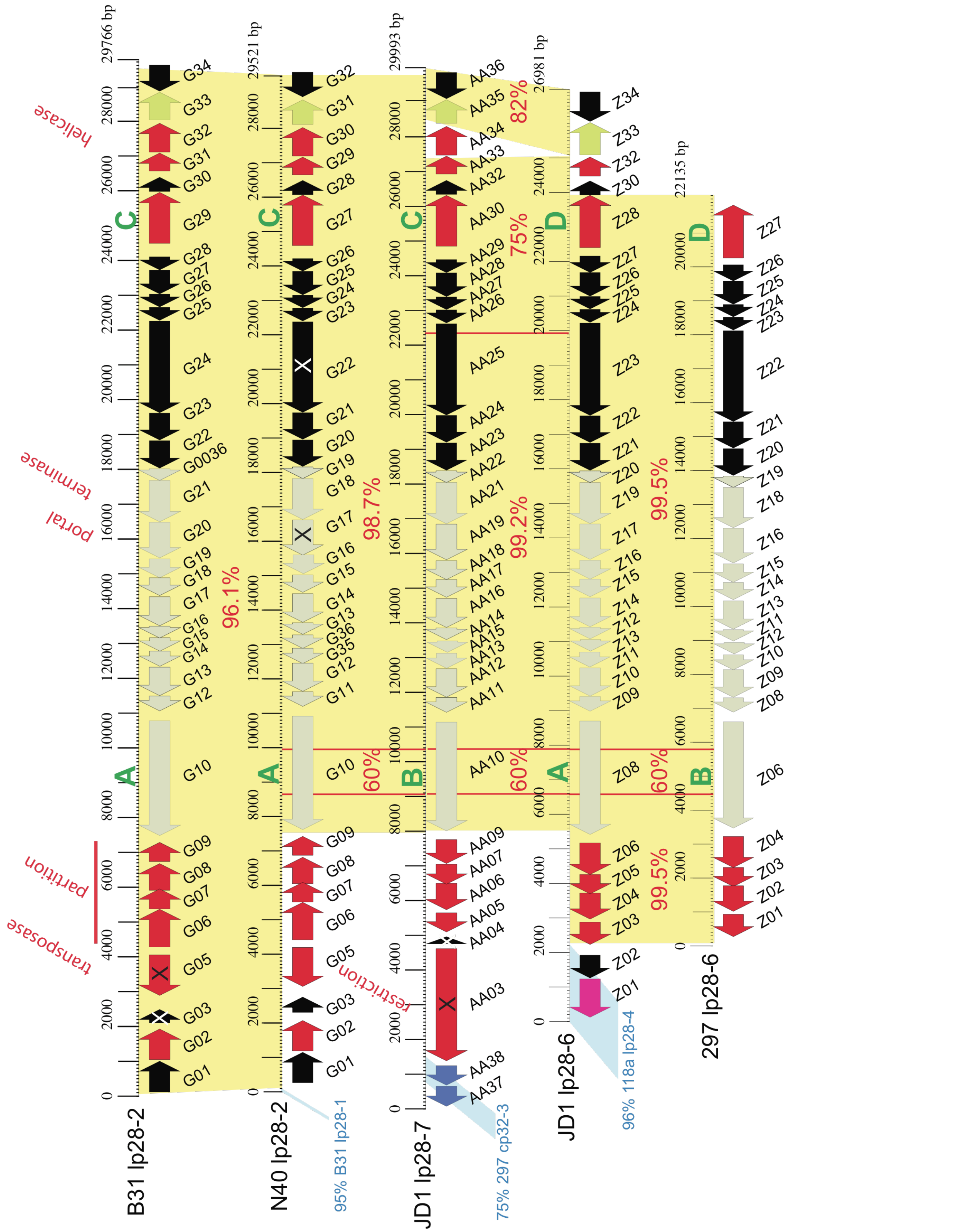

Figure S2M. lp54

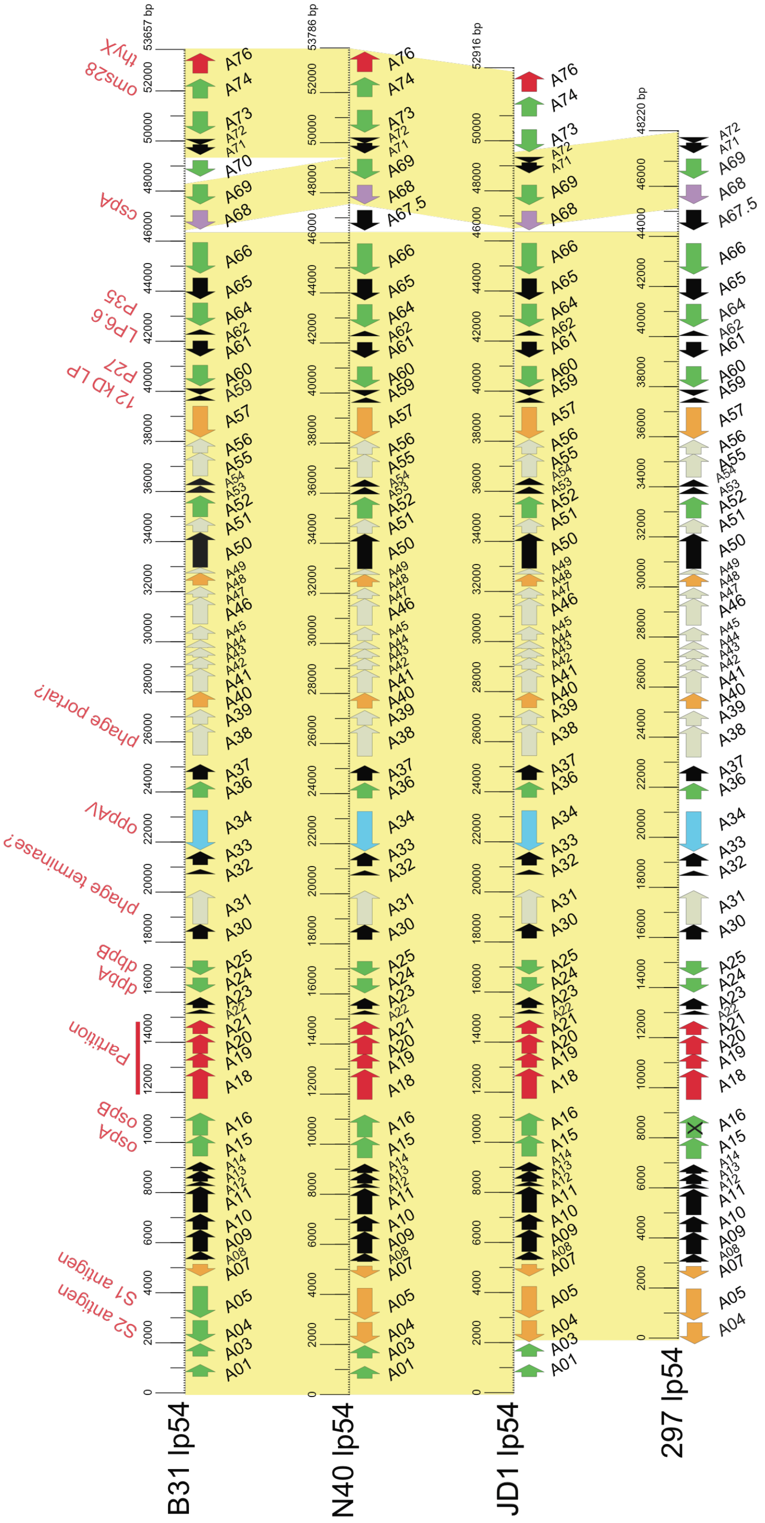

Supplement: Figure S2 — Open reading frame maps for plasmids carried by B. burgdorferi strains B31, N40, JD1 and 297. (PDF) [file pone.0033280.s002.pdf]
