# Supplementary material for: Genome Stability of Lyme Disease Spirochetes: Comparative Genomics of Borrelia burgdorferi Plasmids
Source: PLoS One. 2012 Mar 14;7(3):e33280. doi: 10.1371/journal.pone.0033280 (PMC3303823; doi:10.1371/journal.pone.0033280)
Supplement: Figure S3 — Vls cassette regions. (PDF) [file pone.0033280.s003.pdf]

Figure S3. Vls cassette regions

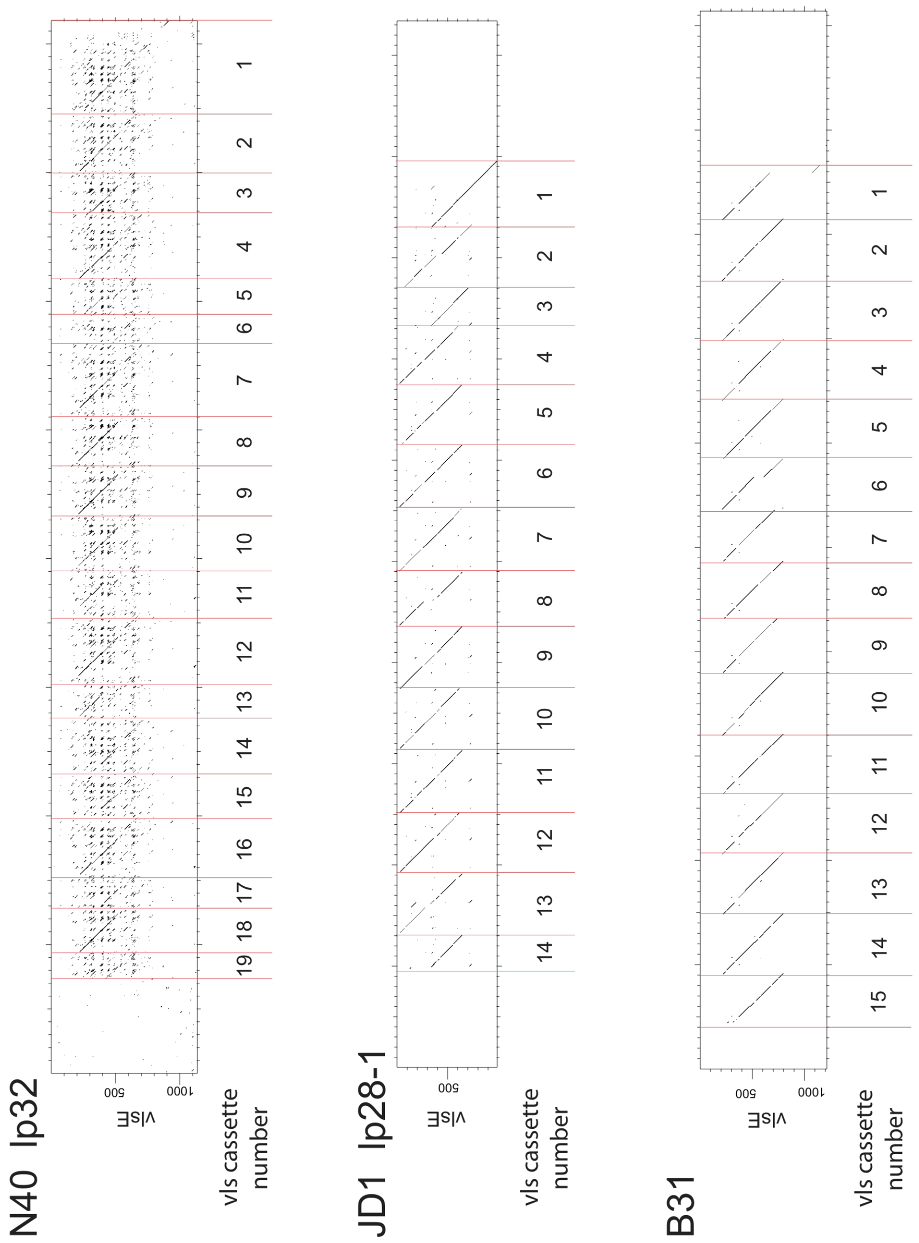

Figure S3. ***B. burgdorferi*** **vls cassette regions.** The cassette regions for N40, JD1 and B31 are shown, with a matrix plot against the cognate *vlsE* gene region (the putative N40 *vlsE* was kindly supplied by X. Wang and J. Weis).
