# Supplementary material for: Genome Stability of Lyme Disease Spirochetes: Comparative Genomics of Borrelia burgdorferi Plasmids
Source: PLoS One. 2012 Mar 14;7(3):e33280. doi: 10.1371/journal.pone.0033280 (PMC3303823; doi:10.1371/journal.pone.0033280)
Supplement: Figure S4 — Ip28-6 copy number. (PDF) [file pone.0033280.s004.pdf]

Figure S4. lp28-6 copy number

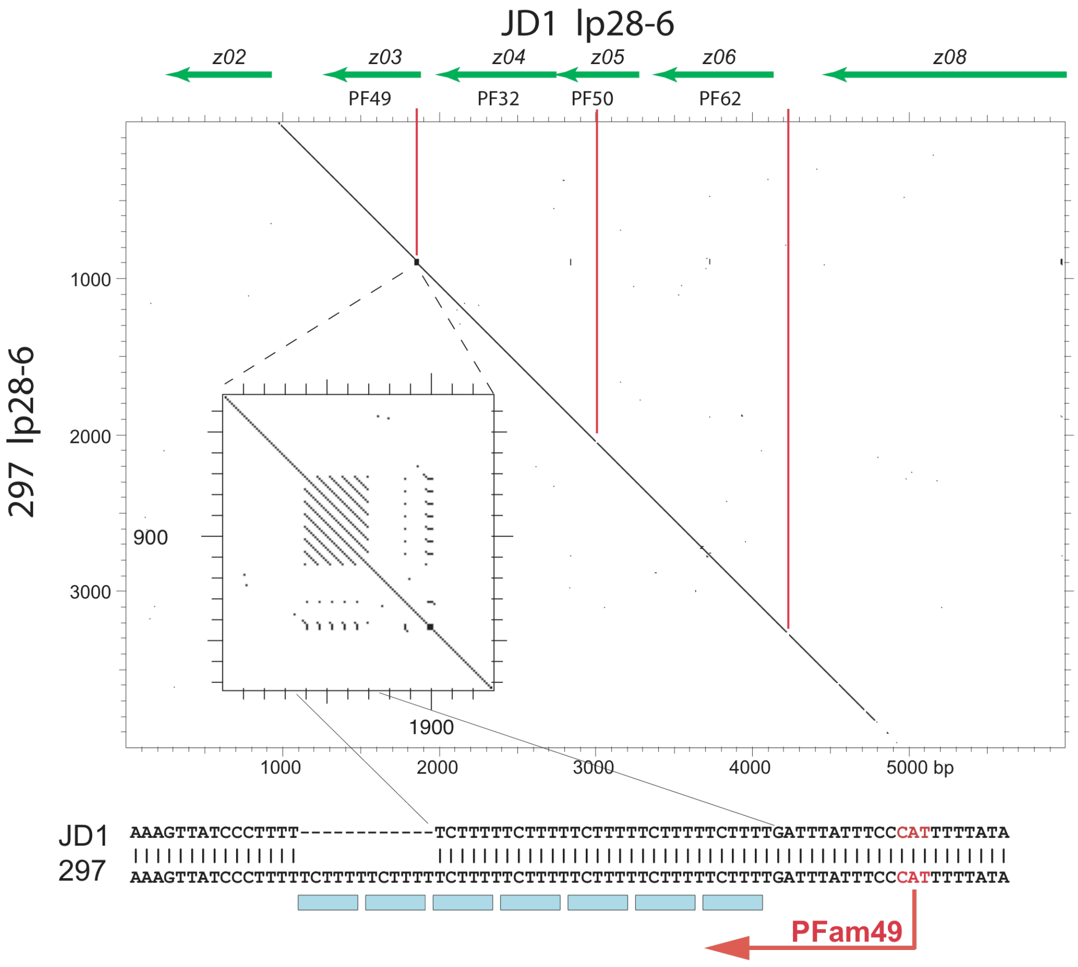

Figure S4. Comparison of JD1 and 297 lp28-6 partition gene regions.

A dot matrix plot (generated by DNA Strider [1] with a 15 identities per 15 bp scanning window). There are only three differences between the two plasmids in the partition gene cluster (*jd1_z03-jd1_z06 vs. 297_z01-297_z04*). The inset shows a magnified view of the difference in *jd1_z03* (PFam49) and the sequences there are compared below, with the predicted *jd1_z03* start codon in red and the tandem six bp Lys-Glu encoding repeats indicated by blue rectangles.

Reference

1. Douglas SE (1994) DNA Strider. A Macintosh program for handling protein and nucleic acid sequences. Methods Mol Biol 25: 181-194.
