# Supplementary material for: Genome Stability of Lyme Disease Spirochetes: Comparative Genomics of Borrelia burgdorferi Plasmids
Source: PLoS One. 2012 Mar 14;7(3):e33280. doi: 10.1371/journal.pone.0033280 (PMC3303823; doi:10.1371/journal.pone.0033280)
Supplement: Figure S5 — Recombination points. (PDF) [file pone.0033280.s005.pdf]

Figure S5. Recombination points

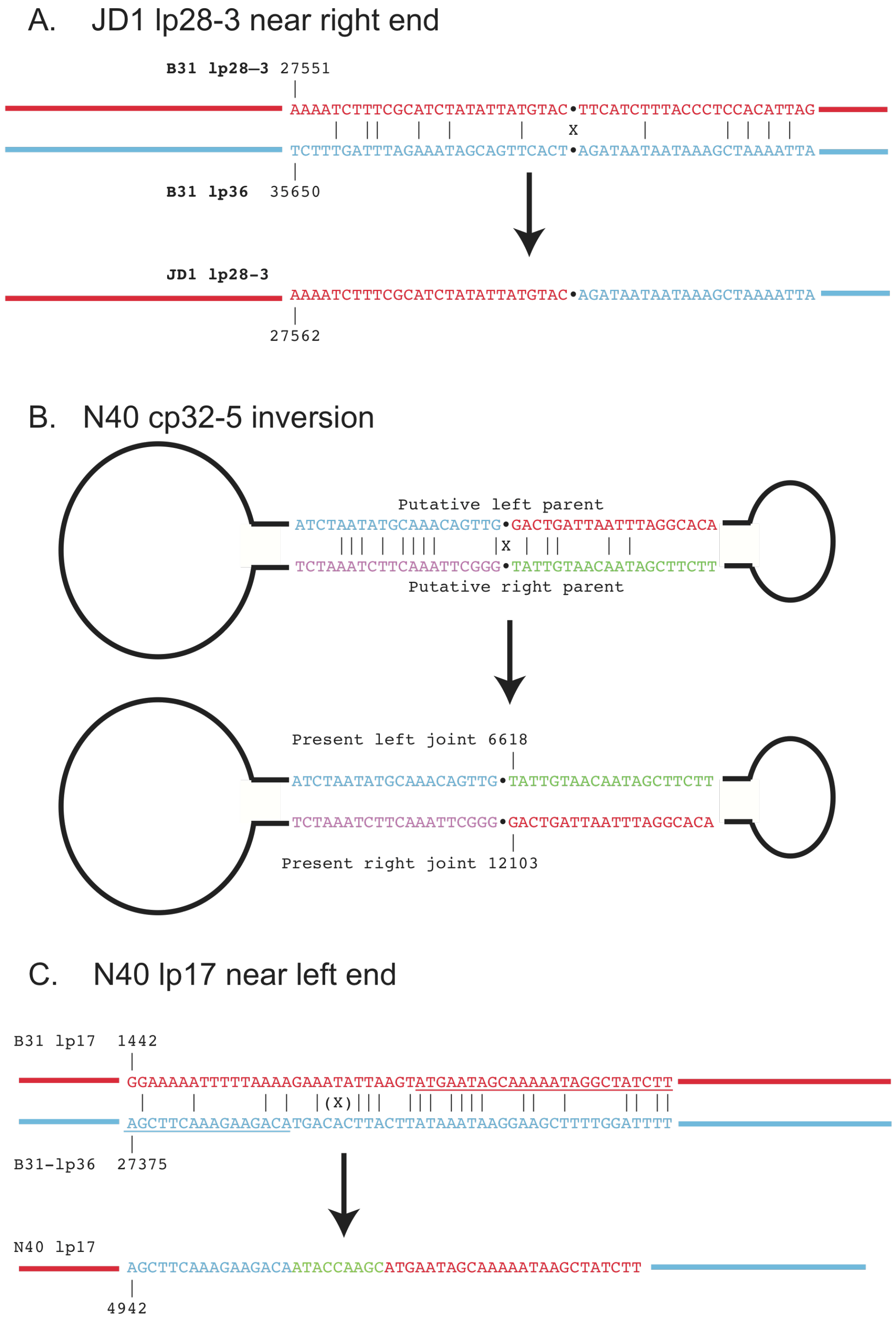

Figure S5. Recombination points

D. Deletion of A70 from JD1 lp54 Pfam54 array

```
        48447
          |
CTCTTTTTT•AAAATATAAA      B31 sequence at between A69 and A70
  |||      ||    ||
GACTTAAAG•AATTAATGTC      B31 sequence in A70
          |
        49145

CTCTTTTTT•T•AATTAATGTC    JD1 sequence at deletion site
          |
        48410
```

E. Deletion of A70 from N40 lp54 Pfam54 array

```
             48415
               |
CTGGGCTGTAAGC•TTAAAAAAAAGAGAA     B31 sequence between A69 and A70
|||||||||||||  ||||||||||||||
CTGGGCTGTAAGC•AAAAAAAAAAGAGAA     B31 sequence between A70 and A71
               |
             49490

CTGGGCTGTAAGC•AAAAAAAAAAAGAGAA    N40 sequence at deletion site
               |
             48414
```

Figure S5. Recombination points. Recombination points (denoted by bullets, •) are shown for several non-homologous events in JD1 and in N40 as well as a homologous event in N40.

A. **JD1 lp28-3 near right end.** The two putative parents appear to be unrelated in sequence, so this recombination event did *not* occur in a region of short sequence similarity between the exchange points. In this case it is not possible to know if the exchange occurred in the direction indicated or if one of the putative "parents" was generated by recombination between the other putative parent and product shown here; however, the directionality does not affect the conclusion that the event did not utilize homologous recombination.

B. **N40 cp32-5 inversion.** Since this region is very highly conserved in many other cp32 plasmids, the presence of rearranged genes strongly indicates that this inversion occurred in this cp32-5, and the near identity of these regions in the other cp32s allows precise deduction of the breakpoints that generated the inversion. Note that although the parts of the plasmids whose sequences are not shown are represented by single black lines they in fact represent both strands of the dsDNA, and that because of the topology, the two sequences shown are actually on different strands (both shown 5′-to-3′ left to right). This recombination event did not occur between regions of even short sequence similarity.

C. **N40 lp17 near left end.** The two putative parents appear to be unrelated in sequence, so this recombination event did *not* occur in a region of short sequence similarity between the exchange points. In this case the putative non-homologous event was more complex in that nine bp (indicated in green) of unknown origin are present between the two putative parental sequences.

D. **Deletion of A70 from JD1 lp54 Pfam54 array.** A non-homologous event that apparently generated an additional "T" (green) at the point of recombination.

E. **Deletion of A70 from N40 lp54 Pfam54 array.** An homologous event that apparently generated an additional "A" (green) at the point of recombination.
