# Supplementary material for: Genome Stability of Lyme Disease Spirochetes: Comparative Genomics of Borrelia burgdorferi Plasmids
Source: PLoS One. 2012 Mar 14;7(3):e33280. doi: 10.1371/journal.pone.0033280 (PMC3303823; doi:10.1371/journal.pone.0033280)
Supplement: Figure S6 — Neighbor-joining trees of proteins encoded by B. burgdorferi cp32 variable regions. (PDF) [file pone.0033280.s006.pdf]

Figure S6A

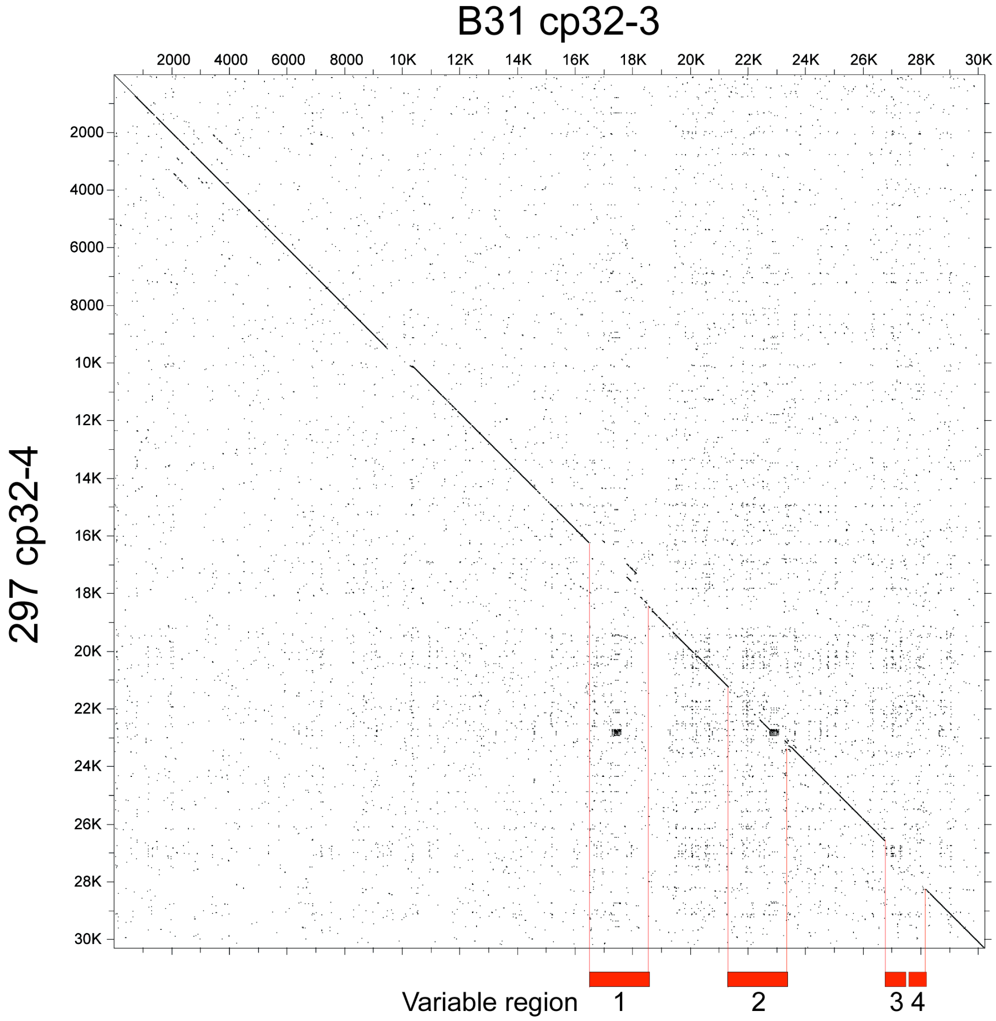
B31 cp32-3
2000
4000
6000
8000
10K
12K
14K
16K
18K
20K
22K
24K
26K
28K
30K
297 cp32-4
Variable region
1
2
3
4

Figure S6B. Variable regions of the cp32 plasmids

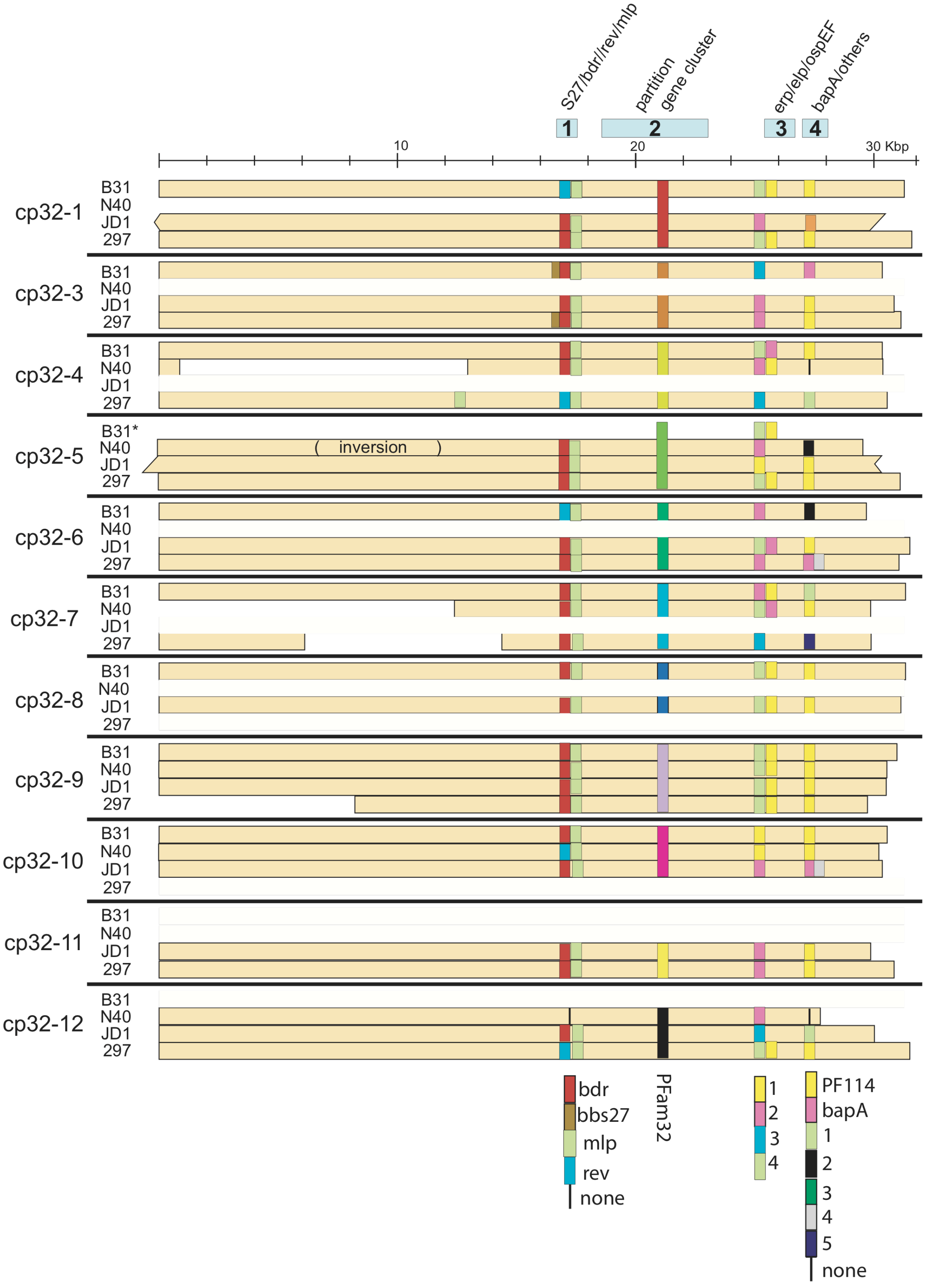

Figure S6C. Mlp Protein Tree

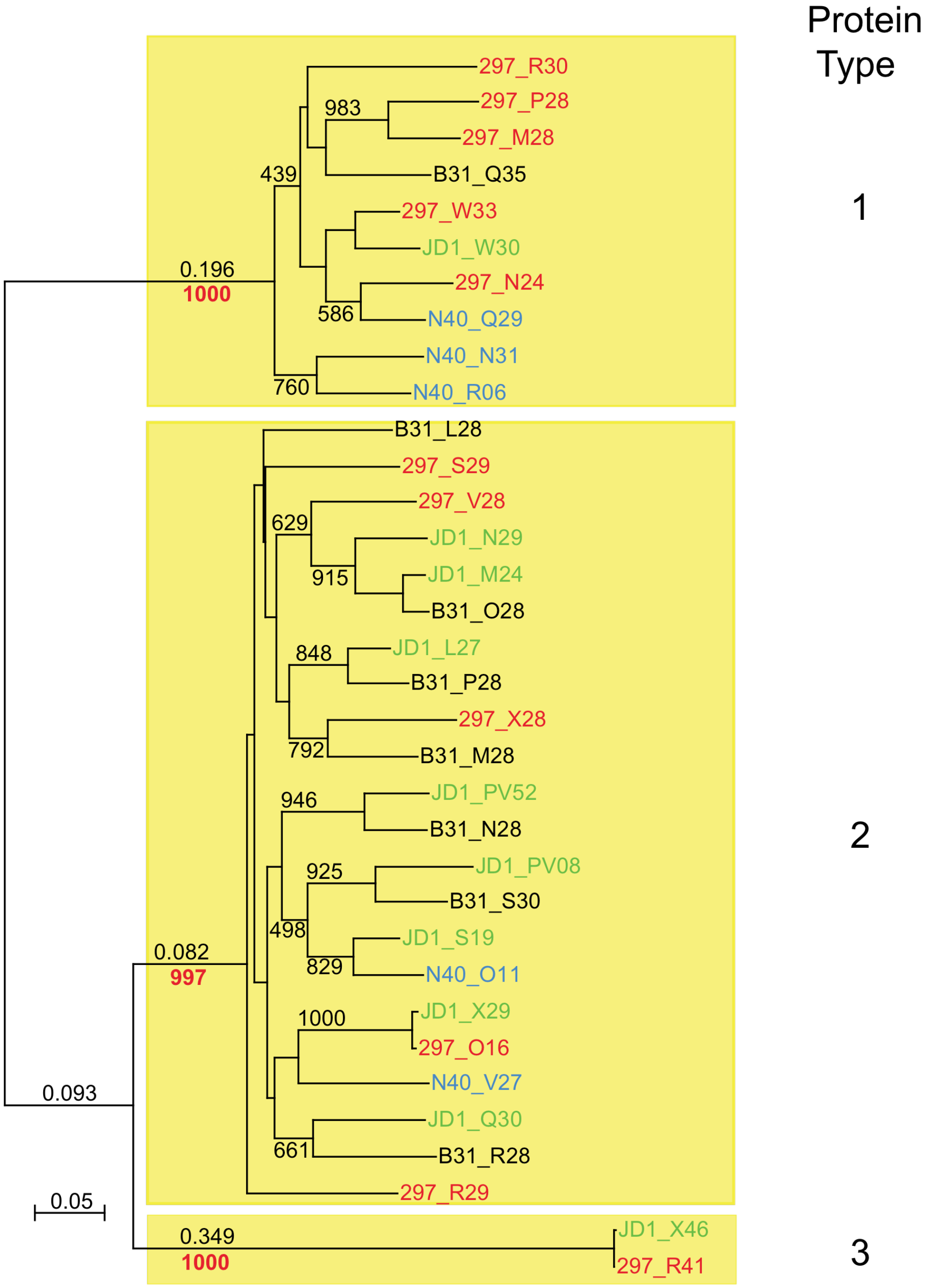

Figure S6D. Bdr Protein Tree

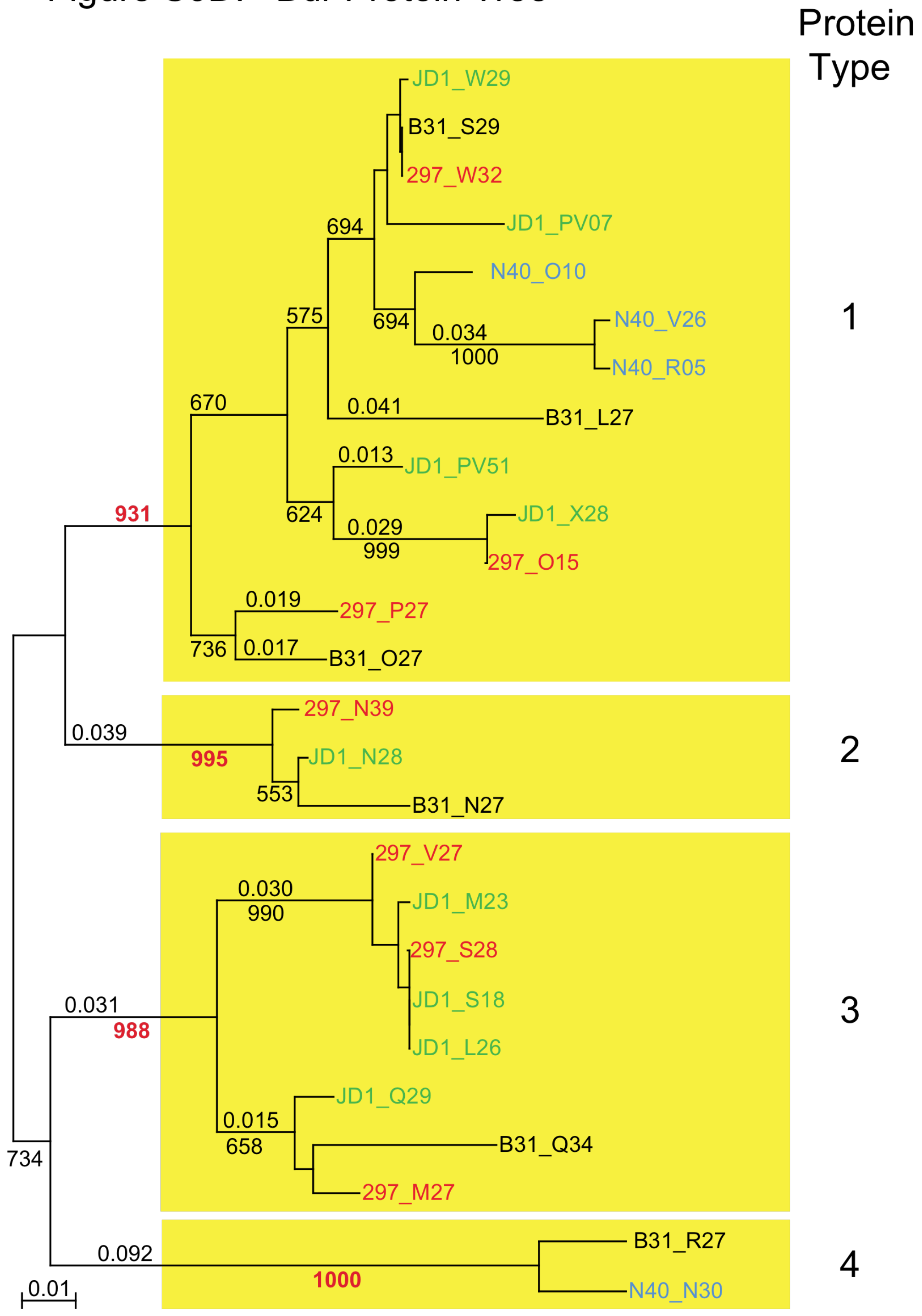

Figure S6E. Erp/Elp/OspEF Tree

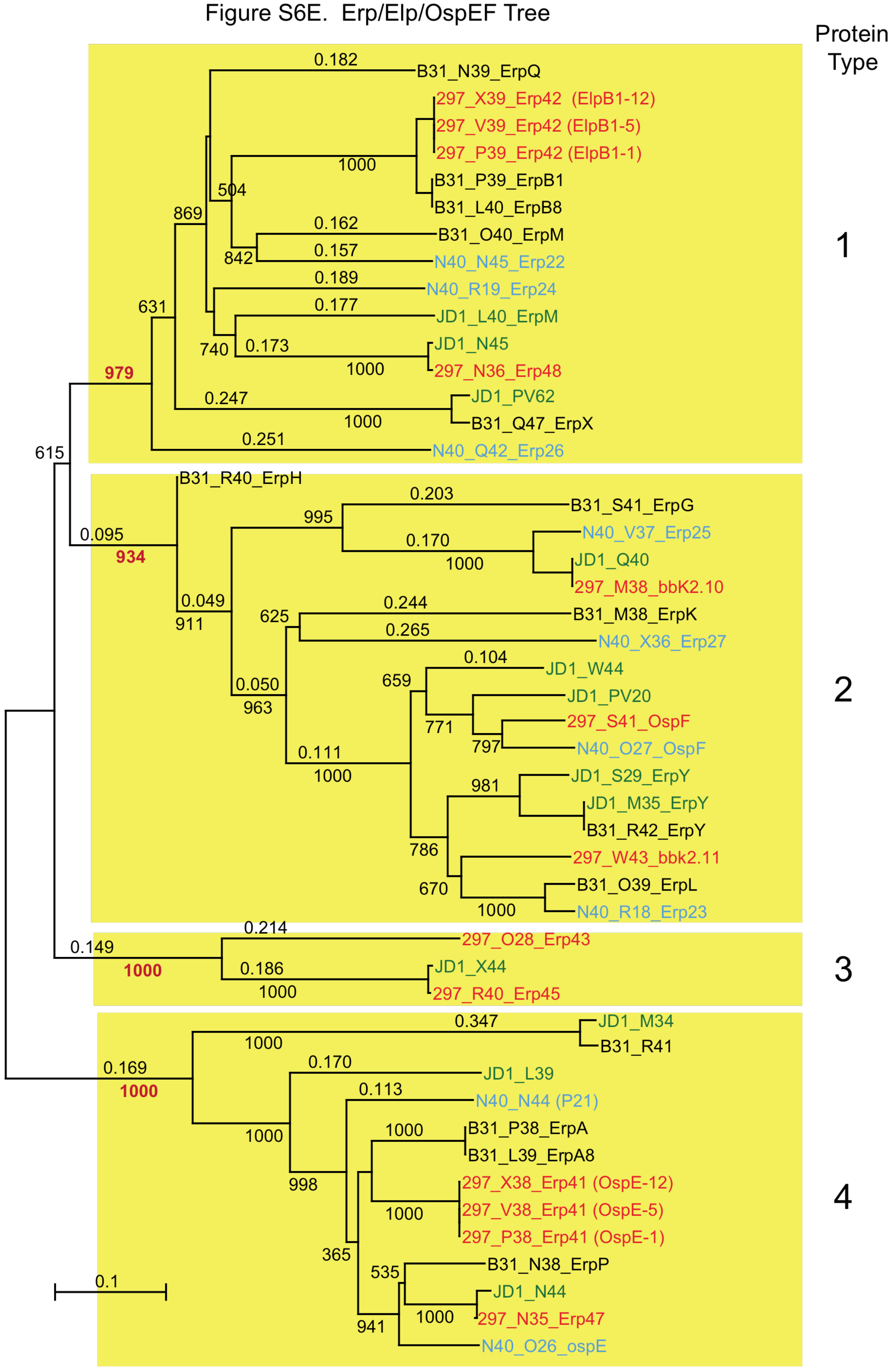

Figure S6F. PFam114 Protein Tree

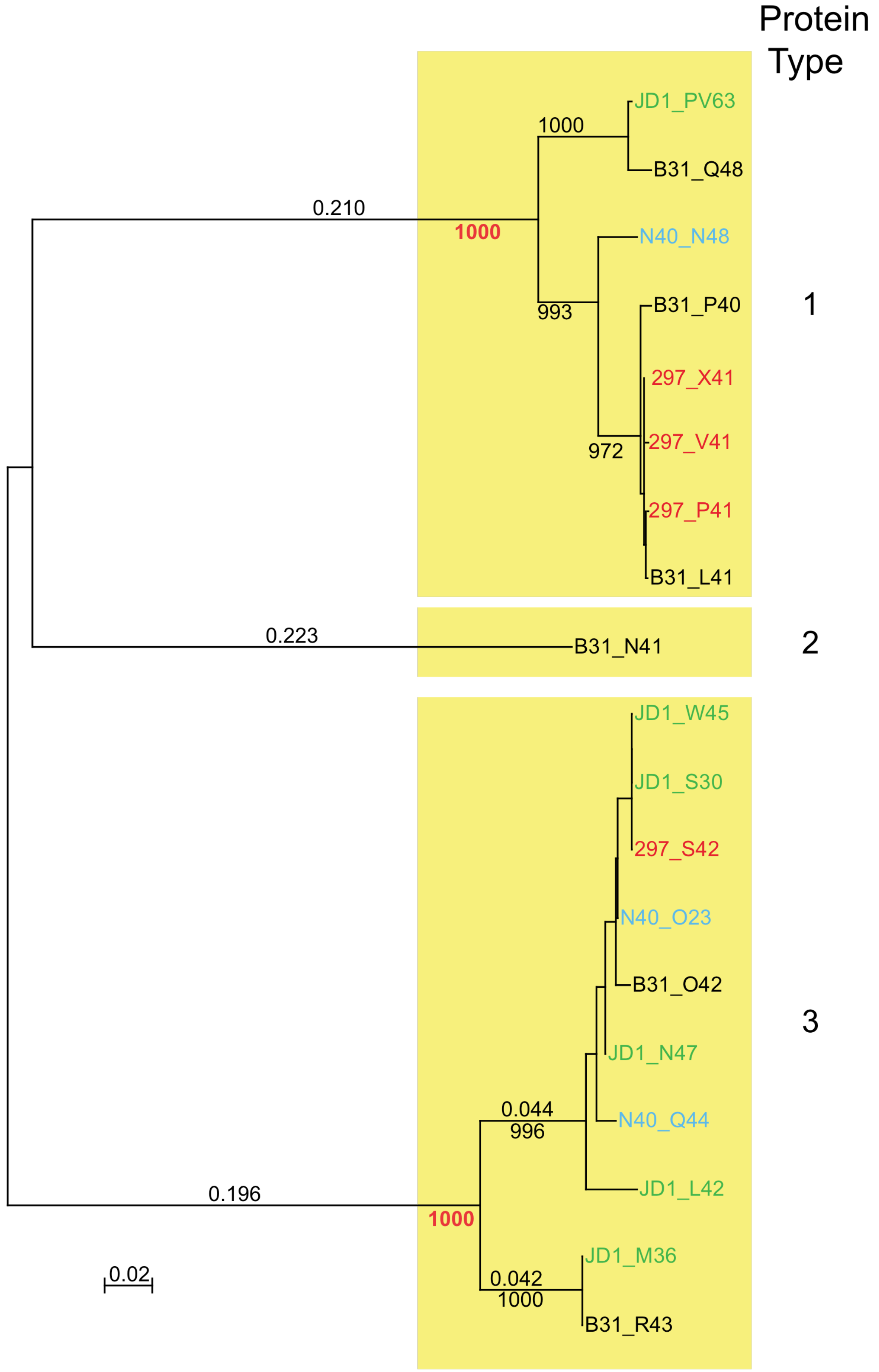

Figure S6. Neighbor-joining trees of proteins encoded by *B. burgdorferi* cp32 variable regions.

ClustalX-2 [1] was used to create neighbor-joining trees for the proteins encoded the genes the four cp32 "variable regions", and yellow boxes enclose robust "sequence types" in the trees shown. Bootstrap values for 1000 trials are shown as numbers between 1 and 1000, and these values for the branches that make up the sequence types defined here are in bold red type. In each case a fractional sequence difference scale is shown at the lower left and some such distance values (between 0 and 1) are shown in the trees. Protein names at the ends of the branches have the form "strain_protein name_previously given name". The relationships within the *bdr*, *mlp* and *erp/elp/ospEF* gene groups are complex and alignments are not always unambiguous due to direct repeats and apparent recombination events that have created hybrid genes, however, since our purpose is not to delineate the history or function of these genes, but only to group them into general "types", these difficulties were ignored and the Clustal alignments were used to build the trees. The proteins encoded by homologs of *s27* (region 1), *rev* (region 1) and *bapA* (region 4) genes are all so similar that subtypes were not evident, and trees are not shown.

A. **Variable regions of the cp32 plasmids.** Matrix comparison plot of two arbitrarily chosen cp32s created by DNA Strider [2] with a 17 matches per 23 nucleotide scanning window. The four variable regions used in this analysis are indicated below. There are a few other regions that show some variation, and comparison of this pair of cp32 plasmids shows that the sequence around 10 kbp is quite different between these two particular plasmids.

B. **Structure of the cp32 plasmids.** The thirty-one completely sequence cp32 plasmids are shown as tan horizontal bars. The cp32-1 and cp32-5 bars in JD1 are fused as one double-size circular plasmid named cp32-1+5 (see text). The asterisk (*) notes a plasmid that was lost from the strain before its genome was sequenced). The plasmids are largely homologous throughout their lengths and are oriented so that homologous regions are aligned vertically. Gaps in these bars show the position of large deletions in some plasmids. Above, blue bars denote the four most variable regions in these plasmids, and below colored boxes denote gene differences in each of these regions; the numbers associated with the latter are defined as in the footnote to Table 3 in the text. Two plasmids, B31 cp32-7 and cp32-9, have previously been called cp18-1 and cp18-2, respectively [3], and N40 cp32-7 has previously been called cp18 [4].

C. **A neighbor joining tree of the *mlp* proteins encoded in region 1.** All the *mlp* genes are in region 1 except type 3 genes which are in region 4. See Cainamo *et al.* [3] for a more detailed discussion of these genes.

D. **A neighbor joining tree of the *bdr* (<u>B</u>orrelia <u>d</u>irect <u>r</u>epeat) proteins encoded in region 1.** Note that there are additional *bdr* genes near region 2 that are ignored in this analysis; see Zuckert and Barbour *et al.* [5] and Carlyon *et al.* [6] for more detailed discussions of these genes.

E. **A neighbor joining tree of the *erp/elp/ospEF* proteins encoded in region 3.** These proteins include B31_N38 and B31_P38 (both sequence type 4 in the tree), which are close relatives of genes in other strains that affect the

mammalian complement fixation [7]. See Stevenson and Miller [8] for a more detailed discussion of these genes.

F. **A neighbor joining tree of paralogous family PFam144 proteins encoded in region 4.** These genes have no known or postulated function.

References

1. Jeanmougin F, Thompson JD, Gouy M, Higgins DG, Gibson TJ (1998) Multiple sequence alignment with Clustal X. Trends Biochem Sci 23: 403-405.
2. Douglas SE (1994) DNA Strider. A Macintosh program for handling protein and nucleic acid sequences. Methods Mol Biol 25: 181-194.
3. Caimano MJ, Yang X, Popova TG, Clawson ML, Akins DR, *et al.* (2000) Molecular and evolutionary characterization of the cp32/18 family of supercoiled plasmids in *Borrelia burgdorferi* 297. Infect Immun 68: 1574-1586.
4. Stevenson B, Casjens S, van Vugt R, Porcella SF, Tilly K, *et al.* (1997) Characterization of cp18, a naturally truncated member of the cp32 family of *Borrelia burgdorferi* plasmids. J Bacteriol 179: 4285-4291.
5. Zuckert WR, Meyer J, Barbour AG (1999) Comparative analysis and immunological characterization of the *Borrelia* Bdr protein family. Infect Immun 67: 3257-3266.
6. Carlyon JA, Roberts DM, Marconi RT (2000) Evolutionary and molecular analyses of the *Borrelia bdr* super gene family: delineation of distinct sub-families and demonstration of the genus wide conservation of putative functional domains, structural properties and repeat motifs. Microb Pathog 28: 89-105.
7. Kraiczy P, Hartmann K, Hellwage J, Skerka C, Kirschfink M, *et al.* (2004) Immunological characterization of the complement regulator factor H-binding CRASP and Erp proteins of *Borrelia burgdorferi*. Int J Med Microbiol 293 Suppl 37: 152-157.
8. Stevenson B, Miller JC (2003) Intra- and interbacterial genetic exchange of Lyme disease spirochete *erp* genes generates sequence identity amidst diversity. J Mol Evol 57: 309-324.
