# Supplementary material for: Genome Stability of Lyme Disease Spirochetes: Comparative Genomics of Borrelia burgdorferi Plasmids
Source: PLoS One. 2012 Mar 14;7(3):e33280. doi: 10.1371/journal.pone.0033280 (PMC3303823; doi:10.1371/journal.pone.0033280)
Supplement: Figure S7 — Orthologous non-coding DNA in Ip28-4. (PDF) [file pone.0033280.s007.pdf]

Figure S7. Orthologous non-coding DNA in lp28-4

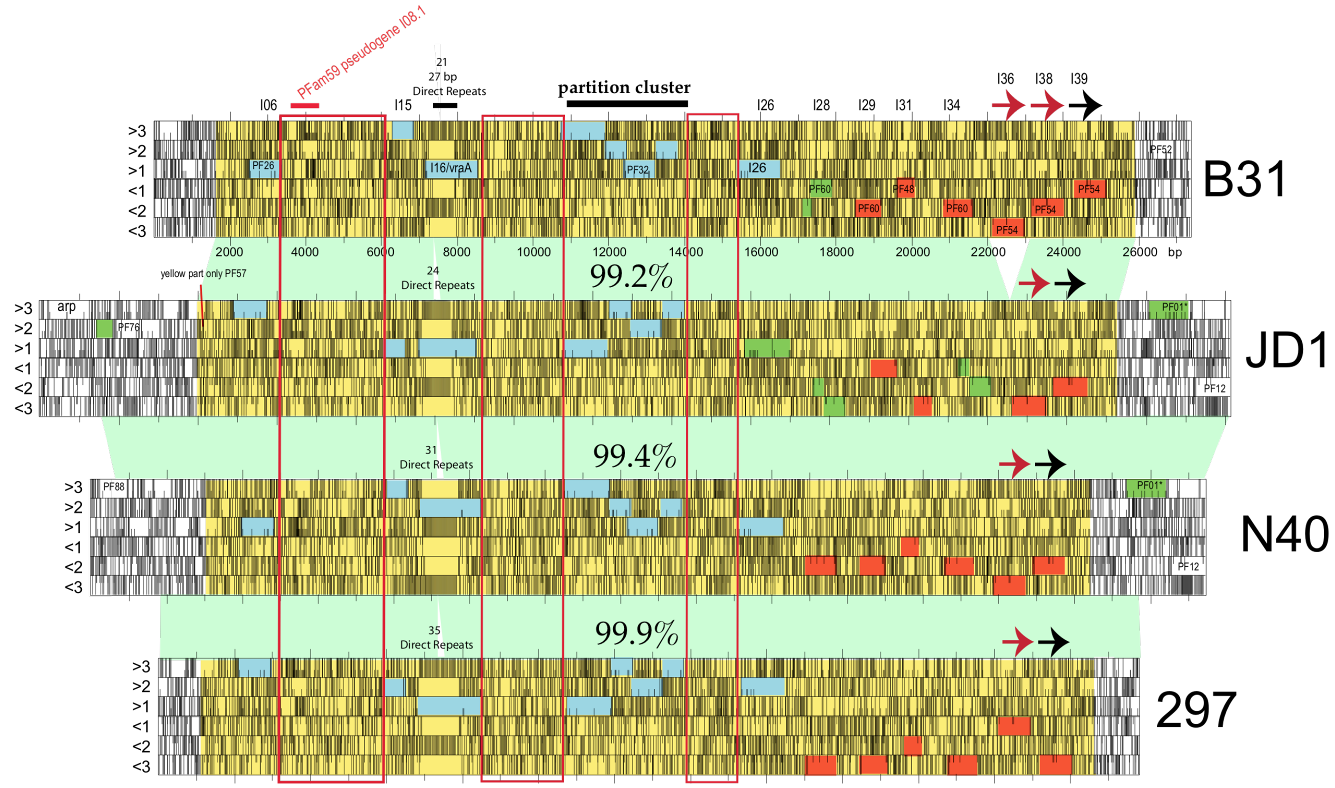

Figure S7. Orthologous non-coding DNA in lp28-4. Translational six-reading frame diagrams are shown for the extremely similar lp28-4 plasmids from strains B31, JD1, N40 and 297. Translation is left to right in top three frames where the longer open reading frames are blue, and right to left in bottom three where the longer open reading frames are red. Genes with broken open reading frames are indicated in green. Long vertical lines in each frame represent stop codons and short vertical lines indicate methionine codons. The red rectangles enclose regions with no significant open reading frames (except perhaps in the PFam59 pseudogene).
