# Supplementary material for: Genome Stability of Lyme Disease Spirochetes: Comparative Genomics of Borrelia burgdorferi Plasmids
Source: PLoS One. 2012 Mar 14;7(3):e33280. doi: 10.1371/journal.pone.0033280 (PMC3303823; doi:10.1371/journal.pone.0033280)
Supplement: Table S1 — B31, JD1, N40 and 297 replicon sizes and accession numbers. (PDF) [file pone.0033280.s008.pdf]

Table S1. B31, JD1, N40 and 297 replicon sizes and accession numbers

	B31						N40					
	sequence (bp)[a]	missing terminal bp 5'	3'	Measured size (kbp)[b]	annotated ORFs	accession No.	sequence (bp)	approximate missing bp 5'	3'	Measured size (kbp)[b]	annotated ORFs	accession No.
chromosome	910724	0	0	–	825	AE000783	902191	250	100	–	814	CP002228
lp5	5228	21[c]	7[c]	5	6	AE001583	–	–	–	–	–	–
lp17	16928	0	0	17	19	AE000793	20672	0[d]	14	21	19	CP002241
lp21	18809	0	0	19	9	AE001582	–	–	–	–	–	–
lp25	24407	0	0	25	11	AE000785	23394	100	700	24	10	CP002234
lp28-1	28922	0	0	28	33	AE000794	–	–	–	–	–	–
lp28-2	29925	0	0	30	33	AE000786	29521	200	200	30	33	CP002232
lp28-3	28637	0	0	29	22	AE000784	e	–	–	29	–	–
lp28-4	27421	0	0	27	24	AE000789	29424	200	500	30	25	CP002236
lp28-5	–	–	–	–	–	–	26642	700	400	27.5	26	CP002233
lp28-6	–	–	–	–	–	–	–	–	–	–	–	–
lp28-7	–	–	–	–		–	–	–	–	–	–	–
lp36	36926	0	0	36	36	AE000788	29194	300	2000	31.5	24	CP002230
lp38	38868	0	0	38	38	AE000787	37903	500	300	38.5	30	CP002237
lp54	53678	0	0	54	65	AE000790	53799	6[a]	6[a]	54	65	CP001651
lp56	53029 (22622[g])	0	0	54	67	AE001584	–	–	–	–	–	–
cp9-1	9386	–	–	9.5	10	AE000791	8722	–	–	9	9	CP002232
cp26	26498	–	–	26	26	AE000792	26498	–	–	26	26	CP002239
cp32-1	30750	–	–	31	42	AE001575	–	–	–	–	–	–
cp32-3	30223	–	–	30	42	AE001576	–	–	–	–	–	–
cp32-4	30299	–	–	30	43	AE001577	15915	–	–	16	19	CP002235
cp32-5	e	–	–	30	–	–	29615	–	–	30	41	CP002227
cp32-6	29838	–	–	30	41	AE001578	–	–	–	–	–	–
cp32-7	30800	–	–	31	42	AE001579	17483	–	–	17	25	CP002229
cp32-8	30885	–	–	31	42	AE001580	–	–	–	–	–	–
cp32-9	30651	–	–	31	41	AE001581	30658	–	–	31	42	CP002242
cp32-10	(30349[g])	–	–	–	–	AE001584	30154	–	–	30	41	CP002240
cp32-11	–	–	–	–	–	–	–	–	–	–	–	–
cp32-12	–	–	–	–	–	–	27767	–	–	28	38	CP002238
	total sequenced bp	total 5' missing	total 3' missing	approx. total length[i]	total ORFs	–	total sequenced bp	total 5' missing	total 3' missing	approx. total length[i]	total plasmid ORFs	–
linear plasmids	362778	21	7	362806	363	–	250549	2006	4120	285675	232	–
circular plasmids	249330	–	–	279330	329	–	186812	–	–	186812	241	–
all plasmids	612108	–	–	612108	692	–	437361	–	–	472487	473	–
whole genome	1522832	21	7	1552860	1517	–	1339552	2261	4220	1375028	1287	–

	JD1						297					
	sequence (bp)	approximate missing bp 5'	3'	Measured size (kbp)[b]	annotated ORFs	accession No.	sequence (bp)	approximate missing bp 5'	3'	Measured size (kbp)[b]	annotated ORFs	accession No.
chromosome	922801	30	600	–	829	CP002312	nd[f]	–	–	921000[h]	–	–
lp5	–	–	–	–	–	–	–	–	–	–	–	–
lp17	17522	150	700	19	15	CP002313	12965	2900	3000	19	11	CP002264
lp21	–	–	–	–	–	–	–	–	–	–	–	–
lp25	22827	1000	500	24.5	15	CP002308	e	–	–	24	–	–
lp28-1	24534	nd	nd	nd	13	CP002306	20978	500	4000	26	11	CP002265
lp28-2	–	–	–	–	–	–	–	–	–	–	–	–
lp28-3	28797	0[d]	0[d]	29	22	CP002314	25211	2200	1800	29.5	17	CP002253
lp28-4	30096	2200	200	31.5	26	CP002318	25788	2100	2600	30	21	CP002266
lp28-5	24610	2000	1000	29	28	CP00231 7	20974	5000	2700	28	25	CP002267
lp28-6	26991	0[d]	300	28	30	CP002324	22135	2200	2600	27	24	CP002259
lp28-7	29993	1000	300	30.5	33	CP002319	–	–	–	–	–	–
lp36	22808	900	400	24	21	CP002315	22715	1300	100	24	21	CP002256
lp38	27590	100	1300	29	23	CP002325	27035	2000	2500	31	28	CP002258
lp54	52926	17[a]	9[a]	54	64	CP001652	48220	3000	4000	54	60	CP001653
lp56	–	–	–	–	–	–	–	–	–	–	–	–
cp9-1	–	–	–	–	–	–	–	–	–	–	–	–
cp26	26525	–	–	26	26	CP002316	26498	–	–	26	26	CP002268
cp32-1	60739	–	–	62	85	CP002310	30920	–	–	31	42	CP002254
cp32-3	29707	–	–	30	41	CP002320	30262	–	–	31	42	CP002269
cp32-4	–	–	–	–	–	–	30301	–	–	30	42	CP002270
cp32-5	(fused to cp32-1)	–	–	–	–	–	30636	–	–	31	42	CP002261
cp32-6	30804	–	–	31	42	CP002307	30641	–	–	31	42	CP002263
cp32-7	–	–	–	–	–	–	21165	–	–	21	29	CP002257
cp32-8	31085	–	–	31	42	CP002311	–	–	–	–	–	–
cp32-9	30697	–	–	31	43	CP002322	21172	–	–	21	29	CP002262
cp32-10	30314	–	–	30	42	CP002323	–	–	–	–	–	–
cp32-11	29902	–	–	30	41	CP002309	30286	–	–	30	42	CP002260
cp32-12	30019	–	–	30	41	CP002321	30795	–	–	31	42	CP002255
	total sequenced bp	total 5' missing	total 3' missing	approx. total length[i]	total plasmid ORFs	–	total sequenced bp	total 5' missing	total 3' missing	approx. total length[i]	total plasmid ORFs	–
linear plasmids	308694	7368	4709	320771	290	–	226021	21200	23300	294521	218	–
circular plasmids	299792	–	–	299792	403	–	282676	–	–	282676	378	–
all plasmids	608486	–	–	620563	693	–	508697	–	–	577197	596	–
whole genome	1531287	7398	5309	1543994	1522	–	508697	–	–	1498197	1416[h]	–

Table S1 footnotes

a. Values include terminal bp determined by Fraser *et al.* [1], Zhang *et al.* [2], Huang *et al.* [3], and Tourand *et al.* [4]. We (J. Aron, S. Casjens and W. M. Huang, unpublished) independently determined the sequence to the tips of the B31 lp54 plasmid. Since the lp54s are all extremely similar, these values assume the tips are all the same. B31 lp28-1 length was assembled from several published sequences as described in text.
b. All plasmid sizes were measured by Southern analysis, and the values obtained agree with the size of the sequence plus the missing sequence to within experimental error.
c. The lp5 ends are virtually identical to those of lp21. If this relationship continues to the ends, there are 21 and 7 bp that were not determined experimentally at the left and right end of lp5, respectively.
d. Bulk sequence went to the end of the telomere of plasmid; this be discussed in detail in a subsequent publication (S. Casjens, D. Radune and W. M. Huang, unpublished).
e. Present in some cultures of this strain but lost from sequenced culture (see text).
f. nd, not determined
g. cp32-10 is integrated into the lp56 plasmid in strain B31 [5].
h. Assumes same chromosome constant region as is present in other strains and includes right end analysis of Huang *et al.* [3].
i. Includes approximate lengths of plasmids known to be present in the strain but which were not sequenced (see footnote e).

References

1. Fraser CM, Casjens S, Huang WM, Sutton GG, Clayton R, *et al.* (1997) Genomic sequence of a Lyme disease spirochaete, *Borrelia burgdorferi*. Nature 390: 580-586.
2. Zhang JR, Hardham JM, Barbour AG, Norris SJ (1997) Antigenic variation in Lyme disease *borreliae* by promiscuous recombination of VMP-like sequence cassettes. Cell 89: 275-285.
3. Huang WM, Robertson M, Aron J, Casjens S (2004) Telomere exchange between linear replicons of *Borrelia burgdorferi*. J Bacteriol 186: 4134-4141.
4. Tourand Y, Deneke J, Moriarty TJ, Chaconas G (2009) Characterization and in vitro reaction properties of 19 unique hairpin telomeres from the linear plasmids of the lyme disease spirochete. J Biol Chem 284: 7264-7272.
5. Casjens S, Palmer N, van Vugt R, Huang WM, Stevenson B, *et al.* (2000) A bacterial genome in flux: the twelve linear and nine circular extrachromosomal DNAs in an infectious isolate of the Lyme disease spirochete *Borrelia burgdorferi*. Mol Microbiol 35: 490-516.
