# Supplementary material for: Genome Stability of Lyme Disease Spirochetes: Comparative Genomics of Borrelia burgdorferi Plasmids
Source: PLoS One. 2012 Mar 14;7(3):e33280. doi: 10.1371/journal.pone.0033280 (PMC3303823; doi:10.1371/journal.pone.0033280)
Supplement: Table S2 — Paralogous protein families in four B. burgdorferi genomes. (PDF) [file pone.0033280.s009.pdf]

Table S2

Paralogous protein families in four *B. burgdorferi* genomes

Table S2 legend

A global analysis was performed using BLAST [6] to accumulate a list of proteins which have paralogs in at least one of the four strains analyzed (see Materials and Methods of text of paper for details). These protein families were named according to the paralogous family (PFam) names used for strain B31 by Casjens *et al.* [5]. Because of the re-annotation of the B31 genomes that did not include genes shorter than 50 codons, some of the previously named PFams do not appear in the current analysis. The PFam names do not include all integers (there are 160 PFams but the names go up to 194). Genes are named here by GenBank locus_tags, so strain B31 names begin with "bb" instead of "*b31_*" as in the text of the article, and the initial "Bbu" of the 297, N40 and JD1 locus tags are present although they were not used in the text.

Within each PFam in Table S2, the genes encoding the proteins on any line are orthologous in the sense that they lie on plasmids in the *same putative compatibility group* (PFam32 types, see text) and in regions of synteny. Genes on different lines are lie on plasmids of different compatibility (PFam32) type or that are not syntenic with the other homologs; *i.e.*, homologous proteins that are encoded on different plasmid types, even if they have *local* synteny, are *not* listed on the same line. Except for the partition proteins, proteins from JD1 plasmid cp32-1+5 cannot be correlated with either of the two PFam32 proteins encoded by this plasmid and so their orthology is not known. B31 genes from the cp32-10 that is integrated into lp56 are considered to be orthologous with other cp32-10s.

A dash (–) in the table indicates that an orthologous gene (by the above definition) is not present in our sequence data. In a given case this could mean that it is present in the strain but was not sequenced (true for any gene in the constant region of the strain 297 chromosome; lp28-4 in N40 and lp25 in 297; see text), is actually not present in the strain (likely true for the B31, JD1 and N40 columns, although a few telomere proximal genes could have been missed by the sequencing, but less likely in the 297 linear plasmids, since several kbp was not sequenced from a number of their termini). Asterisks (*) denote genes that appear to be disrupted by virtue of an internal stop codon, frameshift or truncation compared to other homologs (this does not necessarily imply that these are not expressed or have no function).

The comments column notes functions, predicted functions, other names, *etc.* References for these are too numerous to list here, but see the following references for global analyses and studies of surface proteins and immunogenic proteins [1-5].

References

1. Barbour AG, Jasinskas A, Kayala MA, Davies DH, Steere AC*, et al.* (2008) A genome-wide proteome array reveals a limited set of immunogens in natural infections of humans and white-footed mice with *Borrelia burgdorferi*. Infect Immun 76: 3374-3389.
8. Brooks CS, Vuppala SR, Jett AM, Akins DR (2006) Identification of *Borrelia burgdorferi* outer surface proteins. Infect Immun 74: 296-304.

9. Jacobs JM, Yang X, Luft BJ, Dunn JJ, Camp DG, 2nd, *et al.* (2005) Proteomic analysis of Lyme disease: global protein comparison of three strains of Borrelia burgdorferi. Proteomics 5: 1446-1453.
10. Nowalk AJ, Gilmore RD, Jr., Carroll JA (2006) Serologic proteome analysis of *Borrelia burgdorferi* membrane-associated proteins. Infect Immun 74: 3864-3873.
11. Nowalk AJ, Nolder C, Clifton DR, Carroll JA (2006) Comparative proteome analysis of subcellular fractions from *Borrelia burgdorferi* by NEPHGE and IPG. Proteomics 6: 2121-2134.
12. Skare JT, Foley DM, Hernandez SR, Moore DC, Blanco DR, *et al.* (1999) Cloning and molecular characterization of plasmid-encoded antigens of Borrelia burgdorferi. Infect Immun 67: 4407-4417.

PFam	B31	297	JD1	N40	Comments
1					Restriction/modification proteins
1	BB_0849.2*	–	–	–	truncated
1	BB_E02	–	BbuJD1_E01	BbuN40_E01	full length; JD1_E01 extends out of known sequence
1	BB_H09	Bbu297_H03	BbuJD1_H09	–	full length
1	BB_H11.1*	–	–	–	truncated
1	BB_K02.1*	–	–	–	truncated
1	BB_K10*	Bbu297_K06*	BbuJD1_K06*	BbuN40_K06*	truncated
1	BB_K55*	–	–	BbuN40_K22*	truncated
1	BB_K56*	–	–	BbuN40_K25*	truncated
1	–	Bbu297_I30*	BbuJD1_I36*	BbuN40_I31*	N-terminally truncated
1	–	Bbu297_J01*	BbuJD1_J01*	–	C-terminally truncated
1	–	Bbu297_Y05*	BbuJD1_Y13*	BbuN40_Y04	297 and JD1 truncated; N40 full length
1	–	Bbu297_Y09	BbuJD1_Y16	–	full length
1	–	–	BbuJD1_0896*	–	truncated
1	–	–	BbuJD1_0905	–	full length; locally syntenic with N40_Y04
1	–	–	BbuJD1_AA03*	–	N-terminally truncated
2	BB_0246	–	BbuJD1_0246	BbuN40_0246	Predicted peptidases
2	BB_0255	–	BbuJD1_0255	BbuN40_0255	
2	BB_0262	–	BbuJD1_0262	BbuN40_0262	
2	BB_0761	–	BbuJD1_0761	BbuN40_0761	
3	BB_0611	–	BbuJD1_0611	BbuN40_0611	Predicted ClpP proteases
3	BB_0757	–	BbuJD1_0757	BbuN40_0757	

PFam	B31	297	JD1	N40	Comments
4	BB_0080	–	BbuJD1_0080	BbuN40_0080	Predicted ABC transporters
4	BB_0146	–	BbuJD1_0146	BbuN40_0146	
4	BB_0218	–	BbuJD1_0218	BbuN40_0218	
4	BB_0318	–	BbuJD1_0318	BbuN40_0318	
4	BB_0334	–	BbuJD1_0334	BbuN40_0334	
4	BB_0335	–	BbuJD1_0335	BbuN40_0335	
4	BB_0466	–	BbuJD1_0466	BbuN40_0466	
4	BB_0573	–	BbuJD1_0573	BbuN40_0573	
4	BB_0642	–	BbuJD1_0642	BbuN40_0642	
4	BB_0677	–	BbuJD1_0677	BbuN40_0677	
4	BB_0742	–	BbuJD1_0742	BbuN40_0742	
4	BB_0754	–	BbuJD1_0754	BbuN40_0754	
4	BB_0837	–	BbuJD1_0837	BbuN40_0837	
4	BB_J26	Bbu297_J18	–	BbuN40_J16	
6	BB_0020	–	BbuJD1_0020	BbuN40_0020	Predicted phosphofructokinase
6	BB_0727	–	BbuJD1_0727	BbuN40_0727	
8	BB_0302	–	BbuJD1_0302	BbuN40_0302	Predicted cell division proteins
8	BB_0719	–	BbuJD1_0719	BbuN40_0719	
9	BB_0264	–	BbuJD1_0264	BbuN40_0264	
9	BB_0518	–	BbuJD1_0518	BbuN40_0518	0BB_0518 predicted DnaK type chaperone
9	BB_0715	–	BbuJD1_0715	BbuN40_0715	
10	BB_0076	–	BbuJD1_0076	BbuN40_0076	Predicted flagellar biosynthesis proteins
10	BB_0270	–	BbuJD1_0270	BbuN40_0270	
10	BB_0694	–	BbuJD1_0694	BbuN40_0694	

PFam	B31	297	JD1	N40	Comments
11	BB_0088	–	BbuJD1_0088	BbuN40_0088	Predicted translation factors
11	BB_0476	–	BbuJD1_0476	BbuN40_0476	
11	BB_0540	–	BbuJD1_0540	BbuN40_0540	
11	BB_0691	–	BbuJD1_0691	BbuN40_0691	
11	BB_0801	–	BbuJD1_0801	BbuN40_0801	
12	BB_0844	–	BbuJD1_0889	–	predicted to be lipoproteins
12	BB_G01	–	–	BbuN40_G01	
12	BB_H37	Bbu297_H27	BbuJD1_H43	–	
12	BB_J08	–	–	BbuN40_J03*	
12	BB_K01	–	–	–	
12	–	Bbu297_Y07	BbuJD1_Y14	–	
12	–	–	BbuJD1_I47	BbuN40_I32	
13	BB_0578	–	BbuJD1_0578	BbuN40_0578	Predicted methyl-accepting chemotaxis proteins
13	BB_0596	–	BbuJD1_0596	BbuN40_0596	
13	BB_0597	–	BbuJD1_0597	BbuN40_0597	
13	BB_0680	–	BbuJD1_0680	BbuN40_0680	
13	BB_0681	–	BbuJD1_0681	BbuN40_0681	
14					Response regulator proteins
14	BB_0419	–	BbuJD1_0419	BbuN40_0419	Rrp1, a cyclic-di-GMP-producing response regulator
14	BB_0420	–	BbuJD1_0420	BbuN40_0420	
14	BB_0551	–	BbuJD1_0551	BbuN40_0551	
14	BB_0567	–	BbuJD1_0567	BbuN40_0567	
14	BB_0570	–	BbuJD1_0570	BbuN40_0570	
14	BB_0669	–	BbuJD1_0669	BbuN40_0669	
14	BB_0672	–	BbuJD1_0672	BbuN40_0672	
14	BB_0763	–	BbuJD1_0763	BbuN40_0763	Hk2 putative histidine protein kinase
14	BB_0764	–	BbuJD1_0764	BbuN40_0764	Rrp2 response regulator

PFam	B31	297	JD1	N40	Comments
15	BB_0517	–	BbuJD1_0517	BbuN40_0517	Predicted DnaJ type chaperone
15	BB_0602	–	BbuJD1_0602	BbuN40_0602	
15	BB_0655	–	BbuJD1_0655	BbuN40_0655	
16	BB_0116	–	BbuJD1_0116	BbuN40_0116	Predicted sugar transport proteins
16	BB_0645	–	BbuJD1_0645	BbuN40_0645	
16	BB_B29	Bbu297_B029	BbuJD1_B29	BbuN40_B29	
18	BB_0344	–	BbuJD1_0344	BbuN40_0344	Predicted DNA helicases
18	BB_0607	–	BbuJD1_0607	BbuN40_0607	
18	BB_0633	–	BbuJD1_0633	BbuN40_0633	
19	BB_0408	–	BbuJD1_0408	BbuN40_0408	Predicted sugar transport proteins
19	BB_0447	–	BbuJD1_0447	BbuN40_0447	
19	BB_0629	–	BbuJD1_0629	BbuN40_0629	
20	BB_0581	–	BbuJD1_0581	BbuN40_0581	Predicted DNA helicases
20	BB_0623	–	BbuJD1_0623	BbuN40_0623	
21	BB_0002	–	BbuJD1_0002	BbuN40_0002	
21	BB_0620	–	BbuJD1_0620	BbuN40_0620	
22	BB_0253	–	BbuJD1_0253	BbuN40_0253	
22	BB_0613	–	BbuJD1_0613	BbuN40_0613	
23	BB_0369	–	BbuJD1_0369	BbuN40_0369	
23	BB_0834	–	BbuJD1_0834	BbuN40_0834	
25	BB_0137	–	BbuJD1_0137	BbuN40_0137	
25	BB_0593	–	BbuJD1_0593	BbuN40_0593	
26					MetK/Bgp - putative nucleotidase and glucosaminoglycan binding
26	BB_0375	–	BbuJD1_0375	BbuN40_0375	
26	BB_0588	–	BbuJD1_0588	BbuN40_0588	
26	BB_E07*	–	–	–	
26	BB_I06	Bbu297_I05	BbuJD1_I04	BbuN40_I09	

PFam	B31	297	JD1	N40	Comments
29	BB_0451	–	BbuJD1_0451	BbuN40_0451	Predicted glycosyl hydrolases
29	BB_0452	–	BbuJD1_0452	BbuN40_0452	
30	BB_0036	–	BbuJD1_0036	BbuN40_0036	Predicted DNA topoisomerase subunits
30	BB_0436	–	BbuJD1_0436	BbuN40_0436	
31	BB_0035	–	BbuJD1_0035	BbuN40_0035	Predicted DNA topoisomerase subunits
31	BB_0435	–	BbuJD1_0435	BbuN40_0435	
32					Homology to *parA* genes in other bacterial systems and shown to function in plasmid partitioning in *Borrelia.* Previously called "Orf-C".
32	BB_0269	–	BbuJD1_0269	BbuN40_0269	
32	BB_0361	–	BbuJD1_0361	BbuN40_0361	
32	BB_0431	–	BbuJD1_0431	BbuN40_0431	
32	BB_0726	–	BbuJD1_0726	BbuN40_0726	
32	BB_0843.1*	–	–	–	
32	BB_A20	Bbu297_A20	BbuJD1_A20	BbuN40_A20	
32	BB_B12	Bbu297_B12	BbuJD1_B12	BbuN40_B12	
32	BB_D21	Bbu297_D19	BbuJD1_D17	BbuN40_D29	
32	BB_E19	–	BbuJD1_E09	BbuN40_E08	
32	BB_F11.1*	–	–	–	
32	BB_F13	–	–	–	
32	BB_F24	Bbu297_F18	BbuJD1_F14	–	
32	BB_G08	–	–	BbuN40_G08	
32	BB_H28	Bbu297_H22	BbuJD1_H34	–	
32	BB_I21	Bbu297_I17	BbuJD1_I15	BbuN40_I18	
32	BB_J17	Bbu297_J24	BbuJD1_J11	BbuN40_J08	

32 continued on next page

PFam	B31	297	JD1	N40	Comments
32	BB_K21	Bbu297_K16	BbuJD1_K18	BbuN40_K16	
32	BB_L32	–	BbuJD1_L32	–	
32	BB_M32	Bbu297_M32	BbuJD1_M32	–	
32	BB_N32	Bbu297_N28	BbuJD1_N34	BbuN40_N37	
32	BB_O32	Bbu297_O20	–	BbuN40_O15	
32	BB_P32	Bbu297_P32	BbuJD1_PV32	–	
32	BB_Q08	–	–	–	
32	BB_Q40	–	BbuJD1_Q34	BbuN40_Q34	
32	BB_R33	Bbu297_R34	–	BbuN40_R10	
32	BB_S35	Bbu297_S34	BbuJD1_S32	–	
32	BB_U05	–	–	–	
32	–	Bbu297_V32	BbuJD1_PV74	BbuN40_V31	
32	–	Bbu297_W37	BbuJD1_W35	–	
32	–	Bbu297_X32	BbuJD1_X35	BbuN40_X30	
32	–	Bbu297_Y12	BbuJD1_Y03	BbuN40_Y09	N40_Y09 in inversion
32	–	Bbu297_Z02	BbuJD1_Z04	–	
32	–	–	BbuJD1_0902	–	
32	–	–	BbuJD1_AA06	–	
33	BB_0040	–	BbuJD1_0040	BbuN40_0040	Predicted chemotaxis proteins
33	BB_0312	–	BbuJD1_0312	BbuN40_0312	
33	BB_0414	–	BbuJD1_0414	BbuN40_0414	
33	BB_0565	–	BbuJD1_0565	BbuN40_0565	
33	BB_0670	–	BbuJD1_0670	BbuN40_0670	
34	BB_0251	–	BbuJD1_0251	BbuN40_0251	Predicted tRNA synthases
34	BB_0587	–	BbuJD1_0587	BbuN40_0587	
34	BB_0738	–	BbuJD1_0738	BbuN40_0738	
34	BB_0833	–	BbuJD1_0833	BbuN40_0833	

PFam	B31	297	JD1	N40	Comments
35	BB_0405	–	BbuJD1_0405	BbuN40_0405	
35	BB_0406	–	BbuJD1_0406	BbuN40_0406	
35	BB_0562	–	BbuJD1_0562	BbuN40_0562	
35	BB_0563	–	BbuJD1_0563	BbuN40_0563	
35	BB_0564	–	BbuJD1_0564	BbuN40_0564	
36	BB_0382	–	BbuJD1_0382	BbuN40_0382	BmpB; immunogenic surface protein, binds plasminogen
36	BB_0383	–	BbuJD1_0383	BbuN40_0383	BmpA; immunogenic surface protein
36	BB_0384	–	BbuJD1_0384	BbuN40_0384	BmpC; immunogenic surface protein
36	BB_0385	–	BbuJD1_0385	BbuN40_0385	BmpD; immunogenic surface protein
37					ABC oligopeptide transporter components
37	BB_0328	–	BbuJD1_0328	BbuN40_0328	OppAI
37	BB_0329	–	BbuJD1_0329	BbuN40_0329	OppAII
37	BB_0330	–	BbuJD1_0330	BbuN40_0330	OppAIII
37	BB_A34	Bbu297_A34	BbuJD1_A34	BbuN40_A34	OppAV; B31 A34 antigenic
37	BB_B16	Bbu297_B16	BbuJD1_B16	BbuN40_B16	OppAIV; B31 B16 antigenic
38	BB_0221	–	BbuJD1_0221	BbuN40_0221	Predicted flagellar motor protein
38	BB_0290	–	BbuJD1_0290	BbuN40_0290	
39	BB_0093	–	BbuJD1_0093	BbuN40_0093	
39	BB_0094	–	BbuJD1_0094	BbuN40_0094	
39	BB_0230	–	BbuJD1_0230	BbuN40_0230	
39	BB_0288	–	BbuJD1_0288	BbuN40_0288	
40	BB_0223	–	BbuJD1_0223	BbuN40_0223	
40	BB_0224	–	BbuJD1_0224	BbuN40_0224	
40	BB_K13	Bbu297_K09*	BbuJD1_K09	BbuN40_K08	

PFam	B31	297	JD1	N40	Comments
41	BB_0145	–	BbuJD1_0145	BbuN40_0145	Predicted ABC transporter proteins
41	BB_0216	–	BbuJD1_0216	BbuN40_0216	
41	BB_0217	–	BbuJD1_0217	BbuN40_0217	
41	BB_0332	–	BbuJD1_0332	BbuN40_0332	
41	BB_0333	–	BbuJD1_0333	BbuN40_0333	
41	BB_0640	–	BbuJD1_0640	BbuN40_0640	
41	BB_0641	–	BbuJD1_0641	BbuN40_0641	
41	BB_0746	–	BbuJD1_0746	BbuN40_0746	
41	BB_0747	–	BbuJD1_0747	BbuN40_0747	
42	BB_0059	–	BbuJD1_0059	BbuN40_0059	
42	BB_0202	–	BbuJD1_0202	BbuN40_0202	
43	BB_0074	–	BbuJD1_0074	BbuN40_0074	peptide release factor; _0074's have programmed translational frameshift
43	BB_0196	–	BbuJD1_0196	BbuN40_0196	
44	BB_0158	–	BbuJD1_0158	BbuN40_0158	
44	BB_0159	–	BbuJD1_0159	BbuN40_0159	
44	BB_A04	Bbu297_A04	BbuJD1_A04	BbuN40_A04	B31 A04 is S2 antigen
44	BB_E09	–	–	BbuN40_E03	B31 E09 is antigenic
44	BB_F22*	–	–	–	
44	BB_H36.1*	–	–	–	
44	BB_K52	Bbu297_K31*	BbuJD1_K36	–	P23 in B31; antigenic; predicted lipoprotein
44	BB_Q04*	–	–	–	B31 Q04 is antigenic
44	–	Bbu297_J05*	BbuJD1_J04	–	
44	–	Bbu297_Y02	BbuJD1_Y11	BbuN40_Y03	
44	–	–	BbuJD1_0908	–	
44	–	–	BbuJD1_0911	–	
45	BB_0018	–	BbuJD1_0018	BbuN40_0018	Predicted rRNA modification enzyme
45	BB_0815*	–	BbuJD1_0815	BbuN40_0815	

PFam	B31	297	JD1	N40	Comments
46					predicted helicases
46	BB_0111	–	BbuJD1_0111	BbuN40_0111	
46	BB_G32	–	–	BbuN40_G30	
46	–	–	BbuJD1_AA34	–	region of local synteny with B31_G32
46	–	–	–	BbuN40_Y14	
47	BB_0050	–	BbuJD1_0050	BbuN40_0050	
47	BB_0051	–	BbuJD1_0051	BbuN40_0051	
					Outer membrane channel forming protein
48	BB_0034	–	BbuJD1_0034	BbuN40_0034	protein P13; outer membrane channel forming protein
48	BB_A01	–	BbuJD1_A01	BbuN40_A01	surface protein; outer membrane channel forming protein
48	BB_G03*	–	–	BbuN40_G03	
48	BB_H41	–	–	–	
48	BB_I31*	Bbu297_I24	BbuJD1_I24	BbuN40_I25	
48	BB_Q06	–	–	–	
48	BB_Q81*	–	–	–	
48	–	–	BbuJD1_AA04*	–	
48	–	–	BbuJD1_E26*	–	local synteny with B31 lp28-2
49					Implicated in plasmid partitioning/replication by gene positions; Previously called "Orf-3"
49	BB_A21	Bbu297_A21	BbuJD1_A21	BbuN40_A21	
49	BB_B13	Bbu297_B013	BbuJD1_B13	BbuN40_B13	
49	BB_C03	–	–	BbuN40_C03	
49	BB_E18	–	BbuJD1_E08	BbuN40_E07	
49	BB_F12*	–	–	–	

49 continued on next page

PFam	B31	297	JD1	N40	Comments
49	BB_F23	Bbu297_F17	BbuJD1_F13	–	
49	BB_G09	–	–	BbuN40_G09	
49	BB_H29	Bbu297_H23	BbuJD1_H35	–	
49	BB_I22	Bbu297_I18	BbuJD1_I16	BbuN40_I19	
49	BB_I40*	Bbu297_I29*	BbuJD1_I32*	BbuN40_I30*	
49	BB_J16	Bbu297_J25	BbuJD1_J12	BbuN40_J07	
49	BB_K24	Bbu297_K19	BbuJD1_K21	BbuN40_K19	
49	BB_L34	–	BbuJD1_L33	–	
49	BB_M33	Bbu297_M33	BbuJD1_M33	–	
49	BB_N33	Bbu297_N29	BbuJD1_N35	BbuN40_N38	
49	BB_O33	Bbu297_O21	–	BbuN40_O16	
49	BB_P33	–	–	–	
49	BB_Q07	–	–	–	
49	BB_Q41	–	BbuJD1_Q35	BbuN40_Q35	
49	BB_R34	Bbu297_R35	–	BbuN40_R11	
49	BB_S36	Bbu297_S35	BbuJD1_S33	–	
49	BB_U06	–	–	–	
49	–	Bbu297_J13*	–	–	
49	–	Bbu297_P33	–	–	
49	–	Bbu297_V33	–	BbuN40_V32	
49	–	Bbu297_W38	BbuJD1_W36	–	
49	–	Bbu297_X33	BbuJD1_X37	BbuN40_X31	
49	–	Bbu297_Y13	BbuJD1_Y04	BbuN40_Y08	BbuN40_Y08 in inversion
49	–	Bbu297_Z01	BbuJD1_Z03	–	
49	–	–	BbuJD1_0903	–	
49	–	–	BbuJD1_AA05	–	
49	–	–	BbuJD1_J19	–	local synteny with section of B31 lp21
49	–	–	BbuJD1_PV33	–	
49	–	–	BbuJD1_PV75	–	

PFam	B31	297	JD1	N40	Comments
50					Implicated in plasmid partitioning/replication by gene positions. Previously called "Orf-2".
50	BB_A19	Bbu297_A19	BbuJD1_A19	BbuN40_A19	Plasmid partitioning/replication
50	BB_B11	Bbu297_B011	BbuJD1_B11	BbuN40_B11	
50	BB_C02	–	–	BbuN40_C02	
50	BB_E20	–	BbuJD1_E10	BbuN40_E09	
50	BB_F0040	–	–	–	
50	BB_F14	–	–	–	
50	BB_F25	Bbu297_F19	BbuJD1_F15	–	
50	BB_G07	–	–	BbuN40_G07	
50	BB_G31*	–	–	BbuN40_G29	
50	BB_H07*	–	–	–	
50	BB_H27	Bbu297_H21	BbuJD1_H33*	–	
50	BB_I20	Bbu297_I16	BbuJD1_I14	BbuN40_I17	
50	BB_J18	Bbu297_J23	BbuJD1_J10	BbuN40_J09	
50	BB_K22	Bbu297_K17	BbuJD1_K19	BbuN40_K17	
50	BB_L31	–	BbuJD1_L31	–	
50	BB_M31	Bbu297_M31	BbuJD1_M31	–	
50	BB_N31	Bbu297_N27	BbuJD1_N33	BbuN40_N36	
50	BB_O31	Bbu297_O19	–	BbuN40_O14	
50	BB_P31	Bbu297_P31	–	–	
50	BB_Q09	–	–	–	
50	BB_Q39	–	BbuJD1_Q33	BbuN40_Q33	
50	BB_R32	Bbu297_R33	–	BbuN40_R09	
50	BB_S34	Bbu297_S33	BbuJD1_S31	–	
50	–	Bbu297_J14*	–	–	
50	–	Bbu297_V31	–	BbuN40_V30	

50 continued on next page

PFam	B31	297	JD1	N40	Comments
50	–	Bbu297_W36	BbuJD1_W34	–	
50	–	Bbu297_X31	BbuJD1_X34	BbuN40_X29	
50	–	Bbu297_Y11	BbuJD1_Y02	BbuN40_Y10	
50	–	Bbu297_Z03	BbuJD1_Z05	–	
50	–	–	BbuJD1_0901	–	
50	–	–	BbuJD1_AA07	–	
50	–	–	BbuJD1_AA33	–	
50	–	–	BbuJD1_PV31	–	
50	–	–	BbuJD1_PV73	–	
50	–	–	BbuJD1_Z32	–	
52	BB_I42	–	–	–	B31 I42 is surface protein, antigenic, predicted lipoprotein
52	BB_J50*	–	–	–	
52	BB_K53	Bbu297_K32	BbuJD1_K37	–	B31 K53 antigenic, predicted lipoprotein
52	BB_Q03	–	–	–	B31 Q03 is antigenic, predicted lipoprotein
52	BB_T07*	–	–	–	
52	BB_U12*	–	–	–	
52	–	Bbu297_J11*	–	BbuN40_J33*	
52	–	Bbu297_J12*	–	–	
52	–	–	BbuJD1_0912	–	
52	–	–	BbuJD1_E04	–	
52	–	–	BbuJD1_H47	–	
52	–	–	BbuJD1_J21*	–	in region of local synteny with B31 lp21
52	–	–	BbuJD1_J25*	–	In region of local synteny with B31 lp21
52	–	–	–	BbuN40_Y01	
53	BB_A15	Bbu297_A15	BbuJD1_A15	BbuN40_A15	outer surface lipoprotein OspA
53	BB_A16	Bbu297_A16*	BbuJD1_A16	BbuN40_A16	outer surface lipoprotein OspB

PFam	B31	297	JD1	N40	Comments
54	BB_A64	Bbu297_A64	BbuJD1_A64	BbuN40_A64	antigenic; elicits protective immunity in mice; surface protein; predicted lipoprotein
54	BB_A65	Bbu297_A65	BbuJD1_A65	BbuN40_A65	predicted lipoprotein
54	BB_A66	Bbu297_A66	BbuJD1_A66	BbuN40_A66	surface protein, antigenic, predicted lipoprotein
54	BB_A68	Bbu297_A68	BbuJD1_A68	BbuN40_A68	complement regulator-acquiring surface protein 1 (CRASP-1); surface protein; predicted lipoprotein
54	BB_A69	Bbu297_A69	BbuJD1_A69	BbuN40_A69	surface protein; predicted lipoprotein
54	BB_A70*	–	–	–	
54	BB_A71*	Bbu297_A71	BbuJD1_A71	BbuN40_A71	truncated
54	BB_A73	–	BbuJD1_A73	BbuN40_A73	surface protein; predicted lipoprotein
54	BB_I36	Bbu297_I27	BbuJD1_I30	BbuN40_I28	predicted lipoprotein
54	BB_I38	Bbu297_I28	BbuJD1_I31	BbuN40_I29	B31 I38 is surface protein, predicted lipoprotein
54	BB_I39	–	–	–	B31 I39 surface protein, predicted lipoprotein
54	BB_J41	–	–	–	B31 J41 is surface protein, predicted lipoprotein
54	BB_J42	–	–	–	
54	–	Bbu297_A67.5	–	BbuN40_A67.5	
54	–	Bbu297_Y16	BbuJD1_Y08	–	

PFam	B31	297	JD1	N40	Comments
55	BB_C08	–	–	BbuN40_C11*	
55	BB_I43*	–	–	–	
55	BB_K54*	–	–	BbuN40_K21*	
55	BB_Q01*	–	–	–	
55	BB_U09	–	–	–	
55	–	Bbu297_W45	–	–	
55	–	–	BbuJD1_J23*	–	In region of local synteny with B31 lp21
55	–	–	BbuJD1_PV40	–	
56	BB_0473	–	BbuJD1_0473	BbuN40_0473	
56	BB_0583	–	BbuJD1_0583	BbuN40_0583	
56	BB_0584	–	BbuJD1_0584	BbuN40_0584	
57					Plasmid partitioning, distant relatives of PFam62; previously called "Orf-1"
57	BB_0849.1*	–	–	–	
57	BB_0853*	–	–	–	
57	BB_A18	Bbu297_A18	BbuJD1_A18	BbuN40_A18	
57	BB_C01	–	–	BbuN40_C01	
57	BB_D03*	–	–	–	
57	BB_D05.1*	–	–	BbuN40_D09*	
57	BB_E21	–	BbuJD1_E11	BbuN40_E10	
57	BB_E33*	–	–	–	
57	BB_F05*	–	–	–	
57	BB_F06*	–	–	–	
57	BB_F26	Bbu297_F20	BbuJD1_F16	–	
57	BB_G06	–	–	BbuN40_G06	
57	BB_H04*	–	BbuJD1_H04*	–	
57	BB_H05*	–	BbuJD1_H05*	–	
57	BB_I01*				

57 continued on next page

PFam	B31	297	JD1	N40	Comments
57	BB_I02.2*	Bbu297_I02*	BbuJD1_I43*	BbuN40_I06*	
57	BB_I19	Bbu297_I15	BbuJD1_I13	BbuN40_I16	
57	BB_L30	–	BbuJD1_L30	–	
57	BB_M30	Bbu297_M30	BbuJD1_M30	–	
57	BB_N30	Bbu297_N26	BbuJD1_N32	BbuN40_N35	
57	BB_O30	Bbu297_O18	–	BbuN40_O13	
57	BB_P30	Bbu297_P30	–	–	
57	BB_Q38	–	BbuJD1_Q32	BbuN40_Q32	
57	BB_Q84.1*	–	–	–	
57	BB_Q85*	–	–	–	
57	BB_R31	Bbu297_R32	–	BbuN40_R08	
57	BB_S33	Bbu297_S32	BbuJD1_S30	–	
57	BB_T0008	–	–	–	
57	BB_T01*	–	–	–	
57	BB_U01*	–	–	–	
57	BB_U04	–	–	–	
57	BB_U07*	–	–	–	
57	BB_U10*	–	–	–	
57	–	Bbu297_F37*	BbuJD1_F32*	–	
57	–	Bbu297_I01	BbuJD1_I41	BbuN40_I04	
57	–	Bbu297_V30	–	BbuN40_V29	
57	–	Bbu297_W35	BbuJD1_W33	–	
57	–	Bbu297_X30	BbuJD1_X33	BbuN40_X28	
57	–	Bbu297_Y10	BbuJD1_Y01	BbuN40_Y11	in inverted section
57	–	Bbu297_Y17*	–	–	
57	–	–	BbuJD1_0899	–	
57	–	–	BbuJD1_I40*	BbuN40_I03*	
57	–	–	BbuJD1_J20*	–	in region syntenic with B31 lp21

57 continued on next page

PFam	B31	297	JD1	N40	Comments
57	–	–	BbuJD1_PV30*	–	
57	–	–	BbuJD1_PV72	–	
57	–	–	BbuJD1_Y09*	–	
57	–	–	BbuJD1_Y18*	–	
57	–	–	–	BbuN40_K01*	
59	BB_I08.1*	Bbu297_I07*	BbuJD1_I07*	BbuN40_I10*	
59	BB_J31	–	–	BbuN40_J20	
59	BB_J45	–	–	BbuN40_J27	
59	BB_K07	Bbu297_K03	BbuJD1_K03	BbuN40_K04*	B31 K07 is antigenic, predicted lipoprotein
59	BB_K12	Bbu297_K07*	BbuJD1_K08*	BbuN40_K07	
59	BB_K39*	–	–	–	
60					Intact members predicted to be lipoproteins
60	BB_D0027*	–	–	BbuN40_D10	
60	BB_E31	–	BbuJD1_E21	BbuN40_E31	B31 E31 is surface protein
60	BB_H32	Bbu297_H24	BbuJD1_H38	–	
60	BB_I15*	Bbu297_I12*	BbuJD1_I10*	BbuN40_I13*	
60	BB_I16	Bbu297_I13	–	–	B31 I16 is antigenic surface protein
60	BB_I28*	Bbu297_I22	BbuJD1_I22*	BbuN40_I23	
60	BB_I29	Bbu297_I23	BbuJD1_I23	BbuN40_I24	
60	BB_I34	Bbu297_I26	BbuJD1_I46*	BbuN40_I27	
60	BB_K15	Bbu297_K11*	BbuJD1_K13*	BbuN40_K10*	
60	BB_Q05	–	–	–	
60	BB_Q74*	–	–	–	
60	BB_Q80*	–	–	–	
60	–	Bbu297_J06	BbuJD1_J05	–	
60	–	Bbu297_Y14	BbuJD1_Y05	–	

60 continued on next page

PFam	B31	297	JD1	N40	Comments
60	–	–	BbuJD1_D01*	–	
60	–	–	BbuJD1_I11	BbuN40_I14	
61					Adenine deaminase
61	BB_H33*	Bbu297_H25*	BbuJD1_H39*	–	
61	BB_K17	Bbu297_K13	BbuJD1_K15	BbuN40_K13	
61	–	Bbu297_D01*	BbuJD1_D02*	–	
62					Plasmid partitioning/replication, distant relatives of PFam57
62	BB_0853.1*	–	–	–	
62	BB_B10	Bbu297_B010	BbuJD1_B10	BbuN40_B10	
62	BB_D14	Bbu297_D12	BbuJD1_D10	BbuN40_D20	
62	BB_G29	–	–	BbuN40_G27	
62	BB_H26	Bbu297_H20	BbuJD1_H32	–	
62	BB_H34*	Bbu297_H26*	BbuJD1_H40*	–	
62	BB_J19	Bbu297_J22	BbuJD1_J09	BbuN40_J10	
62	BB_K23	Bbu297_K18	BbuJD1_K20	BbuN40_K18	
62	BB_Q55	–	–	–	
62	–	Bbu297_Z04	BbuJD1_Z06	–	
62	–	Bbu297_Z27	BbuJD1_Z28	–	
62	–	–	BbuJD1_0900	–	
62	–	–	BbuJD1_AA30	–	
62	–	–	–	BbuN40_Y13	
63	BB_C10	–	–	–	RevB; antigenic outer membrane protein; binds fibronectin
63	BB_M27	–	–	–	B31 RevA1/M27 antigenic
63	BB_P27	–	–	–	B31 RevA2/P27 surface protein
63	–	Bbu297_R28	–	–	
63	–	Bbu297_X27	–	–	
63	–	–	–	BbuN40_Q28	

PFam	B31	297	JD1	N40	Comments
64	BB_F16*	–	–	–	
64	BB_K34	Bbu297_K24	BbuJD1_K27	BbuN40_K32	
65					ThyX tymidylate synthase
65	BB_A76	–	BbuJD1_A76	BbuN40_A76	
65	BB_F14.1*	–	–	–	
65	BB_K33*	–	–	BbuN40_K31	
68	BB_F17*	–	–	–	
68	BB_K35*	Bbu297_K25*	BbuJD1_K28*	–	
68	–	–	BbuJD1_Z02	–	
69	BB_H18	Bbu297_H12	BbuJD1_H20*	–	
69	BB_J13*	–	–	BbuN40_J06	
69	BB_K47	–	–	BbuN40_K36*	
69	BB_K49	–	–	–	
69	–	–	–	BbuN40_D03	In region of local synteny to B31_K47
70	BB_F10	–	–	–	
70	BB_K41	–	–	BbuN40_K33	
71	BB_F09*	–	–	–	
71	BB_K42	–	–	BbuN40_K34	
72	BB_F08*	–	–	–	
72	BB_K42.1*	–	–	–	
74					Decorin binding proteins
74	BB_A24	Bbu297_A24	BbuJD1_A24	BbuN40_A24	DbpA; B31 A24 antigenic surface protein
74	BB_A25	Bbu297_A25	BbuJD1_A25	BbuN40_A25	DbpB B31 A25 antigenic surface protein

PFam	B31	297	JD1	N40	Comments
75	BB_K37*	–	–	–	BBK37 is also in family 175 by virtue of similarity at its C-terminus.
75	BB_K45	–	–	–	B31 K45 is antigenic
75	BB_K46*	–	–	–	
75	BB_K48	–	–	–	
75	BB_K50	–	–	–	B31 K50 elicits protective immunity in mice
75	–	–	–	BbuN40_D02	In region of local synteny with B31 lp36
75	–	–	–	BbuN40_D04	In region of local synteny with B31 lp36
75	–	–	–	BbuN40_D05	
76	BB_D001*	–	–	–	
76	BB_D01*	–	–	–	
76	BB_H02	–	BbuJD1_H01	–	
76	BB_Q82*	–	–	–	
76	BB_Q88	–	–	–	
76	BB_Q89	–	–	–	
76	–	–	BbuJD1_I39*	BbuN40_I02*	
78	BB_0283	–	BbuJD1_0283	BbuN40_0283	Predicted flagellar proteins
78	BB_0293	–	BbuJD1_0293	BbuN40_0293	
78	BB_0774	–	BbuJD1_0774	BbuN40_0774	
78	BB_0775	–	BbuJD1_0775	BbuN40_0775	
80					Bdr (Borrelia direct repeat) proteins; antigenic; previously called "Orf-E"
80	BB_F001.1*	–	–	–	
80	BB_F03*	–	–	–	
80	BB_G33	–	–	BbuN40_G31	B31 BdrT
80	BB_H13	Bbu297_H08	BbuJD1_H15	–	B31 BdrU, antigenic
80	BB_J10	–	–	BbuN40_J05	

80 continued on next page

PFam	B31	297	JD1	N40	Comments
80	BB_K40	–	–	–	
80	BB_L27	–	BbuJD1_L27	–	B31 BdrP
80	BB_L35	–	BbuJD1_L34	–	B31 BdrO, antigenic
80	BB_M34	Bbu297_M34	BbuJD1_M34	–	B31 BdrK, antigenic
80	BB_N27	Bbu297_N23	BbuJD1_N28	BbuN40_N30	B31 BdrR, antigenic
80	BB_N34	Bbu297_N30	BbuJD1_N36*	BbuN40_N39	B31 BdrQ, antigenic
80	BB_O27	Bbu297_O15	–	BbuN40_O10	B31 BdrN
80	BB_O34	Bbu297_O23	–	BbuN40_O18	B31 BdrM, antigenic
80	BB_P34	Bbu297_P34	–	–	B31 BdrA, antigenic
80	BB_Q34	–	BbuJD1_Q29	–	B31 BdrW
80	BB_Q42	–	BbuJD1_Q36	BbuN40_Q36	B31 BdrV. Antigenic
80	BB_R27	–	–	BbuN40_R05	B31 BdrH
80	BB_R35*	Bbu297_R36	–	BbuN40_R12	B31 BdrG; antigenic
80	BB_S29	Bbu297_S28	BbuJD1_S27	–	B31 BdrF
80	BB_S37	Bbu297_S36	BbuJD1_S34	–	B31 BdrE
80	–	Bbu297_M27	BbuJD1_M27	–	
80	–	Bbu297_P27	–	–	
80	–	Bbu297_V27	–	BbuN40_V26	
80	–	Bbu297_V34	–	BbuN40_V33	
80	–	Bbu297_W32	BbuJD1_W29	–	
80	–	Bbu297_W39	BbuJD1_W37	–	
80	–	Bbu297_X34	BbuJD1_X38	BbuN40_X32	
80	–	–	BbuJD1_AA35	–	
80	–	–	BbuJD1_PV27	–	
80	–	–	BbuJD1_PV34	–	
80	–	–	BbuJD1_PV69	–	
80	–	–	BbuJD1_PV76	–	
80	–	–	BbuJD1_X28	–	
80	–	–	BbuJD1_Z33	–	

PFam	B31	297	JD1	N40	Comments
82					Putative IS605B type transposase
82	BB_0848.1*	–	–	–	
82	BB_D23*	–	BbuJD1_D20*	BbuN40_D32*	
82	BB_F18*	–	–	–	
82	BB_F19*	–	–	–	
82	BB_G05*	–	–	BbuN40_G05	
82	BB_H40*	–	BbuJD1_H46*	–	
82	BB_I41*	–	–	–	
82	–	Bbu297_J26*	BbuJD1_J14*	–	
82	–	Bbu297_K27*	BbuJD1_K30*	–	
82	–	Bbu297_K30*	BbuJD1_K33*	–	
82	–	–		–	
82	–	–	BbuJD1_E18*	–	
82	–	–	BbuJD1_E24*	–	in region of synteny with B31 lp28-2
82	–	–	BbuJD1_E25*	–	in region of synteny with B31 lp28-2
82	–	–	BbuJD1_J16*	–	
82	–	–	–	BbuN40_Y12	
82	–	–	–	BbuN40_Y15	
84	BB_I0044	–	–	–	
84	BB_T03	–	–	–	
84	BB_U02	–	–	–	
84	–	Bbu297_K01	BbuJD1_K01	BbuN40_K02*	
84	–	–	BbuJD1_Y17	–	
85	BB_D15	Bbu297_D13	BbuJD1_D11	BbuN40_D21	
85	BB_F20*	–	–	–	
85	–	Bbu297_K26*	BbuJD1_K29*	–	

PFam	B31	297	JD1	N40	Comments
86	BB_G22	–	–	BbuN40_G20	
86	BB_G23	–	–	BbuN40_G21	
86	–	Bbu297_Z20	BbuJD1_Z21	–	
86	–	Bbu297_Z21	BbuJD1_Z22	–	
86	–	–	BbuJD1_AA23	–	
86	–	–	BbuJD1_AA24	–	
88	BB_F001*	–	–	–	
88	BB_F02*	–	–	–	
88	BB_G34	–	–	BbuN40_G32	
88	–	–	BbuJD1_AA36	–	
88	–	–	BbuJD1_I38*	–	
88	–	–	BbuJD1_Z34	–	
88	–	–	–	BbuN40_I01*	
88	–	–	–	BbuN40_Y17	
89	BB_0712	–	BbuJD1_0712	BbuN40_0712	Predicted RNA polymerase sigma factors
89	BB_0771	–	BbuJD1_0771	BbuN40_0771	
90	BB_J29	Bbu297_J15	–	BbuN40_J19	
90	BB_J43	–	–	BbuN40_J26	
92	BB_H06	Bbu297_H01	BbuJD1_H06	–	
92	BB_J34	–	–	BbuN40_J22	
92	BB_J36	–	–	BbuN40_J23	
92	–	Bbu297_J27	–	–	
94					Putative guanine transport
94	BB_B22	Bbu297_B022	BbuJD1_B22	BbuN40_B22	
94	BB_B23	Bbu297_B023	BbuJD1_B23	BbuN40_B23	

PFam	B31	297	JD1	N40	Comments
95	BB_C06	–	–	BbuN40_C05	BB_C06 is EppA; exported; antigenic
95	BB_H09.1	Bbu297_H06	BbuJD1_H13	–	
95	BB_S42	–	–	–	BB_S42 is BapA
95	–	Bbu297_M39	–	–	
95	–	–	BbuJD1_AA37	–	
95	–	–	BbuJD1_AA38	–	
95	–	–	BbuJD1_Q41	–	
96					BppC; weak similarity to Erf type phage DNA binding protein; previously called Orf-6"
96	BB_C11	–	–	BbuN40_C12	
96	BB_L38	–	BbuJD1_L37	–	
96	BB_M37	Bbu297_M37	BbuJD1_M37	–	
96	BB_N37*	Bbu297_N34	BbuJD1_N41	BbuN40_N42	
96	BB_O38	Bbu297_O27*	–	BbuN40_O21	
96	BB_P37	Bbu297_P37	–	–	
96	BB_Q45	–	BbuJD1_Q39	BbuN40_Q39	
96	BB_R38	Bbu297_R39	–	BbuN40_R15	
96	BB_S40	Bbu297_S40	BbuJD1_S37	–	
96	–	Bbu297_V37	–	BbuN40_V36	
96	–	Bbu297_W42	BbuJD1_W41	–	
96	–	Bbu297_X37	BbuJD1_X42	BbuN40_X35	
96	–	–	BbuJD1_PV37	–	
96	–	–	BbuJD1_PV79	–	
97	BB_0068	–	BbuJD1_0068	BbuN40_0068	
97	BB_0421	–	BbuJD1_0421	BbuN40_0421	
99	BB_E16	–	BbuJD1_E06	BbuN40_E05	B31 E16 required for tick transmission
99	BB_J47	–	–	BbuN40_J30	

PFam	B31	297	JD1	N40	Comments
101	BB_F26.1*	–	–	–	
101	BB_G10	–	–	BbuN40_G10	
101	BB_Q57	–	–	–	
101	BB_Q59	–	–	–	
101	–	Bbu297_F21	BbuJD1_F35	–	
101	–	Bbu297_F23*	BbuJD1_F20*	–	
101	–	Bbu297_Z06	BbuJD1_Z08	–	
101	–	–	BbuJD1_AA10	–	
102					Predicted adenine specific DNA methyltransferase
102	BB_E29.1*	–	–	–	
102	BB_G02	–	–	BbuN40_G02	
102	BB_H36.2*	–	–	–	
102	BB_Q67	–	–	–	B31 BB_Q67 fused to PFam167 part
102	–	–	BbuJD1_E27	–	in region of synteny with BB_G02 of B31 lp28-2
103	BB_G20	–	–	BbuN40_G17*	
103	BB_Q65*	–	–	–	
103	–	Bbu297_Z16	BbuJD1_Z17	–	
103	–	–	BbuJD1_AA19	–	
104	BB_G24	–	–	BbuN40_G22*	
104	BB_H38*	Bbu297_H18*	BbuJD1_H29*	–	
104	–	Bbu297_Z22	BbuJD1_Z23	–	
104	–	–	BbuJD1_AA25	–	

PFam	B31	297	JD1	N40	Comments
105					Predicted efflix transporter
105	BB_0845.2*	–	–	–	
105	BB_I26	Bbu297_I21	BbuJD1_I19*	BbuN40_I22	
105	BB_Q71*	–	–	–	
105	–	Bbu297_J08*	BbuJD1_J08*	–	
105	–	Bbu297_J09*	–	–	
105	–	Bbu297_Y08*	BbuJD1_Y15*	–	
106	BB_J23	Bbu297_J21	–	BbuN40_J13	
106	BB_J24	Bbu297_J20	–	BbuN40_J14	
106	–	Bbu297_F25	BbuJD1_F23	–	
107	BB_A43	Bbu297_A43	BbuJD1_A43	BbuN40_A43	
107	BB_L08	–	BbuJD1_L08	–	
107	BB_M08	Bbu297_M08	BbuJD1_M08	–	
107	BB_N08	–	BbuJD1_N09	BbuN40_N10	
107	BB_O08	Bbu297_O08	–	–	
107	BB_P08	Bbu297_P08	–	–	
107	BB_Q15	–	BbuJD1_Q08	BbuN40_Q08	
107	BB_R08	Bbu297_R09	–	–	
107	BB_S08	Bbu297_S08	BbuJD1_S08	–	
107	–	Bbu297_V08	–	BbuN40_V08	
107	–	Bbu297_W10	BbuJD1_W09	–	
107	–	Bbu297_X08	BbuJD1_X09	BbuN40_X08	
107	–	–	BbuJD1_PV07	–	
107	–	–	BbuJD1_PV50	–	
108	BB_L09	–	BbuJD1_L09	–	
108	BB_M09	Bbu297_M09	BbuJD1_M09	–	
108	BB_N09	–	BbuJD1_N10	BbuN40_N11	
108	BB_O09	Bbu297_O09*	–	–	

108 continued on next page

PFam	B31	297	JD1	N40	Comments
108	BB_P09	Bbu297_P09	–	–	
108	BB_Q16*	–	BbuJD1_Q09	BbuN40_Q09	
108	BB_R09	Bbu297_R10	–	–	
108	BB_S09	Bbu297_S09	BbuJD1_S09	–	
108	–	Bbu297_V09	–	BbuN40_V09	
108	–	Bbu297_W12	BbuJD1_W10	–	
108	–	Bbu297_X09	BbuJD1_X10	BbuN40_X09	
108	–	–	BbuJD1_PV08	–	
108	–	–	BbuJD1_PV51*	–	
109					BlyA; causes bacterial cell lysis; possible phage holin
109	BB_L23	–	BbuJD1_L23	–	
109	BB_M23	Bbu297_M23	BbuJD1_M23	–	
109	BB_N23	Bbu297_N14	BbuJD1_N24	BbuN40_N26	
109	BB_O23	Bbu297_O11	–	BbuN40_O06	
109	BB_P23	Bbu297_P23	–	–	
109	BB_Q30	–	BbuJD1_Q25	BbuN40_Q24	
109	BB_R23	Bbu297_R24	–	–	
109	BB_S23	Bbu297_S23	BbuJD1_S23	–	
109	–	Bbu297_V23	–	BbuN40_V22	
109	–	Bbu297_W28	BbuJD1_W24	–	
109	–	Bbu297_X23	BbuJD1_X24	BbuN40_X23	
109	–	–	BbuJD1_PV22	–	
109	–	–	BbuJD1_PV65	–	
111	BB_L24	–	BbuJD1_L24	–	
111	BB_M24	Bbu297_M24	BbuJD1_M24	–	
111	BB_N24	Bbu297_N18*	BbuJD1_N25	BbuN40_N27	
111	BB_O24	Bbu297_O12	–	BbuN40_O07	

111 continued on next page

PFam	B31	297	JD1	N40	Comments
111	BB_P24	Bbu297_P24	–	–	
111	BB_Q31	–	BbuJD1_Q26	BbuN40_Q25	
111	BB_R24	Bbu297_R25	–	BbuN40_R02	
111	BB_S24	Bbu297_S24	BbuJD1_S24	–	
111	–	Bbu297_J10*	–	–	
111	–	Bbu297_V24	–	BbuN40_V23	
111	–	Bbu297_W29	BbuJD1_W25*	–	
111	–	Bbu297_X24	BbuJD1_X25	BbuN40_X24	
111	–	Bbu297_Y01*	–	–	
111	–	–	BbuJD1_PV23	–	
111	–	–	BbuJD1_PV66	–	
112	BB_L25	–	BbuJD1_L25	–	
112	BB_M25	Bbu297_M25	BbuJD1_M25	–	
112	BB_N25	Bbu297_N20	BbuJD1_N26	BbuN40_N28	
112	BB_O25	Bbu297_O13	–	BbuN40_O08	
112	BB_P25	Bbu297_P25	–	–	
112	BB_Q32	–	BbuJD1_Q27	BbuN40_Q26	
112	BB_R25	Bbu297_R26	–	BbuN40_R03	
112	BB_S25	Bbu297_S25	BbuJD1_S25	–	
112	–	Bbu297_V25	–	BbuN40_V24	
112	–	Bbu297_W30	BbuJD1_W27	–	
112	–	Bbu297_X25	BbuJD1_X26	BbuN40_X25	
112	–	–	BbuJD1_PV24	–	
112	–	–	BbuJD1_PV67	–	
113					Mlp proteins; antigenic
113	BB_L28	–	BbuJD1_L28	–	B31 MlpH
113	BB_M28	Bbu297_M28	BbuJD1_M28	–	B31 MlpF; 297 Mlp1
113	BB_N28	Bbu297_N24	BbuJD1_N29	BbuN40_N31	B31 MlpI, antigenic; 297 Mlp9

113 continued on next page

PFam	B31	297	JD1	N40	Comments
113	BB_O28	Bbu297_O16	–	BbuN40_O11	B31MlpG; 297 Mlp3
113	BB_P28	Bbu297_P28	–	–	B31MlpA
113	BB_Q35	–	BbuJD1_Q30	BbuN40_Q29	B31MlpJ
113	BB_R28	Bbu297_R29	–	BbuN40_R06	B31MlpD; 297 Mlp7A
113	BB_S30	Bbu297_S29	BbuJD1_S28	–	B31 MlpC, antigenic; 297 Mlp8
113	–	Bbu297_R30	–		297 Mlp7B
113	–	Bbu297_R41	–	–	297 Mlp11
113	–	Bbu297_V28	–	BbuN40_V27	297 Mlp2
113	–	Bbu297_W33	BbuJD1_W30	–	297 Mlp4
113	–	Bbu297_X28	BbuJD1_X29	–	297 Mlp10
113	–	–	BbuJD1_PV28	–	
113	–	–	BbuJD1_PV70	–	
113	–	–	BbuJD1_X46	–	
114	BB_L41	–	BbuJD1_L40	–	
114	BB_N41	Bbu297_N37*	BbuJD1_N47	BbuN40_N48*	
114	BB_O42	–	–	BbuN40_O23	
114	BB_P40	Bbu297_P41	–	–	
114	BB_Q48	–	–	BbuN40_Q44	
114	BB_R43	–	–	–	
114	–	Bbu297_S42	BbuJD1_S39	–	
114	–	Bbu297_V41	–	–	
114	–	Bbu297_X41	–	–	
114	–	–	BbuJD1_M40	–	
114	–	–	BbuJD1_PV81	–	
114	–	–	BbuJD1_W45	–	
115	BB_L42	–	BbuJD1_L41	–	
115	BB_M41	Bbu297_M42	BbuJD1_M41	–	
115	BB_N42	–	BbuJD1_N48	BbuN40_N49	

115 continued on next page

PFam	B31	297	JD1	N40	Comments
115	BB_O43	Bbu297_O30	–	BbuN40_O24	
115	BB_P41	Bbu297_P42	–	–	
115	BB_Q49	–	BbuJD1_Q43	BbuN40_Q45	
115	BB_R44	Bbu297_R42	–	BbuN40_R20	
115	BB_S44	Bbu297_S43	BbuJD1_S40	–	
115	–	Bbu297_N38*	–	–	
115	–	Bbu297_V42	–	–	
115	–	Bbu297_W46	BbuJD1_W46	–	
115	–	Bbu297_X43	BbuJD1_X47	BbuN40_X37	
115	–	–	BbuJD1_PV41	–	
115	–	–	BbuJD1_PV82	–	
115	–	–	–	BbuN40_V39*	
117	BB_G19	–	–	BbuN40_G16*	
117	–	Bbu297_Z15	BbuJD1_Z16	–	
117	–	–	BbuJD1_AA18	–	
118	BB_0098	–	BbuJD1_0098	BbuN40_0098	Predicted DNA repair proteins
118	BB_0797	–	BbuJD1_0797	BbuN40_0797	
119	BB_0136	–	BbuJD1_0136	BbuN40_0136	Predicted peniciilin binding proteins
119	BB_0718	–	BbuJD1_0718	BbuN40_0718	
119	BB_0732	–	BbuJD1_0732	BbuN40_0732	
120	BB_0147	–	BbuJD1_0147	BbuN40_0147	Predicted flagellar proteins
120	BB_0182	–	BbuJD1_0182	BbuN40_0182	
121	BB_0172	–	BbuJD1_0172	BbuN40_0172	
121	BB_0173	–	BbuJD1_0173	BbuN40_0173	
122	BB_0179	–	BbuJD1_0179	BbuN40_0179	Predicted GTPases
122	BB_0508	–	BbuJD1_0508	BbuN40_0508	
122	BB_0643	–	BbuJD1_0643	BbuN40_0643	
122	BB_0660	–	BbuJD1_0660	BbuN40_0660	

PFam	B31	297	JD1	N40	Comments
123	BB_0058	–	BbuJD1_0058	BbuN40_0058	
123	BB_0195	–	BbuJD1_0195	BbuN40_0195	
123	BB_0210	–	BbuJD1_0210	BbuN40_0210	
124	BB_0225	–	BbuJD1_0225	BbuN40_0225	
124	BB_0737	–	BbuJD1_0737	BbuN40_0737	
125	BB_0231	–	BbuJD1_0231	BbuN40_0231	
125	BB_0245	–	BbuJD1_0245	BbuN40_0245	
125	BB_0538	–	BbuJD1_0538	BbuN40_0538	
127	BB_0295	–	BbuJD1_0295	BbuN40_0295	
127	BB_0612	–	BbuJD1_0612	BbuN40_0612	
128	BB_0304	–	BbuJD1_0304	BbuN40_0304	Predicted peptidoglycan D-alanine ligases
128	BB_0585	–	BbuJD1_0585	BbuN40_0585	
128	BB_0817	–	BbuJD1_0817	BbuN40_0817	
129	BB_0316	–	BbuJD1_0316	BbuN40_0316	Predicted integral membrane proteins
129	BB_0317	–	BbuJD1_0317	BbuN40_0317	
130	BB_0678	–	BbuJD1_0678	BbuN40_0678	Predicted ABC transporter permeases
130	BB_0679	–	BbuJD1_0679	BbuN40_0679	
131	BB_0366	–	BbuJD1_0366	BbuN40_0366	Predicted amiopeptidases
131	BB_0627	–	BbuJD1_0627	BbuN40_0627	
132	BB_0415	–	BbuJD1_0415	BbuN40_0415	Predicted chemotaxis protein methylases
132	BB_0568	–	BbuJD1_0568	BbuN40_0568	
133	BB_0471	–	BbuJD1_0471	BbuN40_0471	
133	BB_0505	–	BbuJD1_0505	BbuN40_0505	
135	BB_0637	–	BbuJD1_0637	BbuN40_0637	Predicted Na+H+ antiporter proteins
135	BB_0638	–	BbuJD1_0638	BbuN40_0638	
136	BB_0652	–	BbuJD1_0652	BbuN40_0652	SecD protein secretion
136	BB_0653	–	BbuJD1_0653	BbuN40_0653	SecF protein secretion

PFam	B31	297	JD1	N40	Comments
137	BB_0734	–	BbuJD1_0734	BbuN40_0734	
137	BB_T06	–	–	–	
137	BB_U08*	–	–	–	297 homologs on chromosome (Huang
137	BB_U11	–	–	–	et al. 2004 J. Bact. 186:4134)
137	–	–	BbuJD1_J24	–	in region of local synteny with B31 lp21
138	BB_0852	–	BbuJD1_0852	–	
138	BB_Q69*	–	–	–	
138	–	Bbu297_F38	BbuJD1_F33	–	
139	BB_A08	Bbu297_A08	BbuJD1_A08	BbuN40_A08	
139	BB_L19	–	BbuJD1_L19	–	
139	BB_M19	Bbu297_M19	BbuJD1_M19	–	
139	BB_N19	Bbu297_N09	BbuJD1_N20	BbuN40_N21	
139	BB_O19*	–	–	BbuN40_O02	
139	BB_P19	Bbu297_P19	–	–	
139	BB_Q26	–	BbuJD1_Q21	BbuN40_Q19	
139	BB_R19	Bbu297_R20	–	–	
139	BB_S19	Bbu297_S19	BbuJD1_S19	–	
139	–	Bbu297_V19	–	BbuN40_V18	
139	–	Bbu297_W24	BbuJD1_W20	–	
139	–	Bbu297_X19	BbuJD1_X20	BbuN40_X19	
139	–	–	BbuJD1_PV18*	–	
139	–	–	BbuJD1_PV61*	–	
140	BB_A09	Bbu297_A09	BbuJD1_A09	BbuN40_A09	
140	BB_L20	–	BbuJD1_L20	–	
140	BB_M20	Bbu297_M20	BbuJD1_M20	–	
140	BB_N20	Bbu297_N10	BbuJD1_N21	BbuN40_N22	
140	BB_O20	–	–	BbuN40_O03	
140	BB_P20	Bbu297_P20	–	–	

140 continued on next page

PFam	B31	297	JD1	N40	Comments
140	BB_Q27	–	BbuJD1_Q22	BbuN40_Q20	
140	BB_R20	Bbu297_R21	–	–	
140	BB_S20	Bbu297_S20	BbuJD1_S20	–	
140	–	Bbu297_V20	–	BbuN40_V19	
140	–	Bbu297_W25	BbuJD1_W21	–	
140	–	Bbu297_X20	BbuJD1_X21	BbuN40_X20	
140	–	–	BbuJD1_PV19*	–	
140	–	–	BbuJD1_PV62	–	
141	BB_A10	Bbu297_A10	BbuJD1_A10	BbuN40_A10	
141	BB_L21	–	BbuJD1_L21	–	
141	BB_M21	Bbu297_M21	BbuJD1_M21	–	
141	BB_N21*	Bbu297_N11	BbuJD1_N22	BbuN40_N23	
141	BB_O21	–	–	BbuN40_O04*	
141	BB_P21	Bbu297_P21	–	–	
141	BB_Q28	–	BbuJD1_Q23	BbuN40_Q21	
141	BB_R21	Bbu297_R22	–	–	
141	BB_S21	Bbu297_S21	BbuJD1_S21	–	
141	–	Bbu297_V21	–	BbuN40_V20	
141	–	Bbu297_W26	BbuJD1_W22	–	
141	–	Bbu297_X21	BbuJD1_X22	BbuN40_X21	
141	–	–	BbuJD1_PV20*	–	
141	–	–	BbuJD1_PV63*	–	
142	BB_A11	Bbu297_A11	BbuJD1_A11	BbuN40_A11	
142	BB_L22	–	BbuJD1_L22	–	
142	BB_M22	Bbu297_M22	BbuJD1_M22	–	
142	BB_N22*	Bbu297_N12	BbuJD1_N23	BbuN40_N24	
142	BB_O22	Bbu297_O10	–	BbuN40_O05*	
142	BB_P22	Bbu297_P22	–	–	

142 continued on next page

PFam	B31	297	JD1	N40	Comments
142	BB_Q29	–	BbuJD1_Q24	BbuN40_Q22	
142	BB_R22	Bbu297_R23	–	–	
142	BB_S22	Bbu297_S22	BbuJD1_S22	–	
142	–	Bbu297_V22	–	BbuN40_V21	
142	–	Bbu297_W27	BbuJD1_W23	–	
142	–	Bbu297_X22	BbuJD1_X23	BbuN40_X22	
142	–	–	BbuJD1_PV21	–	
142	–	–	BbuJD1_PV64	–	
143	BB_A14	Bbu297_A14	BbuJD1_A14	BbuN40_A14	
143	BB_G25	–	–	BbuN40_G23	
143	BB_L26	–	BbuJD1_L26	–	
143	BB_M26	Bbu297_M26	BbuJD1_M26	–	
143	BB_N26	Bbu297_N22	BbuJD1_N27	BbuN40_N29	
143	BB_O26	Bbu297_O14	–	BbuN40_O09	
143	BB_P26	Bbu297_P26	–	–	
143	BB_Q33	–	BbuJD1_Q28	BbuN40_Q27	
143	BB_R26	Bbu297_R27	–	BbuN40_R04	
143	BB_S26	Bbu297_S26	BbuJD1_S26	–	
143	–	Bbu297_V26	–	BbuN40_V25	
143	–	Bbu297_W31	BbuJD1_W28	–	
143	–	Bbu297_X26	BbuJD1_X27	BbuN40_X26	
143	–	Bbu297_Z23	BbuJD1_Z24	–	
143	–	–	BbuJD1_AA26	–	
143	–	–	BbuJD1_PV25	–	
143	–	–	BbuJD1_PV68*	–	
144					BppB; previously called "Orf-10"
144	BB_A23	Bbu297_A23	BbuJD1_A23	BbuN40_A23	
144	BB_G27	–	–	BbuN40_G25	

144 continued on next page

PFam	B31	297	JD1	N40	Comments
144	BB_L37	–	BbuJD1_L36	–	
144	BB_M36	Bbu297_M36	BbuJD1_M36	–	B31 M36 antigenic
144	BB_N36	Bbu297_N33	BbuJD1_N40	BbuN40_N41	
144	BB_O37	Bbu297_O25	–	BbuN40_O20	
144	BB_P36	Bbu297_P36	–	–	
144	BB_Q44	–	BbuJD1_Q38	BbuN40_Q38	
144	BB_R37	Bbu297_R38	–	BbuN40_R14	
144	BB_S39	Bbu297_S39	BbuJD1_S36	–	
144	–	Bbu297_V36	–	BbuN40_V35	
144	–	Bbu297_W41	BbuJD1_W40	–	
144	–	Bbu297_X36	BbuJD1_X41	BbuN40_X34	
144	–	Bbu297_Z25	BbuJD1_Z26	–	
144	–	–	BbuJD1_AA28	–	
144	–	–	BbuJD1_PV36*	–	
144	–	–	BbuJD1_PV78	–	
145					Putative bacteriophage DNA packaging enzyme, terminase
145	BB_A31	Bbu297_A31	BbuJD1_A31	BbuN40_A31	
145	BB_L43	–	BbuJD1_L42	–	
145	BB_M42	Bbu297_M43	BbuJD1_M42	–	
145	BB_N43	–	BbuJD1_N49	BbuN40_N51*	
145	BB_O44	Bbu297_O31*	–	BbuN40_O25	
145	BB_P42	Bbu297_P43	–	–	
145	BB_Q50	–	BbuJD1_Q44	BbuN40_Q46	
145	BB_R45	Bbu297_R43	–	BbuN40_R21	
145	BB_S45	Bbu297_S44	BbuJD1_S41	–	
145	–	Bbu297_V43	–	BbuN40_V40*	
145	–	Bbu297_W47	BbuJD1_W47	–	

145 continued on next page

PFam	B31	297	JD1	N40	Comments
145	–	Bbu297_X44	BbuJD1_X48	BbuN40_X38	
145	–	–	BbuJD1_PV42	–	
145	–	–	BbuJD1_PV83	–	
146					Putative bacteriophage portal protein
146	BB_A38	Bbu297_A38	BbuJD1_A38	BbuN40_A38	
146	BB_L01	–	BbuJD1_L01	–	
146	BB_M01	Bbu297_M01	BbuJD1_M01	–	
146	BB_N01	–	BbuJD1_N01	BbuN40_N01*	
146	BB_O01	Bbu297_O01*	–	–	
146	BB_P01	Bbu297_P01	–	–	
146	BB_Q51*	–	BbuJD1_Q01	BbuN40_Q01	
146	BB_R01	Bbu297_R01	–	BbuN40_R01*	
146	BB_S01	Bbu297_S01	BbuJD1_S01	–	
146	–	Bbu297_V01	–	BbuN40_V01	
146	–	Bbu297_W01	BbuJD1_W01	–	
146	–	Bbu297_X01	BbuJD1_X01	BbuN40_X01	
146	–	–	BbuJD1_PV01	–	
146	–	–	BbuJD1_PV43*	–	
147	BB_A39	Bbu297_A39	BbuJD1_A39	BbuN40_A39	
147	BB_L02	–	BbuJD1_L02	–	B31 L02 antigenic
147	BB_M02	Bbu297_M02	BbuJD1_M02	–	
147	BB_N02	–	BbuJD1_N02	BbuN40_N02	
147	BB_O02	Bbu297_O02	–	–	
147	BB_P02	Bbu297_P02	–	–	
147	BB_Q52	–	BbuJD1_Q02	BbuN40_Q02*	
147	BB_R02*	Bbu297_R02*	–	–	
147	BB_S02	Bbu297_S02	BbuJD1_S02	–	
147	–	Bbu297_V02	–	BbuN40_V02	

147 continued on next page

PFam	B31	297	JD1	N40	Comments
147	–	Bbu297_W02	BbuJD1_W02	–	
147	–	Bbu297_X02	BbuJD1_X02	BbuN40_X02	
147	–	–	BbuJD1_PV02	–	
147	–	–	BbuJD1_PV44	–	
148	BB_A40	Bbu297_A40	BbuJD1_A40	BbuN40_A40	
148	BB_L03	–	BbuJD1_L03	–	
148	BB_L04	–	BbuJD1_L04	–	
148	BB_L05	–	BbuJD1_L05	–	
148	BB_M03	Bbu297_M03	BbuJD1_M03	–	
148	BB_M04	Bbu297_M04	BbuJD1_M04	–	
148	BB_M05	Bbu297_M05	BbuJD1_M05	–	
148	BB_N03	–	BbuJD1_N03	BbuN40_N03	
148	BB_N04	–	BbuJD1_N05	BbuN40_N05	
148	BB_N05*	–	BbuJD1_N06	BbuN40_N07*	
148	BB_O03	Bbu297_O03	–	–	
148	BB_O04	Bbu297_O04	–	–	
148	BB_O05	Bbu297_O05	–	–	
148	BB_P03	Bbu297_P03	–	–	
148	BB_P04	Bbu297_P04	–	–	
148	BB_P05	Bbu297_P05	–	–	
148	BB_Q11	–	BbuJD1_Q03	BbuN40_Q03	
148	BB_Q12	–	BbuJD1_Q04	BbuN40_Q04	
148	BB_Q53	–	BbuJD1_Q05	BbuN40_Q05	
148	BB_Q54*	–	–	–	
148	BB_R03	Bbu297_R04	–	–	
148	BB_R04	Bbu297_R05	–	–	
148	BB_R05	Bbu297_R06	–	–	
148	BB_S03	Bbu297_S03	BbuJD1_S03	–	

148 continued on next page

PFam	B31	297	JD1	N40	Comments
148	BB_S04	Bbu297_S04	BbuJD1_S04	–	
148	BB_S05	Bbu297_S05	BbuJD1_S05	–	
148	–	Bbu297_V03	–	BbuN40_V03	
148	–	Bbu297_V04	–	BbuN40_V04	
148	–	Bbu297_V05	–	BbuN40_V05	
148	–	Bbu297_W03	BbuJD1_W03	–	
148	–	Bbu297_W04	BbuJD1_W05	–	
148	–	Bbu297_W05	BbuJD1_W06	–	
148	–	Bbu297_X03	BbuJD1_X03	BbuN40_X03	
148	–	Bbu297_X04	BbuJD1_X05	BbuN40_X04	
148	–	Bbu297_X05	BbuJD1_X06	BbuN40_X05	
148	–	–	BbuJD1_PV03*	–	
148	–	–	BbuJD1_PV04	–	
148	–	–	BbuJD1_PV45	–	
148	–	–	BbuJD1_PV46	–	
148	–	–	BbuJD1_PV47	–	
149	BB_A41	Bbu297_A41	BbuJD1_A41	BbuN40_A41	
149	BB_L06	–	BbuJD1_L06	–	
149	BB_M06	Bbu297_M06	BbuJD1_M06	–	
149	BB_N06*	–	BbuJD1_N07	BbuN40_N08	
149	BB_O06	Bbu297_O06	–	–	
149	BB_P06	Bbu297_P06	–	–	
149	BB_Q13	–	BbuJD1_Q06	BbuN40_Q06	B31 Q13 is antigenic
149	BB_R06	Bbu297_R07	–	–	
149	BB_S06	Bbu297_S06	BbuJD1_S06	–	
149	–	Bbu297_V06	–	BbuN40_V06	
149	–	Bbu297_W06	BbuJD1_W07	–	
149	–	Bbu297_X06	BbuJD1_X07	BbuN40_X06	

149 continued on next page

PFam	B31	297	JD1	N40	Comments
149	–	–	BbuJD1_PV05*	–	
149	–	–	BbuJD1_PV48	–	
150	BB_A42	Bbu297_A42	BbuJD1_A42	BbuN40_A42	
150	BB_L07	–	BbuJD1_L07	–	
150	BB_M07	Bbu297_M07	BbuJD1_M07	–	
150	BB_N07	–	BbuJD1_N08	BbuN40_N09	
150	BB_O07	Bbu297_O07	–	–	
150	BB_P07	Bbu297_P07	–	–	
150	BB_Q14	–	BbuJD1_Q07	BbuN40_Q07	
150	BB_R07	Bbu297_R08	–	–	
150	BB_S07	Bbu297_S07	BbuJD1_S07	–	
150	–	Bbu297_V07	–	BbuN40_V07	
150	–	Bbu297_W08	BbuJD1_W08	–	
150	–	Bbu297_X07	BbuJD1_X08	BbuN40_X07	
150	–	–	BbuJD1_PV06	–	
150	–	–	BbuJD1_PV49	–	
151	BB_A45	Bbu297_A45	BbuJD1_A45	BbuN40_A45	
151	BB_L10	–	BbuJD1_L10	–	
151	BB_M10	Bbu297_M10	BbuJD1_M10	–	
151	BB_N10	–	BbuJD1_N11	BbuN40_N12	
151	BB_O10	–	–	–	
151	BB_P10	Bbu297_P10	–	–	
151	BB_Q17	–	BbuJD1_Q10	BbuN40_Q10	
151	BB_R10	Bbu297_R11	–	–	
151	BB_S10	Bbu297_S10	BbuJD1_S10	–	
151	–	Bbu297_V10	–	BbuN40_V10*	
151	–	Bbu297_W14	–	–	
151	–	Bbu297_X10	BbuJD1_X11	BbuN40_X10	

151 continued on next page

PFam	B31	297	JD1	N40	Comments
151	–	–	BbuJD1_PV09	–	
151	–	–	BbuJD1_PV52	–	
151	–	–	BbuJD1_W11	–	
152	BB_A46	Bbu297_A46	BbuJD1_A46	BbuN40_A46	
152	BB_L11	–	BbuJD1_L11	–	
152	BB_M11	Bbu297_M11	BbuJD1_M11	–	
152	BB_N11	–	BbuJD1_N12	BbuN40_N13*	B31 N11 iis antigenic
152	BB_O11	–	–	–	
152	BB_P11	Bbu297_P11	–	–	
152	BB_Q18	–	BbuJD1_Q11	BbuN40_Q11	
152	BB_R11	Bbu297_R12	–	–	
152	BB_S11	Bbu297_S11	BbuJD1_S11	–	
152	–	Bbu297_V11	–	BbuN40_V17*	
152	–	Bbu297_W16	BbuJD1_W12	–	
152	–	Bbu297_X11	BbuJD1_X12	BbuN40_X11	
152	–	–	BbuJD1_PV10	–	
152	–	–	BbuJD1_PV53*	–	
153	BB_A47	Bbu297_A47	BbuJD1_A47	BbuN40_A47	
153	BB_L12	–	BbuJD1_L12	–	
153	BB_M12	Bbu297_M12	BbuJD1_M12	–	
153	BB_N12	–	BbuJD1_N13	BbuN40_N14	
153	BB_O12	–	–	–	
153	BB_P12	Bbu297_P12	–	–	
153	BB_Q19	–	BbuJD1_Q12	BbuN40_Q12	
153	BB_R12	Bbu297_R13	–	–	BB_R12 is antigenic
153	BB_S12	Bbu297_S12	BbuJD1_S12	–	
153	–	Bbu297_V12	–	BbuN40_V16	
153	–	Bbu297_W17	BbuJD1_W13	–	

153 continued on next page

PFam	B31	297	JD1	N40	Comments
153	–	Bbu297_X12	BbuJD1_X13	BbuN40_X12	
153	–	–	BbuJD1_PV11	–	
153	–	–	BbuJD1_PV54	–	
154	BB_A48	Bbu297_A48	BbuJD1_A48	BbuN40_A48	B31 A48 is antigenic
154	BB_L13	–	BbuJD1_L13	–	
154	BB_M13	Bbu297_M13	BbuJD1_M13	–	
154	BB_N13*	Bbu297_N02*	BbuJD1_N14	BbuN40_N15*	
154	BB_O13	–	–	–	
154	BB_P13	Bbu297_P13	–	–	
154	BB_Q20	–	BbuJD1_Q13	BbuN40_Q13	
154	BB_R13	Bbu297_R14	–	–	
154	BB_S13	Bbu297_S13	BbuJD1_S13	–	
154	–	Bbu297_V13	–	BbuN40_V15	
154	–	Bbu297_W18	BbuJD1_W14	–	
154	–	Bbu297_X13	BbuJD1_X14	BbuN40_X13	
154	–	–	BbuJD1_PV12	–	
154	–	–	BbuJD1_PV55	–	
155	BB_A49	Bbu297_A49	BbuJD1_A49	BbuN40_A49	
155	BB_L14	–	BbuJD1_L14	–	
155	BB_M14	Bbu297_M14	BbuJD1_M14	–	
155	BB_N14	Bbu297_N04	BbuJD1_N15	BbuN40_N16	
155	BB_O14	–	–	–	
155	BB_P14	Bbu297_P14	–	–	
155	BB_Q21	–	BbuJD1_Q14	BbuN40_Q14	
155	BB_R14	Bbu297_R15	–	–	
155	BB_S14	Bbu297_S14	BbuJD1_S14	–	
155	–	Bbu297_V14	–	BbuN40_V14	
155	–	Bbu297_W19	BbuJD1_W15	–	

155 continued on next page

PFam	B31	297	JD1	N40	Comments
155	–	Bbu297_X14	BbuJD1_X15	BbuN40_X14	
155	–	–	BbuJD1_PV13	–	
155	–	–	BbuJD1_PV56	–	
156	BB_L15	–	BbuJD1_L15	–	
156	BB_M15	Bbu297_M15	BbuJD1_M15	–	
156	BB_N15	Bbu297_N05	BbuJD1_N16	BbuN40_N17	
156	BB_O15	–	–	–	
156	BB_P15	Bbu297_P15	–	–	
156	BB_Q22	–	BbuJD1_Q15	BbuN40_Q15	
156	BB_R15	Bbu297_R16	–	–	
156	BB_S15	Bbu297_S15	BbuJD1_S15	–	
156	–	Bbu297_V15	–	BbuN40_V13	
156	–	Bbu297_W20	BbuJD1_W16	–	
156	–	Bbu297_X15	BbuJD1_X16	BbuN40_X15	
156	–	–	BbuJD1_PV14*	–	
156	–	–	BbuJD1_PV57	–	
157	BB_A51	Bbu297_A51	BbuJD1_A51	BbuN40_A51	
157	BB_L16	–	BbuJD1_L16	–	
157	BB_M16	Bbu297_M16	BbuJD1_M16	–	
157	BB_N16*	Bbu297_N06	BbuJD1_N17	BbuN40_N18	
157	BB_O16	–	–	–	
157	BB_P16	Bbu297_P16	–	–	
157	BB_Q23	–	BbuJD1_Q16	BbuN40_Q16	
157	BB_R16	Bbu297_R17	–	–	
157	BB_S16	Bbu297_S16	BbuJD1_S16	–	
157	–	Bbu297_V16	–	BbuN40_V12	
157	–	Bbu297_W21	BbuJD1_W17	–	
157	–	Bbu297_X16	BbuJD1_X17	BbuN40_X16	

157 continued on next page

PFam	B31	297	JD1	N40	Comments
157	–	–	BbuJD1_PV15	–	
157	–	–	BbuJD1_PV58	–	
158	BB_A53	Bbu297_A53	BbuJD1_A53	BbuN40_A53	weakly related to B31 BB_S27
158	BB_A54	Bbu297_A54	BbuJD1_A54	BbuN40_A54	
159	BB_A55	Bbu297_A55	BbuJD1_A55	BbuN40_A55	
159	BB_L17	–	BbuJD1_L17	–	
159	BB_M17	Bbu297_M17	BbuJD1_M17	–	
159	BB_N17	Bbu297_N07	BbuJD1_N18	BbuN40_N19	
159	BB_O17	–	–	–	
159	BB_P17	Bbu297_P17	–	–	
159	BB_Q24	–	BbuJD1_Q17*	BbuN40_Q17	
159	BB_R17	Bbu297_R18	–	–	
159	BB_S17	Bbu297_S17	BbuJD1_S17	–	
159	–	Bbu297_V17	–	BbuN40_V11	
159	–	Bbu297_W22	BbuJD1_W18	–	
159	–	Bbu297_X17	BbuJD1_X18	BbuN40_X17	
159	–	–	BbuJD1_PV16	–	
159	–	–	BbuJD1_PV59	–	
160	BB_A56	Bbu297_A56	BbuJD1_A56	BbuN40_A56	
160	BB_L18	–	BbuJD1_L18	–	
160	BB_M18	Bbu297_M18	BbuJD1_M18	–	
160	BB_N18*	Bbu297_N08	BbuJD1_N19	BbuN40_N20	
160	BB_O18	–	–	BbuN40_O01	
160	BB_P18	Bbu297_P18	–	–	
160	BB_Q25	–	BbuJD1_Q19*	BbuN40_Q18	
160	BB_R18	Bbu297_R19	–	–	
160	BB_S18	Bbu297_S18	BbuJD1_S18	–	
160	–	Bbu297_V18	–	BbuN40_V41*	

160 continued on next page

PFam	B31	297	JD1	N40	Comments
160	–	Bbu297_W23	BbuJD1_W19	–	
160	–	Bbu297_X18	BbuJD1_X19	BbuN40_X18	
160	–	–	BbuJD1_PV17	–	
160	–	–	BbuJD1_PV60*	–	
161	BB_C05	–	–	BbuN40_C04	
161	BB_L29	–	BbuJD1_L29	–	
161	BB_M29	Bbu297_M29	BbuJD1_M29	–	
161	BB_N29*	Bbu297_N25	BbuJD1_N30	BbuN40_N33	
161	BB_O29	Bbu297_O17	–	BbuN40_O12	
161	BB_P29	Bbu297_P29	–	–	
161	BB_Q37	–	BbuJD1_Q31	BbuN40_Q30	
161	BB_R29	Bbu297_R31	–	BbuN40_R07	
161	BB_R41	–	–	–	
161	BB_S31	Bbu297_S30	BbuJD1_S29	–	
161	–	Bbu297_V29	–	BbuN40_V28*	
161	–	Bbu297_W34	BbuJD1_W32	–	
161	–	Bbu297_X29	BbuJD1_X30	BbuN40_X27	
161	–	–	BbuJD1_M38	–	
161	–	–	BbuJD1_PV29	–	
161	–	–	BbuJD1_PV71	–	

PFam	B31	297	JD1	N40	Comments
163					Surface lipoproteins. PFam163 results from merging PFams162, 163 and 164 of Casjens et al. Molec. Micro. 35 (2000) 490. These can be separated into several rather different subgroups (see also Akins *et al.,* Infect. Immun. 67 (1999) 1526-1532 and Stevenson & Miller, J. Mol Evol. 57 (2003) 309).
163	BB_F01	–	–	–	B31 F01/Arp; antibodies resolve arthritis
163	BB_L39	–	BbuJD1_L38	–	B31 ErpA8; N40 Erp50
163	BB_L40	–	BbuJD1_L39	–	B31 ErpB8; N40 Erp51
163	BB_M38	Bbu297_M38	BbuJD1_M39	–	B31 ErpK; 297 Bbk2.10
163	BB_N38	Bbu297_N35	BbuJD1_N44	BbuN40_N44	B31 ErpP/complement regulator-acquiring surface protein 3 (CRASP-3), antigenic, binds plasminogen; N40 p21; 297 P21
163	BB_N39	Bbu297_N36	BbuJD1_N45	BbuN40_N45	B31 ErpQ, antigenic; N40 Erp22; 297 ElpB2
163	BB_O39	Bbu297_O28	–	BbuN40_O26	B31 ErpL, antigenic; N40 OspE; 297 ElpA2
163	BB_O40	–	–	BbuN40_O27	B31 ErpM, antigenic; N40 OspF
163	BB_P38	Bbu297_P38	–	–	B31 ErpA1/Complement regulator-acquiring surface protein-5 (CRASP-5), binds plasminogen; 297 OspE-1
163	BB_P39	Bbu297_P39	–	–	B31 ErpB1, antigenic; 297 ElpB-1

163 continued on next page

PFam	B31	297	JD1	N40	Comments
163	BB_Q47	–	BbuJD1_Q40	BbuN40_Q42	B31 ErpX, antigenic, surface protein, binds laminin; N40 Erp26
163	BB_R42	Bbu297_R40	–	BbuN40_R18	B31 EprY, antigenic; N40 Erp23; 297 ElpA1
163	BB_S41	Bbu297_S41	BbuJD1_S38	–	B31 ErpG, antigenic; 297 OspF
163	–	Bbu297_V38	–	BbuN40_V37	
163	–	Bbu297_V39	–	–	
163	–	Bbu297_W43	BbuJD1_W44	–	297 Bbk2.11
163	–	Bbu297_X38	BbuJD1_X44	BbuN40_X36	N40 Erp27; 297 OspE-12
163	–	Bbu297_X39	–	–	297 ElpB1-12
163	–	–	BbuJD1_I37	–	in region of syntenic with, and very similar to B31 BB_F01
163	–	–	BbuJD1_PV38	–	
163	–	–	BbuJD1_PV80	–	
163	–	–	–	BbuN40_R19	N40 Erp24
163	–	–	–	BbuN40_Y16	in region of syntenic with, and very similar to B31 BB_F01/Arp
165					BppA; previously called "Orf-6/7; weak similarity to exonucleases
165	BB_C12	–	–	BbuN40_C13	
165	BB_L36	–	BbuJD1_L35	–	
165	BB_M35	Bbu297_M35	BbuJD1_M35	–	
165	BB_N35	Bbu297_N31*	BbuJD1_N39	BbuN40_N40	
165	BB_O36	Bbu297_O24	–	BbuN40_O19*	
165	BB_P35	Bbu297_P35	–	–	
165	BB_Q43	–	BbuJD1_Q37	BbuN40_Q37	
165	BB_R36	Bbu297_R37	–	BbuN40_R13*	
165	BB_S38	Bbu297_S38	BbuJD1_S35	–	

165 continued on next page

PFam	B31	297	JD1	N40	Comments
165	–	Bbu297_V35	–	BbuN40_V34	
165	–	Bbu297_W40	BbuJD1_W39	–	
165	–	Bbu297_X35	BbuJD1_X40	BbuN40_X33	
165	–	–	BbuJD1_PV35*	–	
165	–	–	BbuJD1_PV77	–	
167	BB_J20*	–	–	BbuN40_J11*	
167	BB_Q67	–	–	–	B31 BB_Q67 fused to PFam102 part
170					VlsE and cassettes
170	BB_F0041	–	BbuJD1_F36	–	VlsE outer surface protein
170	BB_F32*	Bbu297_F26*	BbuJD1_F24*	–	vlsE cassettes
170	BB_J51*	–	–	BbuN40_J34*	
170	–	–	BbuJD1_Z01	–	
170	–	–	–	BbuN40_K37*	
171	BB_A74	–	BbuJD1_A74	BbuN40_A74	B31 A74 is surface protein
171	BB_H20*	Bbu297_H15*	BbuJD1_H23*	–	
171	BB_J11	–	–	BbuN40_J35*	
175	BB_F19.1*	–	–	–	
175	BB_K37*	–	–	–	BBK37 has PFam75 sequences at its N-terminal region and PFam175 at its C-terminal. Hence it is listed in both PFams
176	BB_0022	–	BbuJD1_0022	BbuN40_0022	Predicted RuvB type helicase
176	BB_0461	–	BbuJD1_0461	BbuN40_0461	similarity to DNA polymerase III gamma subunit
176	BB_0765	–	BbuJD1_0765	BbuN40_0765	
177	BB_0052	–	BbuJD1_052	BbuN40_0052	Predicted RNA methyltransferases
177	BB_0516*	–	BbuJD1_0516	BbuN40_0516	

PFam	B31	297	JD1	N40	Comments
178	BB_0101	–	BbuJD1_0101	BbuN40_0101	asparaginyl-tRNA synthetase
178	BB_0446	–	BbuJD1_0446	BbuN40_0446	aspartyl-tRNA synthetase
179	BB_0235	–	BbuJD1_0235	BbuN40_0235	Predicted GTP-binding protein
179	BB_0781	–	BbuJD1_0781	BbuN40_0781	
180	BB_0733	–	BbuJD1_0733	BbuN40_0733	Predicted cyclic-di-GMP binding protein
180	–	Bbu297_F15*	BbuJD1_F10*	–	
180	–	–	BbuJD1_0904	–	local synteny with N40 lp28-5
180	–	–	–	BbuN40_Y05	
181	BB_G0036	–	–	BbuN40_G19	all genes in thisPfam are in locally syntenic positions
181	–	Bbu297_Z19	BbuJD1_Z20	–	
181	–	–	BbuJD1_AA22	–	
182	BB_G12	–	–	BbuN40_G11	all genes in thisPfam are in locally syntenic positions
182	–	Bbu297_Z08	BbuJD1_Z09	–	
182	–	–	BbuJD1_AA11	–	
183	BB_G13	–	–	BbuN40_G12	all genes in thisPfam are in locally syntenic positions
183	–	Bbu297_Z09	BbuJD1_Z10	–	
183	–	–	BbuJD1_AA12	–	
184	BB_G14	–	–	BbuN40_G35	all genes in thisPfam are in locally syntenic positions
184	–	Bbu297_Z10	BbuJD1_Z11	–	
184	–	–	BbuJD1_AA13	–	
185	BB_G15	–	–	BbuN40_G36	all genes in thisPfam are in locally syntenic positions
185	–	Bbu297_Z12	BbuJD1_Z13	–	
185	–	–	BbuJD1_AA15	–	

PFam	B31	297	JD1	N40	Comments
186	BB_G16	–	–	BbuN40_G13	all genes in thisPfam are in locally syntenic positions
186	–	Bbu297_Z11	BbuJD1_Z12	–	
186	–	–	BbuJD1_AA14	–	
187	BB_G17	–	–	BbuN40_G14	all genes in thisPfam are in locally syntenic positions
187	–	Bbu297_Z13	BbuJD1_Z14	–	
187	–	–	BbuJD1_AA16	–	
188	BB_G18	Bbu297_Z14	BbuJD1_Z15	BbuN40_G15	all genes in thisPfam are in locally syntenic positions
188	–	Bbu297_Z14	BbuJD1_Z15	–	
188	–	–	BbuJD1_AA17	–	
189	BB_G21	–	–	BbuN40_G18	all genes in thisPfam are in locally syntenic positions
189	–	Bbu297_Z18	BbuJD1_Z19	–	
189	–	–	BbuJD1_AA21	–	
190	BB_G26	–	–	BbuN40_G24	all genes in thisPfam are in locally syntenic positions
190	–	Bbu297_Z24	BbuJD1_Z25	–	
190	–	–	BbuJD1_AA27	–	
191	BB_G28	–	–	BbuN40_G26	all genes in thisPfam are in locally syntenic positions
191	–	Bbu297_Z26	BbuJD1_Z27	–	
191	–	–	BbuJD1_AA29	–	
192	BB_G30	–	–	BbuN40_G28	all genes in thisPfam are in locally syntenic positions
192	–	–	BbuJD1_Z30	–	
192	–	–	BbuJD1_AA32	–	

PFam	B31	297	JD1	N40	Comments
193	–	Bbu297_Y03	BbuJD1_Y12	BbuN40_Y18	weak similarity to B31 BB_J46
193	–		BbuJD1_0906	–	
194	–	–	BbuJD1_Y10	BbuN40_Y02	
194	–	–	BbuJD1_0909	–	
