# Supplementary material for: Genome Stability of Lyme Disease Spirochetes: Comparative Genomics of Borrelia burgdorferi Plasmids
Source: PLoS One. 2012 Mar 14;7(3):e33280. doi: 10.1371/journal.pone.0033280 (PMC3303823; doi:10.1371/journal.pone.0033280)
Supplement: Table S3 — Major tandem repeat tracts. (PDF) [file pone.0033280.s010.pdf]

Table S3

Major tandem repeat tracts

replicon	location	unit repeat length (bp)	B31	N40	JD1	297
chrm	*bb0210 (lmp1)*	162	7[a]	2	6	nd[c]
chrm	*bb0546*	60	5	3	5	nd
chrm	*bb0801*	33	12	10	12	nd
lp17	13154 in B31	21	8	20	9	15
lp21	3618 in B31	63	176	–[b]	126[d]	–
lp28-4	*B31_bbi16/vraA*	27	22	31	24	35
lp28-5	12056 in JD1	133	–	16	26	6
lp38	5938 in B31	17	7	6	–	–
lp38	10287 in B31	7	12	119	–	–
lp54	11362 in B31	8	2	7	3	10
lp54	20128 in B31	11	7	5	3	3
lp54	27258 in B31	10	4	3	3	4

Table S3 footnotes:

a. values rounded to nearest whole integer
b. "–", orthologous sequence not present in this strain
c. nd, not sequenced
d. in variable part of chromosome
