# Supplementary material for: Genome Stability of Lyme Disease Spirochetes: Comparative Genomics of Borrelia burgdorferi Plasmids
Source: PLoS One. 2012 Mar 14;7(3):e33280. doi: 10.1371/journal.pone.0033280 (PMC3303823; doi:10.1371/journal.pone.0033280)
Supplement: Table S4 — Lyme agent Borrelia plasmid letter appellations for locus tags. (PDF) [file pone.0033280.s011.pdf]

Table S4
Lyme agent *Borrelia* plasmid letter appellations for locus tags

Plasmid Name[a]	Letter designation[a]	Example locus tag[a]
Chromosome	no letter	BbuN40_0001
lp54	A	BbuN40_A01
cp26	B	BbuN40_B01
cp9-1	C	BbuN40_C01
lp17	D	BbuN40_D01
lp25	E	BbuN40_E01
lp28-1[b]	F[b]	BbuN40_F01
lp28-2	G	BbuN40_G01
lp28-3	H	BbuN40_H01
lp28-4	I	BbuN40_I01
lp36	K	BbuN40_K01
lp38	J	BbuN40_J01
cp32-1	P	BbuN40_P01
cp32-3	S	BbuN40_S01
cp32-4	R	BbuN40_R01
cp32-6	M	BbuN40_M01
cp32-7 (& -2[c])	O	BbuN40_O01
cp32-8	L	BbuN40_L01
cp32-9	N	BbuN40_N01
cp32-10[d,e]	Q[d,e]	BbuN40_J01
lp5	T	BbuN40_T01
lp21	U	BbuN40_U01
Plasmids not present in the strain B31 genome sequence		
cp32-5	V	BbuN40_V01
cp32-11	W	BbuN40_W01
cp32-12	X	BbuN40_X01
lp28-5	Y	BbuN40_Y01
lp28-6	Z	BbuN40_Z01
lp28-7	AA	BbuJD1_AA01
lp28-8[e]	AC[e]	Bbu94a_AC01
lp28-9[e]	AG[e]	BbuBol26_AG01
lp56[d]	AD[d]	Bbu94a_AD01
cp32-13[e]	AF[e]	Bbu72a_AB01
lp32-3, -6, 10 & -12[f]		see footnote f
Plasmids that carry apparently intact partition gene clusters of two different types		
cp32-1+5[a]	PV	BbuJD1_PV01
cp32-3+8[a]	SL	Bbu64a_SL01
cp32-3+10[a]	SQ	BbuZS7_SQ01
cp32-7+9[a]	ON	Bbu118a_ON01
cp32-quad	NXAF	BbiDN127_NXAF01
Apparent plasmid fragments that carry no PFam32 genes		
Unknown	ZZ	BbuJD1_ZZ01

Table S4 Footnotes:

a. Plasmid names (and thus locus_tag letter designation) were determined by the apparent "compatibility type" of the PFam32 plasmid partitioning gene on the plasmid as determined by sequence comparison (see text).
   If there are two PFam32 genes on a plasmid (rare, but it occurs):
   i. Use the one that is in the more "intact" replication/partition type gene cluster (see "b" below).
   ii. In cases where both are in an apparently intact cluster, *e.g.*, the fused cp32-1/cp32-5 plasmid cp32-1+5 in strain JD1, both letters are used to give the locus tag "BbuJD1_PV01".
   iii. We have identified three other fused cp32s in *B. burgdorferi* strains 64a, ZS7 and 118a [1] and nomenclature for these is shown in the table.

b. There are two PFam32 genes on B31 lp28-1
   i. B31 lp28-1 has two PFam32 genes, *f13* and *f24*. The *f13* is in an incomplete (apparently partly deleted) cluster and *f24* is in the complete cluster.
   ii. To date, *f13* on B31 lp28-1 has not been found on other *Borrelia* plasmids.
   iii. Therefore we gave "F" locus_tags to plasmids with an *f24* type PFam32 gene and recommend giving a new letter code to plasmids driven by a *f13* type PFam32 gene when and if the latter are found.

c. Plasmids cp32-2 and cp32-7 were given separate names before it was realized that they belong to the same compatibility group. We suggest that the name "cp32-2" no longer be used and "cp32-7" be used for plasmids found to have this type of PFam32 gene, since the B31 cp32-7 plasmid is completely sequenced and is thus better characterized.

d. We apply "Q" to circular cp32-10 type plasmids and "AD" to lp56 type plasmids that do not carry an integrated cp32-10. Such lp56 plasmids are present in *B. burgdorferi* strains 94a, WI91-23 and CA-11.2A [1].
   i. B31 lp56 genes were given locus_tags with the letter "Q".
   ii. The linear plasmid lp56 has a cp32-10 integrated into the PFam62/57 gene of its native partition gene cluster.
   iii. Thus, B31 lp56 has two different intact PFam32 genes (*q08* in its broken cluster and *q40* in the intact cluster of the integrated cp32-10); these belong to two different "compatibility types" of PFam32 genes. We therefore suspect that B31 lp56 partition is driven by the apparently intact cluster in the cp32-10 that includes PFam32 gene *q40*.
   iv. Hence, we give plasmids with B31 lp56 *q40* type PFam32 genes the locus_tag letter Q and plasmids with only the *q08* type PFam32 gene the letters "AD".

e. Plasmids with only cp32-13, lp28-8 and lp28-9 PFam32 genes are not present in the four strains studied in this report, but cp32-13 is known in strain CA-15 [2] as well as strains CA-11.2A, 118a and 72a [1] and lp28-8 and lp28-9 are known from the sequences of the genomes of *B. burgdorferi* strains 94a and Bol26, respectively [1].

f. Plasmids we name "lp32-3" are found in *B. burgdorferi* strains 72a and 118a [1]. Plasmids "lp32-6", "lp32-10" and "lp32-12" are known in isolates SV1, PKo (and ACA-1 and Far04) and SV1, resepectively (our unpublished results). These are all apparently linear plasmids that have significantly different genetic contents from the cp32s, but they carry a cp32-3, -6, -10 or -12 type PFam32 gene, respectively (no lp32 has been found in the same cell as a cp32 with the same putative PFam32 compatibility). We propose to name the locus tags for genes on these plasmids with the letters S, M, Q and X, which correspond to cp32-3, -6, -10 and -12, respectively.

References

1. Schutzer SE, Fraser-Liggett CM, Casjens SR, Qiu WG, Dunn JJ, *et al.* (2011) Whole genome sequences of thirteen isolates of *Borrelia burgdorferi*. J Bacteriol 193: 1018-1020.
2. Stevenson B, Miller JC (2003) Intra- and interbacterial genetic exchange of Lyme disease spirochete *erp* genes generates sequence identity amidst diversity. J Mol Evol 57: 309-324.
